# Supplementary material for: Sleep Counteracts Aging Phenotypes to Survive Starvation-Induced Developmental Arrest in C. elegans
Source: Curr Biol. 2018 Nov 19;28(22):3610–3624.e8. doi: 10.1016/j.cub.2018.10.009 (PMC6264389; doi:10.1016/j.cub.2018.10.009)
Supplement: Document S1. Figures S1–S6 and Tables S1–S7 [file mmc1.pdf]

Current Biology, Volume *28*

Supplemental Information

Sleep Counteracts Aging Phenotypes to Survive Starvation-Induced Developmental Arrest in *C. elegans*

Yin Wu, Florentin Masurat, Jasmin Preis, and Henrik Bringmann

Figure S1

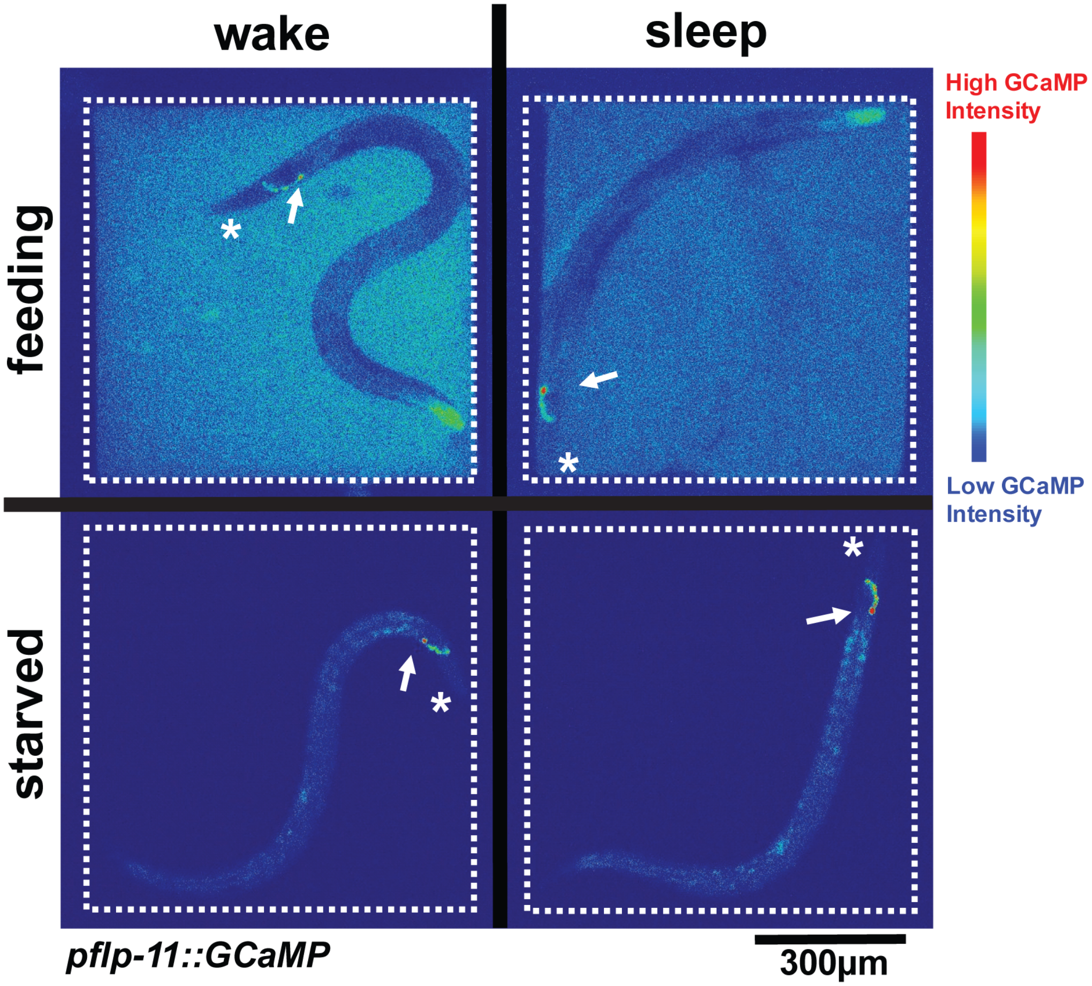

wake
sleep
feeding
starved
High GCaMP Intensity
Low GCaMP Intensity
pflp-11::GCaMP
300μm

Figure S1. Calcium and behavioral imaging in agarose microchambers. Related to Figure 1 and Figure 2. Shown are fluorescence images of adult worms expressing GCaMP3 in the sleep-active neuron RIS. The pictures are false-colored to visualize calcium activity. The border of the agarose microchamber is marked by a white dotted line. The asterisk indicates the worm's nose, the arrow the sleep neuron RIS. The left panels show wake worms, the right panels sleeping worms. Note the relaxed body posture and increased RIS GCaMP intensity during sleep. The top panels show worms feed on OP50. The bacteria are visible due to their auto-fluorescence. On the bottom panels, the worms are starved.

Figure S2

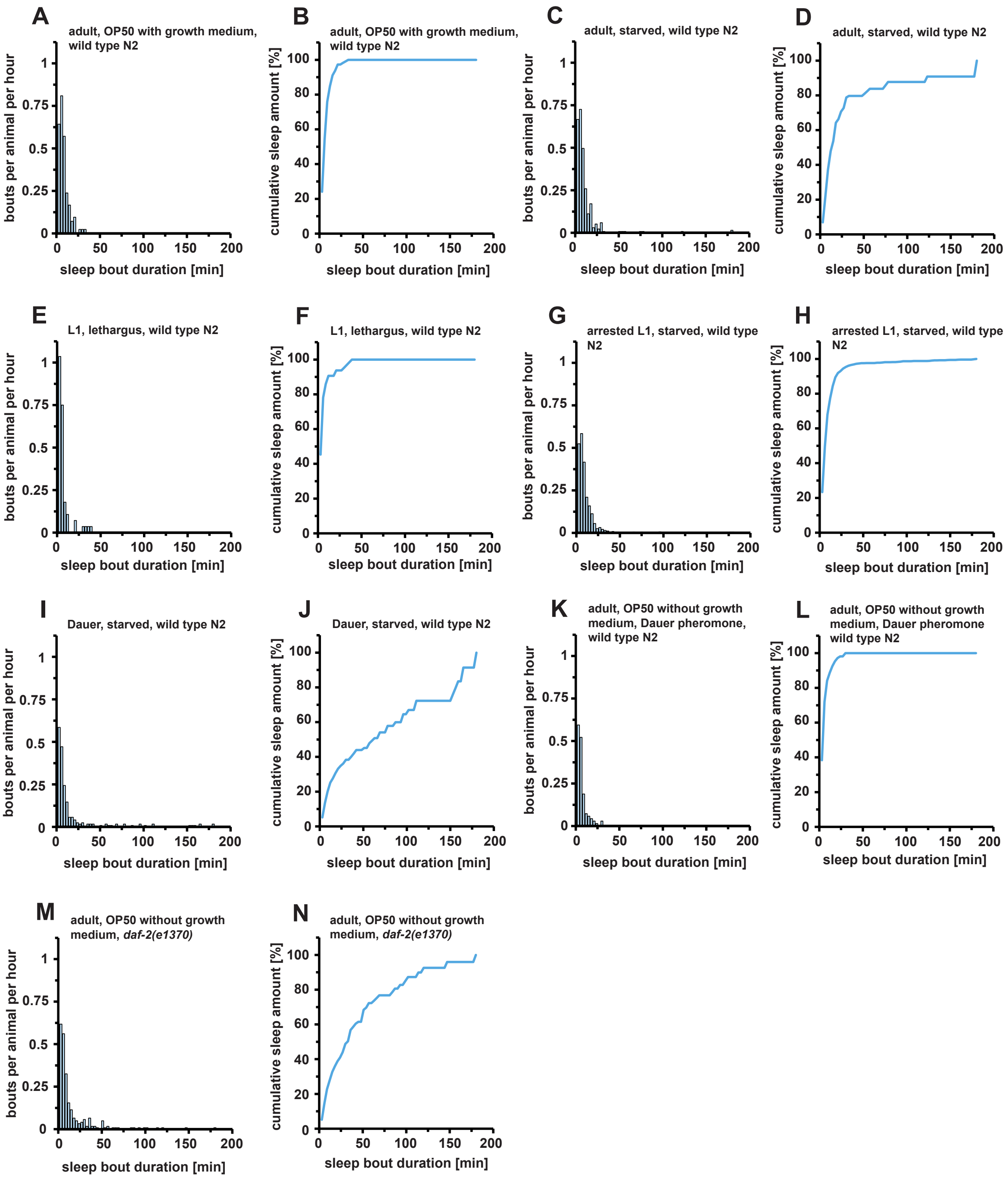
A
adult, OP50 with growth medium, wild type N2
bouts per animal per hour
sleep bout duration [min]
B
adult, OP50 with growth medium, wild type N2
cumulative sleep amount [%]
sleep bout duration [min]
C
adult, starved, wild type N2
bouts per animal per hour
sleep bout duration [min]
D
adult, starved, wild type N2
cumulative sleep amount [%]
sleep bout duration [min]
E
L1, lethargus, wild type N2
bouts per animal per hour
sleep bout duration [min]
F
L1, lethargus, wild type N2
cumulative sleep amount [%]
sleep bout duration [min]
G
arrested L1, starved, wild type N2
bouts per animal per hour
sleep bout duration [min]
H
arrested L1, starved, wild type N2
cumulative sleep amount [%]
sleep bout duration [min]
I
Dauer, starved, wild type N2
bouts per animal per hour
sleep bout duration [min]
J
Dauer, starved, wild type N2
cumulative sleep amount [%]
sleep bout duration [min]
K
adult, OP50 without growth medium, Dauer pheromone, wild type N2
bouts per animal per hour
sleep bout duration [min]
L
adult, OP50 without growth medium, Dauer pheromone wild type N2
cumulative sleep amount [%]
sleep bout duration [min]
M
adult, OP50 without growth medium, *daf-2(e1370)*
bouts per animal per hour
sleep bout duration [min]
N
adult, OP50 without growth medium, *daf-2(e1370)*
cumulative sleep amount [%]
sleep bout duration [min]

Figure S2. Distribution of sleep bout lengths and their contribution to total sleep amount. Related to Figure 1 and Figure 2. Shown are number of bouts per animal per hour as well as cumulative sleep amount as a function of bout length to show which bout lengths contribute how much to total sleep amount. Note that most sleep occurs in shorter bouts except for dauer larvae and *daf-2(-)*, which show also longer sleep bouts that contribute substantially to total sleep amount. (**A-B**) Adults with growth medium, (**C-D**) starved adults, (**E-F**) L1 lethargus, (**G-H**) starved L1 arrested, (**I-J**) dauer without food, (**K-L**) adults feeding on starved bacteria and dauer pheromone, (**M-N**) *daf-2(-)* adults feeding on starved bacteria.

Figure S3

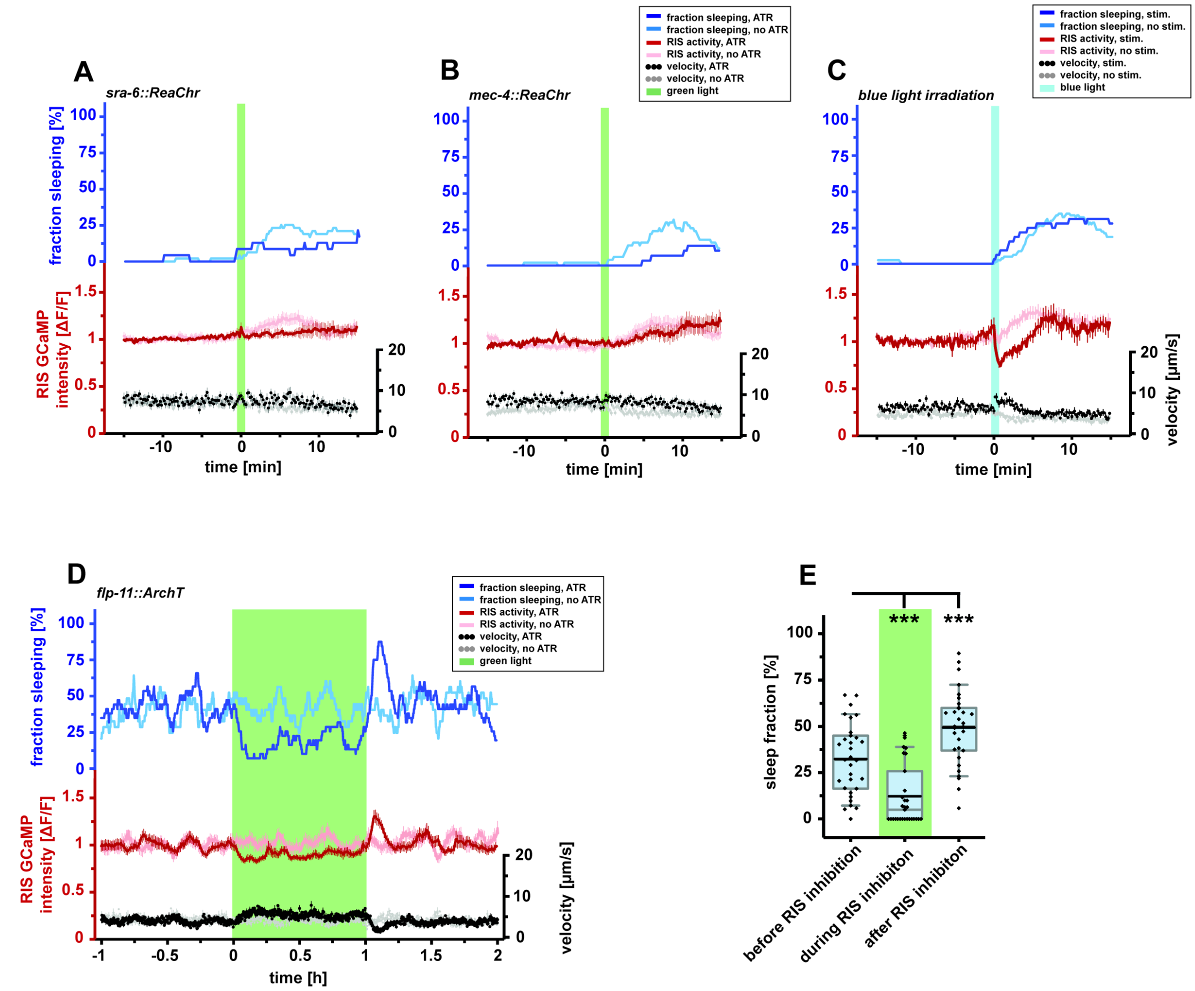
A
sra-6::ReaChr
B
mec-4::ReaChr
C
blue light irradiation
fraction sleeping, ATR
fraction sleeping, no ATR
RIS activity, ATR
RIS activity, no ATR
velocity, ATR
velocity, no ATR
green light
fraction sleeping, stim.
fraction sleeping, no stim.
RIS activity, stim.
RIS activity, no stim.
velocity, stim.
velocity, no stim.
blue light
fraction sleeping [%]
RIS GCaMP intensity [ΔF/F]
velocity [µm/s]
time [min]
D
flp-11::ArchT
time [h]
E
sleep fraction [%]
***
***
before RIS inhibition
during RIS inhibiton
after RIS inhibiton

Figure S3. Control experiments for characterizing larval sleep during starvation-induced arrest. Related to Figure 3. (**A-B**) ASH and mechano-sensory neurons activation during wake in arrested L1 larvae. During wake, there is no detectable action from ASH nociceptive neurons (**A**) or *mec-4* expression mechano-sensory neurons (**B**). RIS activity is shown in red (control without ATR in light red), speed in black (control without ATR in gray), and the fraction of sleeping animals in blue (control without ATR in light blue). (**A-C**) ASH and mechano-sensory neurons activation during wake in arrested L1 larvae. During wake, there is no detectable action from ASH nociceptive neurons (**A**), *mec-4* expression mechano-sensory neurons (**B**) or from noxious blue light irradiation (**C**). RIS activity is shown in red (control without ATR or without stimulation in light red), speed in black (control without ATR or without stimulation in gray), the fraction of sleeping animals in blue (control without ATR or without stimulation in light blue), green light illumination in green and noxious blue light irradiation in teal. (**D-E**) *flp-11::ArchT* activation efficiently reduces RIS activity and consequently inhibits sleep. (**D**) Complete documentation of homeostasis experiment. RIS activity is shown in red (control without ATR in light red), speed in black (control without ATR in gray), and the fraction of sleeping animals in blue (control without ATR in light blue). (**E**) Statistical comparison of the different 1h long periods. During green light illumination the sleep fraction was reduced by $62.0 \pm 0.5\%$, $***p < 0.001$, and after illumination the sleep fraction increased by $52.5 \pm 0.1\%$, $***p < 0.001$, compared to the sleep fraction before the illumination. Comparisons were made using the paired Wilcoxon rank test. $n = 32$ worms.

Figure S4

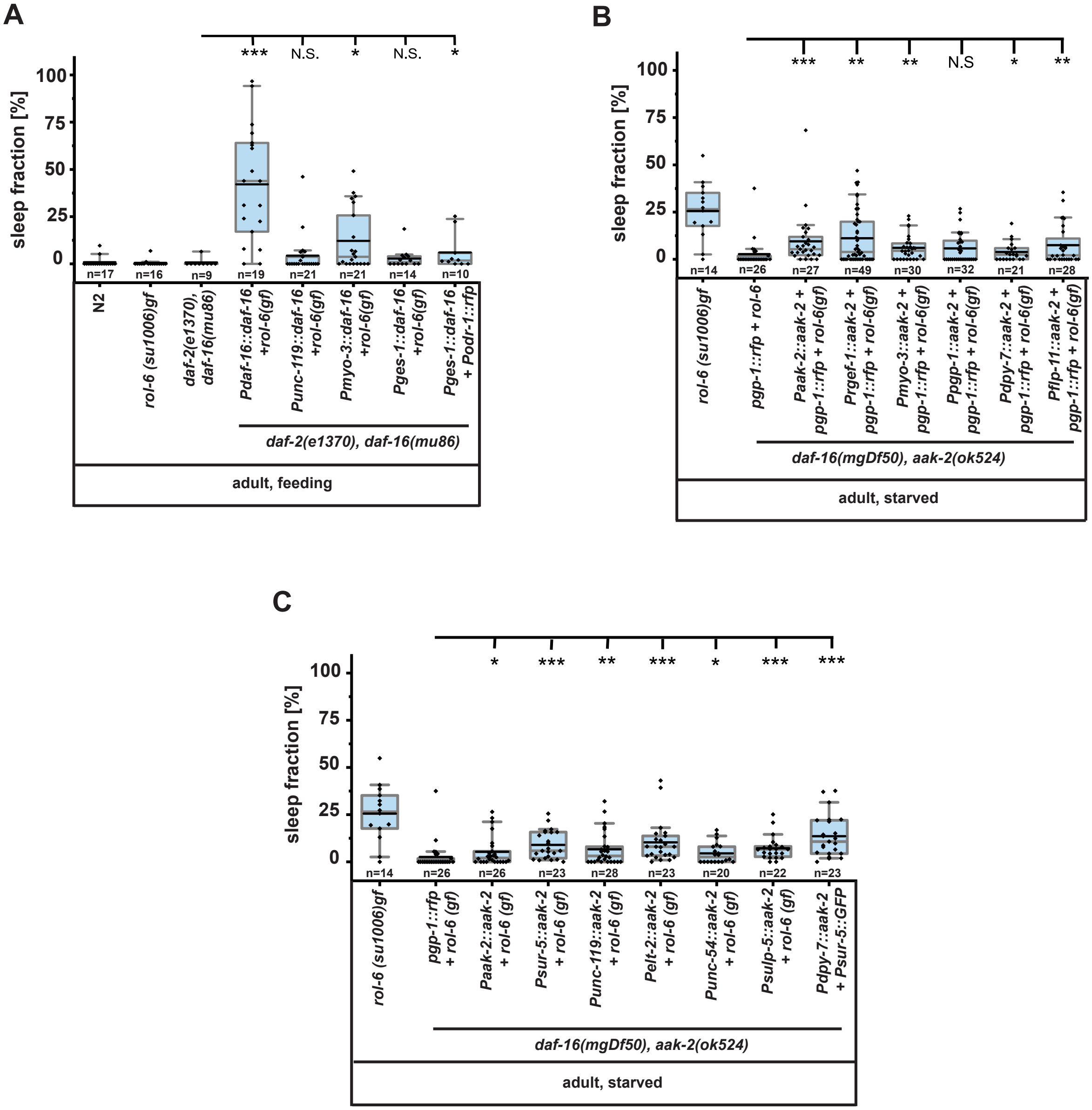

Figure S4. Rescue experiments for FoxO and AMPK. Related to Figure 4. (A) Rescue experiments using inserted transgenes of *daf-16* shows weak rescue only in muscle and inconsistent recue in the intestine. *daf-16* was expressed from its endogenous promoter, in neurons (*Punc-119*), in body wall muscle (*Pmyo-3*) and in the intestine (*Pges-1*). Most transgenes carry a *rol-6(su1006)* transformation marker, the roller phenotype does not affect sleep, Median sleep fraction was 0% for wild type N2 and *rol-6(su1006)*, the same data is displayed in panel B. Median sleep fraction for rescue experiments was 0% for the background genotype *daf-2(e1370)/daf-16(mu86)*, 44% for rescue using the endogenous promoter (***p < 0.001), 0 % for pan-neuronal rescue, 4% for muscle rescue (*p < 0.05), 1% and 2% for intestinal rescue dependent on the

transgenic array ($p = 0.11$ and $*p < 0.05$, respectively). *daf-2(e1370)/daf-16(mu86)* data was taken from DIC movies collected for the same animals that were analyzed for Figure 4C and is the same as used in Figure S4B. (B) Rescue experiments using extrachromosomal transgenes of *aak-2* shows weak rescue across tissues, which was expressed from its endogenous promotor, in neurons (*Prgef-1*), in body wall muscle (*Pmyo-3*), in the intestine (*Ppgp-1*), in the hypodermis (*Pdpy-7*), and in RIS (*Pflp-11*). Transgenes carry a *rol-6(su1006)* and intestinal red fluorescence (*pgp-1::rfp*) transformation marker. For comparison, a wild type background is shown, which only expresses a transformation marker. It is the same comparison as shown in Figure S4D. Median sleep fraction was 0% for *daf-2(mgDf50)/aak-2(ok524)* carrying the transformation markers only, 5% for rescue by endogenous promoter ($***p < 0.001$), 4% for pan-neuronal rescue ($**p < 0.01$), 4% for muscle rescue ($**p < 0.01$), 0% for intestine, 2% for hypodermis ($*p < 0.05$), and 2% for RIS ($**p < 0.01$). (C) Additional rescue experiments using extrachromosomal transgenes of *aak-2* with different transformation markers and promoters shows weak rescue across tissues, which was expressed from its endogenous promotor, ubiquitously (*Psur-5*), in neurons (*Punc-119*), in the intestine (*Pelt-2*), in muscle (*Punc-54*), in the hypodermis (*Pdpy-7*), and in the excretory cell (*Psulp-5*). Transgenes carry a *rol-6(su1006)* or other transformation marker. For comparison, a wild type background is shown, which only expresses a transformation marker. It is the same comparison as shown in Figure S4C. Median sleep fraction was 0% for *daf-2(mgDf50)/aak-2(ok524)* carrying the transformation markers only, 2% for rescue by the endogenous promoter ($*p < 0.001$), 6% for ubiquitous expression ($***p < 0.001$), 3% for pan-neuronal rescue ($**p < 0.01$), 8% for intestine ($***p < 0.001$), 3% for muscle rescue ($*p < 0.05$), 7% for the excretory cell ($***p < 0.001$) and 11% for hypodermis ($***p < 0.001$). The low rescue effects obtained after using the endogenous *aak-2* and *sur*-5 promoters are consistent with previous measurements using these transgenes. Perhaps the weak rescue can be explained by weak transmission of the array[S1]. The number of worms assayed (n) is displayed underneath each box plot. For statistical comparisons, Mann-Whitney U test was used to calculate the p value and significance was confirmed by Benjamini-Hochberg Procedure for multiple comparisons.

Figure S5

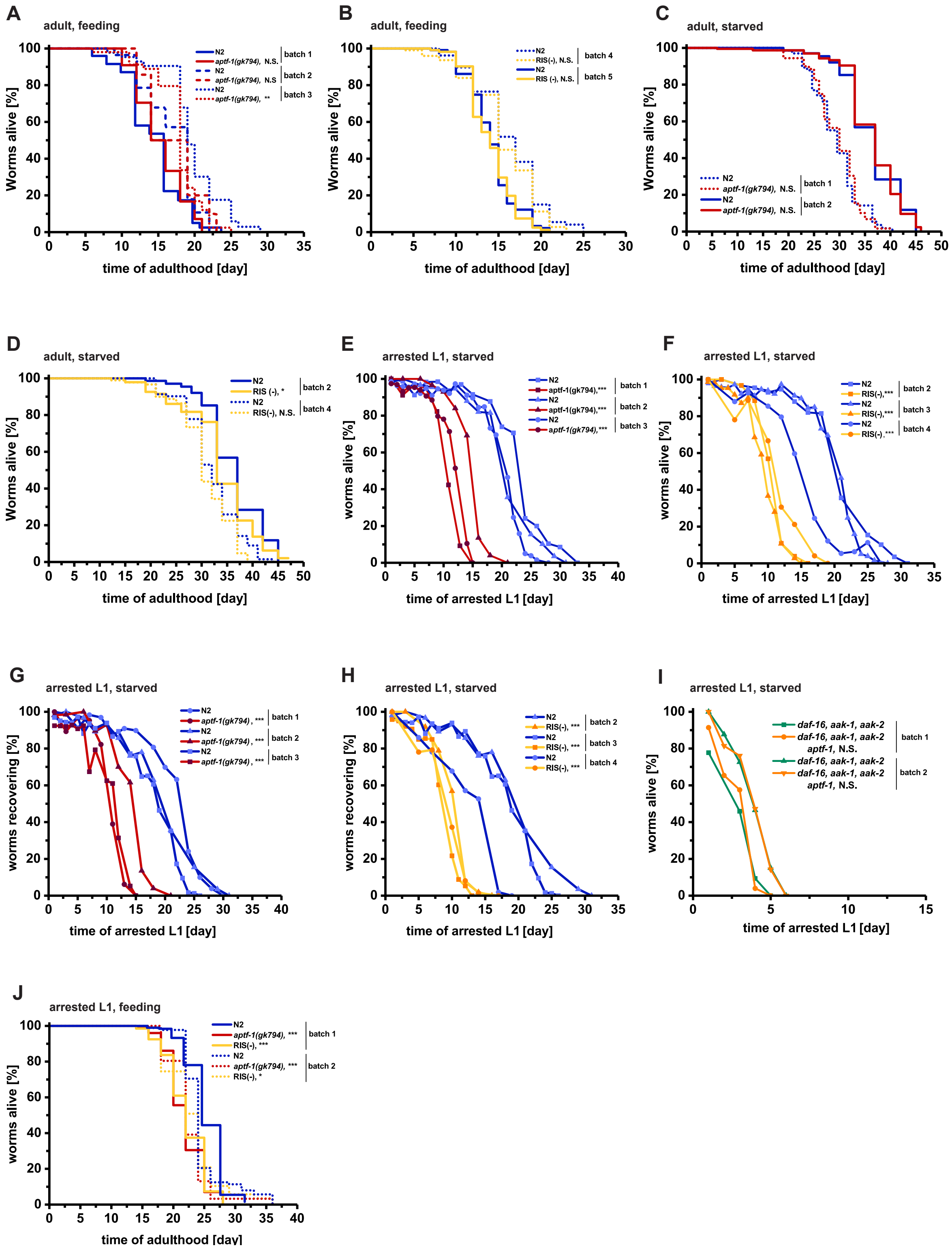

A
adult, feeding
B
adult, feeding
C
adult, starved
D
adult, starved
E
arrested L1, starved
F
arrested L1, starved
G
arrested L1, starved
H
arrested L1, starved
I
arrested L1, starved
J
arrested L1, feeding
Worms alive [%]
worms alive [%]
worms recovering [%]
time of adulthood [day]
time of arrested L1 [day]
N2
aptf-1(gk794), N.S.
aptf-1(gk794), **
aptf-1(gk794), ***
RIS(-), N.S.
RIS (-), *
RIS(-), ***
RIS(-), *
daf-16, aak-1, aak-2
daf-16, aak-1, aak-2 aptf-1, N.S.
batch 1
batch 2
batch 3
batch 4
batch 5

Figure S5. Sleep is required to survive larval starvation. Related to Figure 5. Supplementary figure containing additional replicates related to Figure 5. The biological replicates are indicated as different batches with different lines or symbols. **(A)** Adult lifespan of feeding wild type (blue) and *aptf-1* mutants (red). No consistent significant difference could be shown. **(B)** Lifespan of feeding wild type (blue) and RIS(-) (yellow) adult worms were not significantly different for the two replicates. **(C)** No significant difference was detected for the starved adult lifespan of wild type and *aptf-1(gk794),* **(D)** and also not between starved wild type and RIS(-) adult worms. **(E-F)** L1-arrested starved *aptf-1(gk794)* mutants and RIS ablated worms show substantially reduced survival in all replicates latest from day 16. **(G-H)** All replicates of arrested L1 *aptf-1(gk794)* and RIS-ablated worms showed a significant decline in the ability to re-enter development compared to N2 worms when fed. Data are significant latest from day 12. **(I)** Starved arrested L1 *aak-1(tm1944)/aak-2(ok524)/daf-16(mgDf50)/aptf-1(gk794)* quadruple mutants showed no reduced lifespan compared to *aak-1(tm1944)/aak-2(ok524)/daf-16(mgDf50)* triple mutants. **(J)** *aptf-1(gk794)* and RIS(-) L1s arrested on FUdR have a decreased survival compared to wild type worms in the presence of food. The corresponding data, significance values and tests for all replicates are shown in Tables S1-S5.

Figure S6

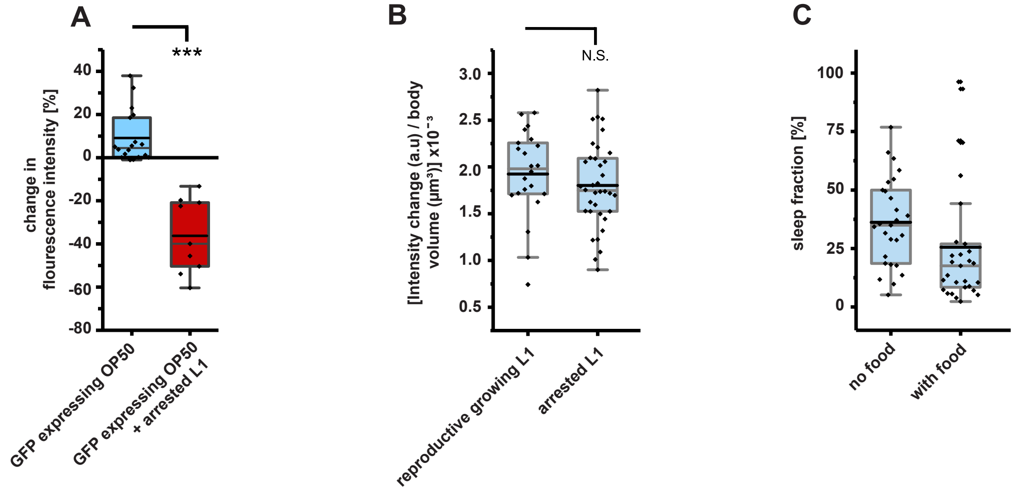

Figure S6. Arrested L1 larvae consume bacteria. Related to Figure 5. (**A**) The fluorescence intensity change of GFP expressing OP50 was measured in agarose microchambers. The two-day reduction in fluorescence is significantly different comparing microchambers containing arrest L1s to microchambers without worms (with worms: 36.3 ± 5.8% decrease, n = 9 microchambers, without worms: 9.1 ± 2.8% increase, n = 18 microchambers, ***$p < 0.001$, Mann-Whitney U test). **(B)** Arrested L1 larvae consume similar amounts of bacteria as reproductive growing L1 worms. The change in fluorescence intensity of OP50 GFP bacteria within 200 min was normalized to worm volume, to compensate for variability in size, which is caused by increased growth in non-arrested worms. The time window was chosen because size differences were still modest. No significant difference in bacterial consumption was detected for reproductive growing L1s (with a mean of 1.92×10^{-3} arbitrary units/μm^3, n = 22) and FUdR-arrested L1s (mean = 1.8×10^{-3} arbitrary units /µm3, n = 36), p = 0.33, two-sample t-test. (C) Sleep fraction of starved and feeding FUdR arrested L1s in wildtype. Starved L1s with FUdR (4 to 5 days-old) had a median sleep fraction of 34.9%, n = 27. Median sleep fraction of the feeding L1s with FUdR (4 to 5 days-old) is 17.5%, n = 29.

Table S1

Lifespan of adult, feeding					
Strain	Experiment batch (in Figure)	# scored/ # total	Mean of lifespan (days)	Compared to	*p* value against control (log-rank test)
wildtype N2	1 (Figure 5A)	44/50	14.64		
aptf-1(gk794)	1 (Figure 5A)	43/50	15.29	N2 Batch1	0.71, N.S.
wildtype N2	2	28/50	16.92		
aptf-1(gk794)	2	12/50	16.91	N2 Batch2	0.70, N.S.
wildtype N2	3	39/50	19.74		
aptf-1(gk794)	3	42/50	17.82	N2 Batch3	0.003, **
wildtype N2	4	72/100	16.30		
RIS(-)	4	75/102	15.54	N2 Batch4	0.23, N.S.
wildtype N2	5 (Figure 5B)	94/120	14.22		
RIS(-)	5 (Figure 5B)	86/120	14.07	N2 Batch5	0.71, N.S.
Lifespan of adult, starved					
wildtype N2	1 (Figure 5C)	60/100	29.81	-	
aptf-1(gk794)	1 (Figure 5C)	63/100	29.46	N2 Batch1	0.65, N.S.
wildtype N2	2	52/200	36.21		
aptf-1(gk794)	2	94/200	36.33	N2 Batch2	0.96, N.S.
RIS(-)	2	83/200	33.62	N2 Batch2	0.04, *
wildtype N2	3 (Figure 5D)	85/100	31.96		
RIS(-)	3 (Figure 5D)	84/95	30.64	N2 Batch3	0.06, N.S.

Table S1. Overview of all replicates of the adult lifespan. Related to Figure 5. Displayed are the strains used, the experimental / replicates, the scored animals from the total number (the missing worms were censored), the mean lifespan estimated from the log-rank test, against which wild type data was tested and the *p* value of the log-rank test.

Table S2

Survival of arrested L1, starved						
Strain	Experiment batch (in Figure)	min. scored/counting	50% alive at (days)	Compared to	Δ 50% alive	*p* value against control (Fisher's exact test)
wildtype N2	1	>50	22.89			
aptf-1(gk794)	1	>50	10.62	N2 Batch1	-53.6%	< 0.001, *** from day 11
wildtype N2	2	>50	20.06			
aptf-1(gk794)	2	>50	14,69	N2 Batch2	-26.8%	< 0.001, *** from day 16
RIS(-)	2	>50	10.3	N2 Batch2	-48.7%	< 0.001, *** from day 10
wildtype N2	3 (Figure 5E)	>50	20.70			
aptf-1(gk794)	3 (Figure 5E)	>50	12.07	N2 Batch3	-41.7%	< 0.001, *** from day 11
RIS(-)	3 (Figure 5E)	>50	9.41	N2 Batch3	-54.5%	< 0.001, *** from day 9
wildtype N2	4	>50	14.85			
RIS(-)	4	>50	9.93	N2 Batch4	-33.1%	< 0.001, *** from day 12

Table S2. Overview of all replicates of the starved arrested L1 lifespans. Related to Figure 5. Displayed are the strains used, the batch / replicate, the scored worms, median survival, against which wild type data was tested, the reduction in lifespan compared to wildtype and the *p* value and from which day on there was a significant difference in the Fisher's exact test.

Table S3

Ability to re-enter development of arrested L1 when fed, starved						
Strain	Experiment batch (in Figure)	min. scored/counting	50% recover at (days)	Compared to	Δ 50% alive	*p* value against control (Fisher's exact test)
wildtype N2	1	>50	22.68			
aptf-1(gk794)	1	>50	10.49	N2 Batch1	-53.7%	< 0.001, *** from day 11
wildtype N2	2	>50	19.42			
aptf-1(gk794)	2	>50	14.48	N2 Batch2	-25.4%	< 0.001, *** from day 16
RIS(-)	2	>50	10.28	N2 Batch2	-47.1%	< 0.001, *** from day 10
wildtype N2	3 (Figure 5F)	>50	18.74			
aptf-1(gk794)	3 (Figure 5F)	>50	11.37	N2 Batch3	-39.3%	< 0.001, *** from day 12
RIS(-)	3 (Figure 5F)	>50	8.53	N2 Batch3	-54.5%	< 0.001, *** from day 8
wildtype N2	4	>50	14.12			
RIS(-)	4	>50	9.09	N2 Batch4	-35.6%	< 0.001, *** from day 12

Table S3. Overview of all replicates of the capacity to recover from the L1 arrest. Related to Figure 5. Displayed are the strains used, the experimental batch / replicate, the scored animals, median recovery rate, against which wild type data set was tested, the reduction in lifespan compared to wildtype and the *p* value and from which day on there was a significant difference in the Fisher's exact test.

Table S4

Survival of arrested L1 of quadruple mutants, starved					
Strain	Experiment batch (in Figure)	min. scored/counting	50% alive at (days)	Compared to	*p* value against control (Fisher's exact test)
aak-1(tm1944), aak-2(ok524), daf-16(mgDf50)	1	>60	2.74		
aak-1(tm1944), aak-2(ok524), daf-16(mgDf50), aptf-1(gk794)	1	>60	3.14	Triple mutant Batch1	>0.05 N.S.
aak-1(tm1944), aak-2(ok524), daf-16(mgDf50)	2 (Figure 5H)	>60	3.85		
aak-1(tm1944), aak-2(ok524), daf-16(mgDf50), aptf-1(gk794)	2 (Figure 5H)	>60	3.9	Triple mutant Batch2	>0.05 N.S.

Table S4. Overview of all replicates of the lifespan of IIS and AMPK mutants. Related to Figure 5. Overview of all replicates of the lifespan of IIS and AMPK mutants. Displayed are the strains used, the experimental batch / replicate, the scored animals, median recovery rate, against which wild type data set was tested, the reduction in lifespan compared to wildtype and the *p* values and significance in the Fisher's exact test.

Table S5

Survival of FUdR-arrested L1, feeding						
Strain	Experiment batch (in Figure)	scored/total	Mean of lifespan (days)	Compared to	Δ 50% alive	*p* value against control (log-rank test)
wildtype N2	1 (Figure 5I)	143/200	25.70			
aptf-1(gk794)	1 (Figure 5I)	140/200	21.86	N2 Batch1	-14.5%	< 0.001, ***
RIS(-)	1 (Figure 5I)	135/200	22.05	N2 Batch1	-14.2%	< 0.001, ***
wildtype N2	2	88/101	24.63			
aptf-1(gk794)	2	92/101	22.5	N2 Batch2	-8.6%	< 0.001, ***
RIS(-)	2	100/144	23.04	N2 Batch2	-6.5%	0.017, *

Table S5. Overview of all replicates of the lifespan of FUdR-arrested L1s. Related to Figure 5. Displayed are the strains used, the experimental batch / replicate, the scored animals, the mean survival, against which wild type data set was tested, the reduction in lifespan compared to wildtype and the *p* values and the significance in the log-rank test.

Table S6

Experiment	No. of biological replicates	Comments
Figure 1J	4	
Figure 1K	7	
Figure 1L	3	
Figure 1M	7	
Figure 1N	5	
Figure 1O	3	
Figure 2A, wt with growth medium	4	
Figure 2A, *aptf-1(-)* with growth medium	4	
Figure 2A, wt without growth medium	2	
Figure 2A, *aptf-1(-)* without growth medium	2	
Figure 2A, wt with growth medium, dead OP50	3	
Figure 2A, *aptf-1(-)* with growth medium, dead OP50	2	
Figure 2B, wt, adult feeding	2	
Figure 2B, *aptf-1(-)*, adult feeding	2	
Figure 2B, *Pflp-11:EGL-1*, adult feeding	6	
Figure 2B, wt, adult starved	9	
Figure 2B, *aptf-1(-)*, adult starved	3	
Figure 2B, *Pflp-11:EGL-1*, adult starved	6	
Figure 2C, wt, non-starved L1, before lethargus	3	
Figure 2C, *aptf-1(-)*, non-starved L1, before lethargus	3	
Figure 2C, wt, non-starved L1, during lethargus	3	
Figure 2C, *aptf-1(-)*, non-starved L1, during lethargus	3	
Figure 2D, wt, arrested L1	8	
Figure 2D, *aptf-1(-)*, arrested L1	4	
Figure 2D, *Pflp-11:EGL-1*, arrested L1	2	
Figure 2E, wt, Dauer	8	
Figure 2E, *aptf-1(-)*, Dauer	4	
Figure 2E, *Pflp-11:EGL-1*, Dauer	4	
Figure 2F, wt, Dauer Pheromone	5	
Figure 2F, *aptf-1(-)*, Dauer Pheromone	3	
Figure 3A, B: 2	2	
Figure 3C with ATR	2	
Figure 3C without ATR	2	
Figure 3D with ATR	2	
Figure 3D without ATR	2	
Figure 3E with ATR	4	
Figure 3E without ATR	3	
Figure 3F	2	
Figure 4B	6	
Figure 4C wt	2	
Figure 4C *daf-2(-)*	9	
Figure 4C *daf-2(-); aptf-1(-)*	4	
Figure 4C *daf-2(-); daf-16(-)*	6	
Figure 4D wt	9	
Figure 4D *daf-16(-)*	4	
Figure 4D *aak-1(-); aak-2(-)*	7	
Figure 4D *daf-16(-); aak-1(-)*	4	
Figure 4D *daf-16(-); aak-2(-)*	2	
Figure 4D *daf-16(-); aak-1(-); aak-2(-)*	3	
Figure 4E wt	8	
Figure 4E *daf-16(-)*	2	
Figure 4E *aak-1(-); aak-2(-)*	2	
Figure 4E *daf-16(-); aak-1(-)*	2	
Figure 4E *daf-16(-); aak-2(-)*	4	
Figure 4E *daf-16(-); aak-1(-); aak-2(-)*	6	
Figure 4F wt	2	
Figure 4F *daf-18(-)*	2	
Figure 4F *sir2.1(-)*	2	
Figure 5A adult wt, feeding + Figure S5A	3	
Figure 5A adult *aptf-1(-)*, feeding + Figure S5A	3	
Figure 5B adult wt, feeding + Figure S5B	2	
Figure 5B adult RIS(-), feeding + Figure S5B	2	
Figure 5C adult wt, starved + Figure S5C	2	
Figure 5C adult *aptf-1(-)*, starved + Figure S5C	2	
Figure 5D adult wt, starved + Figure S5D	2	
Figure 5D adult RIS(-), starved + Figure S5D	2	
Figure 5E L1 wt, starved + Figure S5E-F	3	

Figure 5E L1 *aptf-1(-)*, starved + Figure S5E-F	3	
Figure 5E L1 RIS(-), starved + Figure S5E-F	3	
Figure 5F L1 wt, starved + Figure S5G-H	3	
Figure 5F L1 *aptf-1(-)*, starved + Figure S5G-H	3	
Figure 5F L1 RIS(-), starved + Figure S5G-H	3	
Figure 5G wt, starved	5	
Figure 5H *daf-16(-); aak-1(-); aak-2(-),* starved + Figure S5I	2	
Figure 5H *daf-16(-), aak-1(-), aak-2(-), aptf-1(-),* starved + Figure S5I	2	
Figure 5I L1 wt, FUDR, feeding + Figure S5J	2	
Figure 5I L1 *aptf-1(-)*, FUDR, feeding + Figure S5J	2	
Figure 5I L1 RIS(-), FUDR, feeding + Figure S5J	2	
Figure 6A wt	2	
Figure 6A *aptf-1(-)*	2	
Figure 6C wt	2	
Figure 6C *aptf-1(-)*	2	
Figure 6E wt	2	
Figure 6E *aptf-1(-)*	2	
Figure S2A	4	
Figure S2B	4	
Figure S2C	9	
Figure S2D	9	
Figure S2E	3	
Figure S2F	3	
Figure S2G	8	
Figure S2H	8	
Figure S2I	8	
Figure S2J	8	
Figure S2K	5	
Figure S2L	5	
Figure S2M	9	
Figure S2N	9	
Figure S3A with ATR	2	
Figure S3A without ATR	2	
Figure S3B with ATR	2	
Figure S3B without ATR	2	
Figure S3C, D with ATR	4	
Figure S3C, D without ATR	3	
Figure S3E, before RIS inhibition	4	
Figure S3E, during RIS inhibition	4	
Figure S3E, after RIS inhibition	4	
Figure S4A wt	2	From Figure 4C
Figure S4A *rol-6(gf)*	2	
Figure S4A *daf-2(-); daf-16(-)*	4	Reanalysis of DIC movies from Figure 4C
Figure S4A *daf-2(-); daf-16(-); Pdaf-16::daf-16+rol-6(gf)*	3	
Figure S4A *daf-2(-); daf-16(-); Punc-119::daf-16+rol-6(gf)*	5	
Figure S4A *daf-2(-); daf-16(-); Pmyo-3::daf-16+rol-6(gf)*	5	
Figure S4A *daf-2(-); daf-16(-); Pges-1::daf-16+rol-6(gf)*	4	
Figure S4B *rol-6(gf)*	2	
Figure S4B *daf-16(-); aak-2(-); pgp-1::rfp+rol-6 (gf)*	4	
Figure S4B*daf-16(-); aak-2(-); Paak-2::aak-2+pgp-1::rfp+rol-6 (gf)*	5	
Figure S4B *daf-16(-); aak-2(-); Prgef-1::aak-2+pgp-1::rfp+rol-6 (gf)*	8	
Figure S4B*daf-16(-); aak-2(-); Pmyo-3::aak-2+pgp-1::rfp+rol-6 (gf)*	5	
Figure S4B *daf-16(-); aak-2(-); Ppgp-1::aak-2+pgp-1::rfp+rol-6 (gf)*	5	
Figure S4B *daf-16(-); aak-(-); Pdpy-7::aak-2+pgp-1::rfp+rol-6 (gf)*	3	
Figure S4B *daf-16(-); aak-2(-); Pdpy-7::aak-2+pgp-1::rfp+rol-6 (gf)*	3	
Figure S4C *rol-6 (gf)*	2	
Figure S4C *daf-16(-); aak-2(-); pgp-1::rfp+rol-6 (gf)*	4	
Figure S4C *daf-16(-); aak-2(-); Paak-2::aak-2+rol-6 (gf)*	3	
Figure S4C *daf-16(-); aak-2(-); Psur-5::aak-2+rol-6 (gf)*	3	
Figure S4C *daf-16(-), aak-2(-), Punc-119::aak-2+rol-6 (gf)*	2	
Figure S4C *daf-16(-); aak-2(-); Pelt-2::aak-2+rol-6 (gf)*	3	
Figure S4C *daf-16(-); aak-2(-); Punc-54::aak-2+rol-6 (gf)*	3	
Figure S4C *daf-16(-); aak-2(-); Psulp-5::aak-2+rol-6 (gf)*	3	
Figure S4C *daf-16(-); aak-2(-); Pdpy-7::aak-2+Psur-5::GFP*	3	
Figure S5A, wt, adult feeding	3	
Figure S5A, *aptf-1*(-), adult feeding	3	
Figure S5B, wt, adult feeding	2	
Figure S5B, RIS(-), adult feeding	2	
Figure S5C, wt, adult starved	2	

Figure S5C, *aptf-1(-)*, adult starved	2	
Figure S5D, wt, adult starved	2	
Figure S5D, RIS(-), adult starved	2	
Figure S5E, wt, arrested L1 starved	3	
Figure S5E, *aptf-1(-)*, arrested L1 starved	3	
Figure S5F, wt, arrested L1 starved	3	
Figure S5F, RIS(-), arrested L1 starved	3	
Figure S5G, wt, arrested L1 starved	3	
Figure S5G, *aptf-1(-)*, arrested L1 starved	3	
Figure S5H, wt, arrested L1	3	
Figure S5H, RIS(-), arrested L1	3	
Figure S5I, *daf-16(-); aak-1(-); aak-2(-),* arrested L1	2	
Figure S5I, *daf-16(-); aak-1(-); aak-2(-); aptf-1(-)*, arrested L1	2	
Figure S5J, wt, arrested L1 feeding	2	
Figure S5J, *aptf-1(-)*, arrested L1 feeding	2	
Figure S5J, RIS(-), arrested L1 feeding	2	
Figure S6A GPF OP50 control	2	
Figure S6A GFP OP50 L1 arrested worm	2	
Figure S6B reproductive growing L1	2	
Figure S6B arrested L1	2	
Figure S6C FUDR arrested L1, starved	2	
Figure S6C FUDR arrested L1, feeding	2	

Table S6. Number of replicates for all experiments. Related to all Figures and Supplemental Figures.

Table S7

Primer name	Primer sequence 5'-3'
aak-1(tm1944)	
890	ACTGTTCCGAGTCCTTTCATTC
891	TTCCCTTTCTTCGCTCACTTTT
892	ATGGAACTGTGTATCGGCCA
aak-2(ok524)	
893	ATCGCCAAATTATGCTGCCC
894	GGCAGGGTTCCACAAAGAAG
895	AAGGAGACACTCGGAGTTGG
aptf-1(ok974)	
48	CGACAATCTTCCCAAAGACC
49	CGGATCGATTGCTAGAGAGG
50	GCTTGGACGGCTTTAGTTGA
*daf-2(e1370)**	
519	CGGGATGAGACTGTCAAGATTGGAGATTTCGG
520	CAACACCTCATCATTACTCAAACCAATCCATG
daf-16(mgDf50)	
636	AGGTCAGAGTTCTAGGCGTAAA
637	CGGTTCCAGCAATTCCAAGT
638	AGTGGATTCTGAGCACACGA
daf-16(mu86)	
468	AAAGAGCTCGTGGTGGGTTA
469	TCGCGCCTTTGTCTCTCTAT
470	AGTGTCGAGTGAAGGGAGC
sir-2.1(ok434)	
1107	GTTTGATCACTCCTTCGCAAC
1108	TCCCAGGACAGTTCGTACC
1109	GCGGAAATGGCAGAAGTAGT
daf-18(ok480)	
1090	AGCATGGACTTCAACGATACAA
1091	GAATGCCAGATTGCCGAACA
1092	CGGTGGTCCATTTGAGATACC

Table S7. List of PCR primers used for Duplex PCR Genotyping. Related to STAR Methods. *The *daf-2* mutant allele was identified using restriction with NcoI-HF (in CutSmart Buffer, New England Biolabs).

Supplemental Reference

S1. Narbonne, P., and Roy, R. (2009). Caenorhabditis elegans dauers need LKB1/AMPK to ration lipid reserves and ensure long-term survival. Nature *457*, 210-214.
