# Supplementary material for: Sleep Counteracts Aging Phenotypes to Survive Starvation-Induced Developmental Arrest in C. elegans
Source: Curr Biol. 2018 Nov 19;28(22):3610–3624.e8. doi: 10.1016/j.cub.2018.10.009 (PMC6264389; doi:10.1016/j.cub.2018.10.009)
Supplement: Document S2. Article plus Supplemental Information [file mmc2.pdf]

Current Biology

Article

Sleep Counteracts Aging Phenotypes to Survive Starvation-Induced Developmental Arrest in *C. elegans*

Graphical Abstract

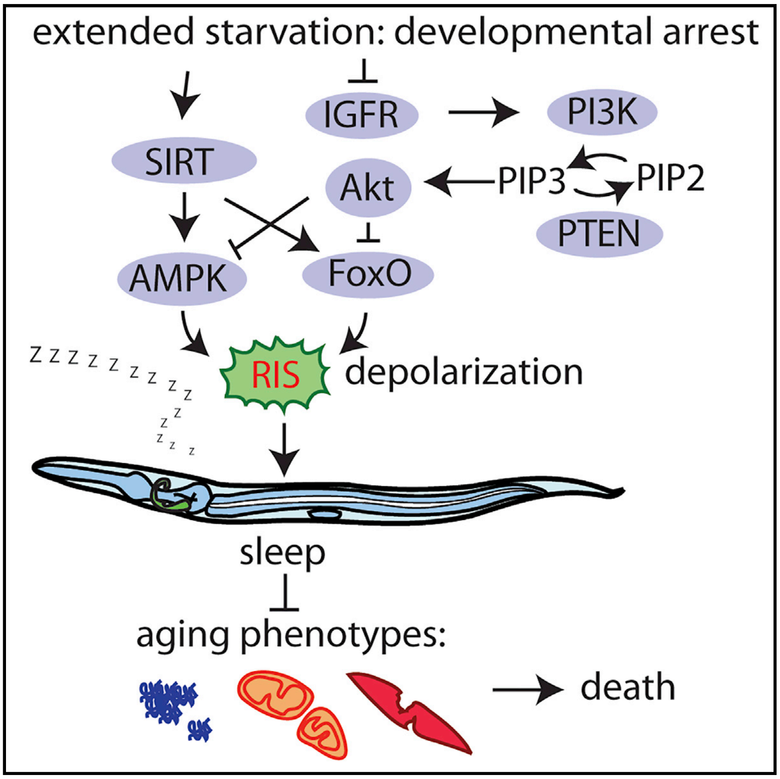

Highlights

- *C. elegans* sleep across most physiological conditions, including developmental arrest
- The sleep-active RIS neuron generally induces physiological sleep
- Insulin and sirtuin signaling control AMPK and FoxO to induce sleep during starvation
- Sleep is required to survive developmental arrest and counteracts aging phenotypes

Authors

Yin Wu, Florentin Masurat, Jasmin Preis, Henrik Bringmann

Correspondence

henrik.bringmann@mpibpc.mpg.de

In Brief

Wu, Masurat, et al. show that *C. elegans* can sleep during most conditions and stages of their life. Worms sleep prominently during developmental arrest induced by starvation. A conserved aging gene network controls sleep through a sleep-active neuron, whose activity is required to slow the progression of aging phenotypes, thus allowing survival.

Wu et al., 2018, Current Biology *28*, 3610–3624
November 19, 2018 © 2018 The Authors. Published by Elsevier Ltd.
https://doi.org/10.1016/j.cub.2018.10.009

CellPress

CellPress

Current Biology
Article

Sleep Counteracts Aging Phenotypes to Survive Starvation-Induced Developmental Arrest in *C. elegans*

Yin Wu,[1,2] Florentin Masurat,[1,2] Jasmin Preis,[1] and Henrik Bringmann[1,3,*]
[1]Max Planck Institute for Biophysical Chemistry, Am Fassberg 11, 37077 Göttingen, Germany
[2]These authors contributed equally
[3]Lead Contact
*Correspondence: henrik.bringmann@mpibpc.mpg.de
https://doi.org/10.1016/j.cub.2018.10.009

SUMMARY

Sleep is ancient and fulfills higher brain functions as well as basic vital processes. Little is known about how sleep emerged in evolution and what essential functions it was selected for. Here, we investigated sleep in *Caenorhabditis elegans* across developmental stages and physiological conditions to find out when and how sleep in a simple animal becomes essential for survival. We found that sleep in worms occurs during most stages and physiological conditions and is typically induced by the sleep-active RIS neuron. Food quality and availability determine sleep amount. Extended starvation, which induces developmental arrest in larvae, presents a major sleep trigger. Conserved nutrient-sensing regulators of longevity and developmental arrest, AMP-activated kinase and FoxO, act in parallel to induce sleep during extended food deprivation. These metabolic factors can act in multiple tissues to signal starvation to RIS. Although sleep does not appear to be essential for a normal adult lifespan, it is crucial for survival of starvation-induced developmental arrest in larvae. Rather than merely saving energy for later use, sleep counteracts the progression of aging phenotypes, perhaps by allocating resources. Thus, sleep presents a protective anti-aging program that is induced by nutrient-sensing longevity pathways to survive starvation-induced developmental arrest. All organisms are threatened with the possibility of experienced famine in their life, which suggests that the molecular coupling of starvation, development, aging, and sleep was selected for early in the evolution of nervous systems and may be conserved in other species, including humans.

INTRODUCTION

Sleep supports higher brain functions such as memory consolidation and synaptic plasticity [1]. Sleep disorders are linked to poor health, including the progression of neurodegenerative diseases and reduced lifespan in humans. Therefore, the wide prevalence of sleep disorders in modern societies poses a major health problem [2]. Sleep is ancient in origin and most likely evolved together with the emergence of a nervous system. However, little is known about the conditions that led to the evolution of sleep and about how sleep controls basic vital functions. Studying sleep in simple animals can shed light on the essential needs fulfilled by sleep [3].

Sleep is found in all organisms that have a nervous system, ranging from jellyfish to humans [4]. Its widespread occurrence implies that sleep is important, a view supported by the finding that sleep deprivation has detrimental effects [5]. Environmental conditions can impact sleep [6, 7]. Nutrient availability often fluctuates, and all organisms have thus established strategies to sense and respond to a lack of food. Across species, starvation triggers developmental arrest and a biphasic behavioral response consisting of a first phase of increased activity and suppressed sleep, followed by decreased physical activity [8–16]. Although increased physical activity is understood as a strategy to increase foraging, less is known about the regulation and function of decreased behavioral activity following long-term starvation [17]. Modest food deprivation can exert beneficial effects, suggesting that, in order to survive food deprivation, animals adapt their physiology and activate health- and longevity-promoting pathways [16, 18].

Caenorhabditis elegans is a model animal with low ethical hurdles for harsh survival assays. It lives a boom-and-bust lifestyle with periods of rapid proliferation when food is present alternating with long periods of starvation. As an adaptation to food scarcity, *C. elegans* has evolved survival strategies, including larval developmental arrest, an alternative larval life stage called "dauer," and an increased lifespan in adults [19–21]. The study of dietary restriction and starvation and their role on lifespan in *C. elegans* has led to the identification of major conserved signaling pathways controlling development and aging. Important nutrient-sensing and lifespan-promoting systems act through AMP-activated kinase and the FoxO transcription factor [21].

Like most animals, *C. elegans* sleeps, a phenomenon that has been studied most in the developing larva and after cellular stress in the adult [22, 23]. From worms to humans, sleep is induced by conserved sleep-active neurons that depolarize at the onset of sleep. They actively induce this behavior by directly inhibiting arousal circuits through GABA and neuropeptides.

 © 2018 The Authors. Published by Elsevier Ltd.
This is an open access article under the CC BY license (http://creativecommons.org/licenses/by/4.0/).

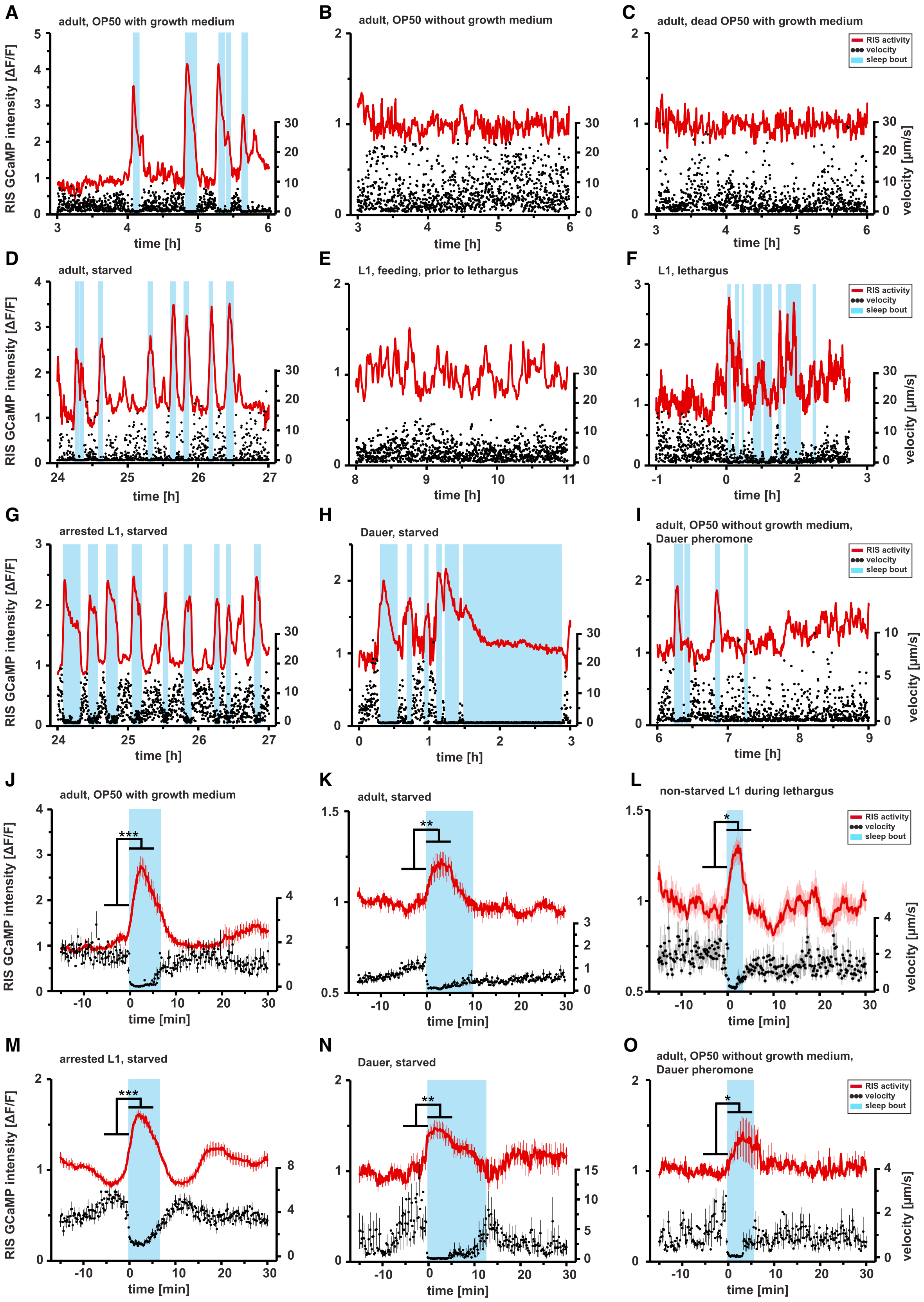

(legend on next page)

Upstream pathways control the depolarization of sleep-active neurons and thereby control the timing and amount of sleep [24, 25]. In mammals, several populations of sleep-active neurons exist, which are thought to induce sleep in a concerted action. The best-studied population of sleep-active neurons is found in the preoptic area (POA). These neurons form part of the so-called descending system that inhibits arousal during sleep. Sleep-active neurons also confer an increased sleep pressure after sleep deprivation, as their depolarization is increased at the onset of rebound sleep [26, 27]. Similarly, several populations of sleep-promoting neurons exist in *Drosophila*. A cluster of nerve cells innervating the dorsal-fan-shaped body (dFB) of the central complex presents a well-studied population of sleep-promoting neurons, whose excitability can be switched dependent on sleep need [28]. In *C. elegans*, sleep during lethargus, a developmentally controlled phase of molting during which worms synthesize a new cuticle, requires a single sleep-active sleep-inducing neuron called RIS, which expresses GABA and inhibitory RFamide peptides. Like its mammalian counterparts, this neuron induces sleep when depolarized optogenetically, shows calcium transients at sleep onset, most likely indicating depolarization, shows over-activation after sleep deprivation, and is inhibited by waking stimuli [29–31].

To find out why a simple animal sleeps, we searched for conditions in which RIS-induced sleep is essential for survival in *C. elegans*. Although worms show sleep during most stages and conditions, this behavior is most prominent during various forms of starvation and developmental arrest, where conserved nutrient-sensing lifespan regulators control it. Although sleepless adult worms have a normal lifespan under both ample food condition as well as starvation, sleeplessness impairs survival of larvae during developmental arrest. Interestingly, the role of sleep in starvation-induced arrest survival appears not to only be conservation of energy but to prevent the progression of aging phenotypes. Thus, sleep presents an adaptive anti-aging strategy to survive starvation-induced arrest. Our work provides a molecular link between sleep, longevity, starvation, and developmental arrest with high potential implications for the evolutionary origin of sleep as well as for human health.

RESULTS

Sleep Is Widespread across Stages and Conditions, with Extended Starvation Presenting a Major Sleep Trigger

To find out how sleep becomes vital in a simple animal, we quantified sleep during several life stages and conditions to find out what is the strongest trigger for this state. We focused on food availability and quality, which affects behavioral activity and quiescence [10, 11, 19, 32, 33]. We used RIS calcium imaging as a proxy for depolarization of this neuron and locomotion quiescence as an assay to identify and quantify sleep. Worms were cultured in microfluidic devices made from hydrogel [34, 35] and behavior, and calcium transients in RIS were imaged and quantified during different life stages and food conditions (Figures 1 and S1). We first looked for sleep in adult worms and tested the effects of three types of bacterial food: first, to mimic standard worm culture conditions, we fed worms with bacteria in the presence of bacterial growth medium [32] (Figure 1A). Second, we tested bacteria that were depleted of growth medium (Figure 1B). And third, we tested dead bacteria in the presence of growth medium (Figure 1C). Worms living on fed bacteria showed extended sleep bouts (Figures 2A, S2A, and S2B). A reduction of locomotion always coincided with increased RIS depolarization (Figure 1J). Worms feeding on dead or starved bacteria, however, showed virtually no sleep behavior (Figures 1B, 1C, and 2A).

Short-term (few hours) fasting results in increased arousal and foraging in *C. elegans* [15], whereas prolonged (more than 12 hr) starvation results in behavioral quiescence and developmental arrest [10, 11, 19, 20, 33, 36]. To analyze sleep during starvation and arrest, we looked at one day starved adults, L1 larvae during lethargus (when worms do not feed during cuticle remodeling), one day starved developmentally arrested L1 larvae, and three days starved dauer larvae. We observed prominent sleep bouts during starvation and arrest in all stages that were tested (Figures 1D–1H, 2B–2E, and S2C–S2J). RIS again depolarized strongly at the onset of all sleep bouts (Figures 1K–1N).

As pheromones play a strong role in anticipation of adverse life conditions and the development of the developmentally arrested dauer larva [37], we also tested the effect of pheromones on sleep in adults feeding on starved bacteria. Dauer pheromone

Figure 1. Food Conditions Control Sleep Amount in *C. elegans* across Stages

(A–I) Individual RIS calcium imaging and sleep measurements. RIS activity is shown in red and locomotion speed in black; blue shading shows sleep bouts as defined by a locomotion cessation threshold. At the onset of a sleep bout, RIS activated and locomotion ceased.

(A) An adult worm feeding on OP50 bacteria with bacterial growth medium.

(B) An adult worm feeding on OP50 without bacterial growth medium.

(C) An adult worm feeding on dead OP50 with growth medium.

(D) An adult that was starved for 24 hr.

(E) An L1 larva in the presence of food, 8 hr after hatching and prior to lethargus.

(F) An L1 larva before and during lethargus as defined as the non-feeding period (starting at 0 hr) prior to the molt in the presence of food.

(G) An arrested L1 larva that was starved for 24 hr.

(H) A 3-day-old dauer larva in the absence of food.

(I) An adult feeding on OP50 without growth medium in the presence of dauer pheromone.

(J–O) RIS activity increased significantly with locomotion cessation at the onset of sleep bouts in all conditions. The increase of $\Delta F/F$ of the calcium sensor signal was (J) 126.1% ± 20.2%, n = 24 worms, ***p < 0.001 for adults on growing OP50; (K) 32.5% ± 5.4%, n = 20 worms, **p < 0.01 for starving adults; and (L) 22.0% ± 0.9%, n = 14 worms, *p = 0.014 for developing L1 worms during lethargus.

(M–O) 58.7% ± 4.9% (M), n = 33 worms, ***p < 0.001 for arrested L1 larvae; 36.8% ± 7.9% (N), n = 13 worms, **p = 0.002 for dauer larvae; 45.7% ± 19.8% (O), n = 12 worms, *p = 0.02 for adults on stationary OP50 with dauer pheromone.

For all statistical comparisons, the paired Wilcoxon rank test was used. See also Figures S1 and S2.

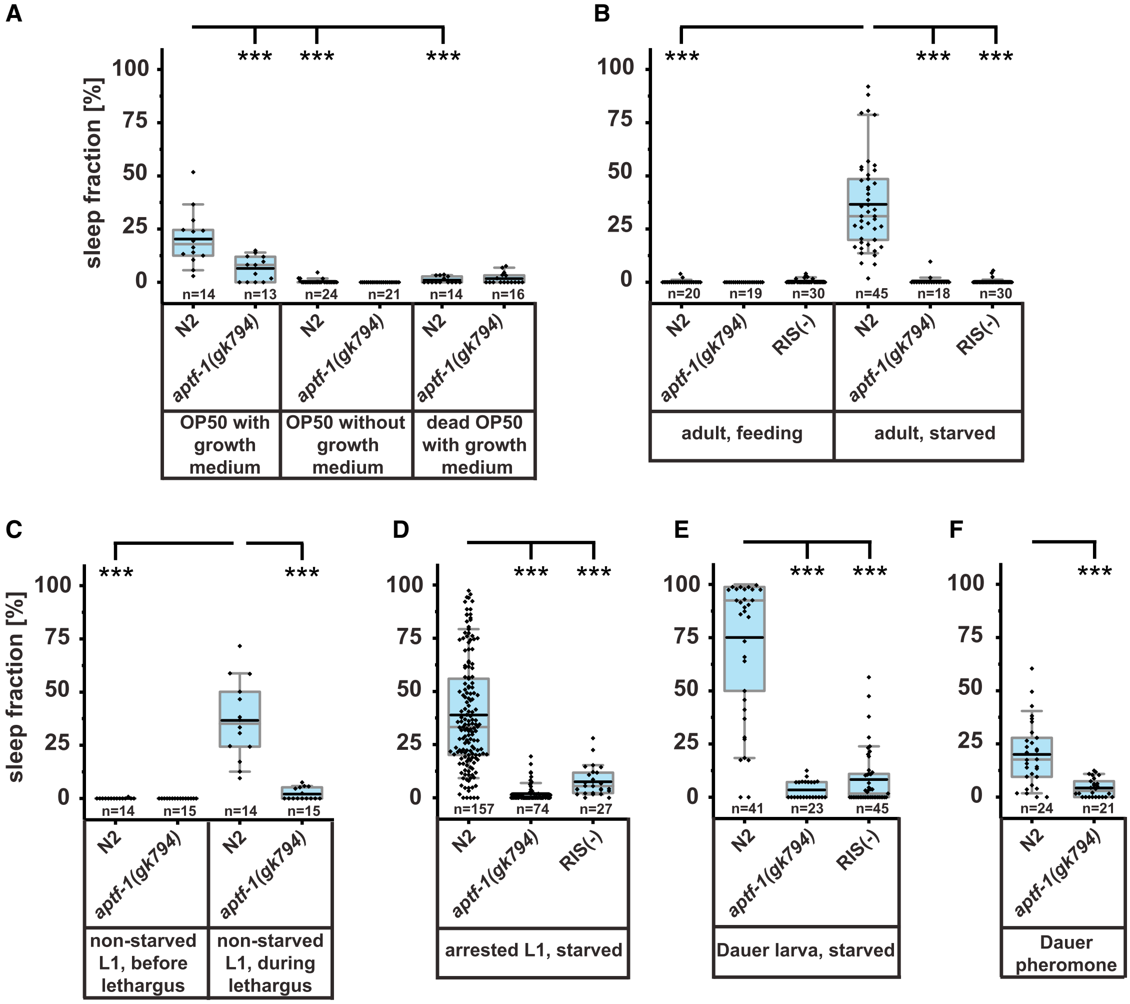

Figure 2. The Sleep-Active RIS Neuron Is Required for All Types of Physiological Sleep

Fraction of time spent asleep in wild-type, *aptf-1(gk794)*, and RIS-ablation (RIS(–)).

(A) Median sleep duration in adult worms in the presence of growing bacteria was 18% in wild-type and 8% in *aptf-1(gk794)*; ***p < 0.001. In the absence of growth medium and in the presence of dead bacteria, median sleep duration was 0% for both wild-type and *aptf-1(gk794)*.

(B) In adult worms, starvation increased sleep compared with worms fed on bacteria in the absence of growth medium. Median sleep duration in starved adults was 31% in wild-type and 0% in *aptf-1(gk794)* and RIS(–); ***p < 0.001.

(C) Fraction of time spent asleep in wild-type and *aptf-1(gk794)* in fed L1 prior to and during lethargus. Before lethargus, median sleep fraction was 0% for both wild-type and *aptf-1(–)*. During lethargus, the median sleep fraction was 35% for wild-type and 0% for *aptf-1(gk794)*; ***p < 0.001.

(D) Sleep fraction in starved L1 larvae. Wild-type larvae had a median sleep fraction of 33%, *aptf-1(gk794)* 0%, and RIS(–) 5%; ***p < 0.001.

(E) Sleep fraction in dauer larvae. Median sleep fraction in dauer larvae was 92% wild-type, 0% in *aptf-1(gk794)*, and 2% in RIS(–); ***p < 0.001.

(F) Sleep fraction in adult worms in the presence of dauer pheromone. Wild-type worms showed a median sleep fraction of 18% and *aptf-1(gk794)* of 3%; ***p < 0.001.

The numbers of assayed worms (n) are displayed below the boxplots. Statistical comparisons were made using the Mann-Whitney U test.

extract also induced sleep bouts and RIS depolarization, which is consistent with the observation that population density affects sleep (Figures 1I, 1O, 2F, S2K, and S2L) [16].

In summary, behavioral quiescence was typically accompanied by RIS activation at the onset of virtually all sleep bouts and across all stages and conditions that we tested. An increase in RIS calcium precisely correlates with reduction of behavioral activity. Thus, rather than reflecting sleep pressure building up during wakefulness prior to sleep onset [11, 38], RIS activity indicates the active induction of sleep. Food conditions appear to be a major determinant for RIS activation and sleep amounts in both larvae and adults. Not only the presence or absence of food determines sleep quantity but also food quality, with metabolizing bacteria appearing as a strong sleep trigger. Extended starvation and arrested development triggered the strongest sleeping behavior. As pheromones signal population density, sleep may already be modulated in anticipation of starvation. RIS calcium imaging combined with locomotion quantification thus presents a straightforward assay to identify sleep, which occurred during all stages and most conditions and thus is much wider spread than previously thought [22, 39].

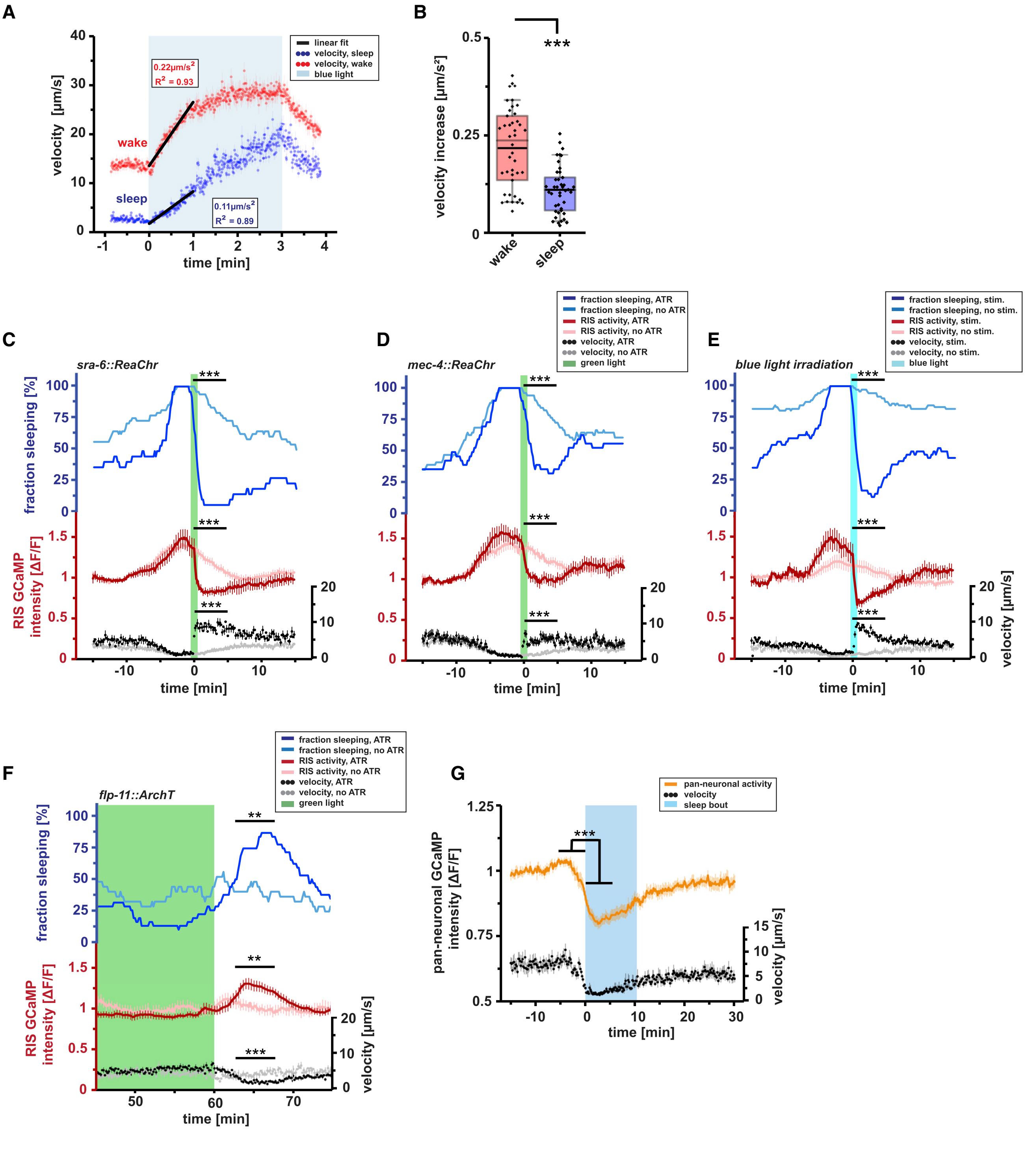

Figure 3. Behavioral Quiescence during L1 Arrest Presents a Sleep State

(A and B) To test for responsiveness to stimulation during quiescence in arrested L1 larvae (A), a blue light stimulus was given and locomotion velocity was measured. (B) Linear regression of locomotion speeds during the first minute of blue light irradiation was used to measure locomotion acceleration. Waking worms (red dotted line, red box) accelerated with 0.22 ± 0.02 $\mu m/s^2$, whereas sleeping worms (blue dotted line, blue box) accelerated only with 0.11 ± 0.01 $\mu m/s^2$ (n = 42 worms; ***$p < 0.001$; paired Wilcoxon rank test).

(C–E) To test for sleep reversibility, nociceptive ASH neurons and *mec-4* expressing mechano-sensory neurons were stimulated optogenetically with ReaChr and green light in sleeping arrested L1 larvae. In addition, arrested L1 larvae were stimulated with noxious blue light. RIS activity is shown in red (control without all trans-Retinal [ATR] in light red), speed in black (control without ATR in gray), and the fraction of sleeping animals in blue (control without ATR in light blue).

(legend continued on next page)

Across Life Stages and Physiological Conditions, Sleep Is Induced by the Sleep-Active RIS Neuron

RIS is crucially required for sleep during lethargus [29, 30]. During un-physiological conditions, such as after severe cellular stress, a second neuron, called ALA, has been suggested to induce sleep independently of RIS [39–41]. Our imaging results suggest that sleep, at least under more physiological conditions, generally involves the RIS neuron. We hence tested whether the types of sleep that we observed depend on RIS. To ablate RIS function, we used *aptf-1* mutant worms that lack neuropeptide expression in RIS, which is required for lethargus sleep induction. In addition, we genetically ablated RIS by expression of the apoptosis inducer EGL-1 from a RIS-specific promoter [30]. In RIS-deficient worms, sleep was strongly reduced or virtually abolished (median sleep fraction in fed adult worms with growth medium was reduced by 56% in *aptf-1(gk794)*; in starved adults by 100% in *aptf-1(gk794)* and RIS(−); during lethargus by 100% in *aptf-1(gk794)*; in arrested L1 larvae by 100% and 84% in *aptf-1(gk794)* and RIS(−), respectively; in dauer larvae by 100% and 98% in *aptf-1(gk794)* and RIS(−), respectively; and in adults in the presence of dauer pheromone by 83% in *aptf-1(gk794)*; Figure 2). Thus, consistent with our imaging data, RIS is required for sleep under all studied conditions. This suggests a pivotal role of RIS in sleep under physiological conditions.

Starvation-Induced Quiescence during L1 Arrest Is a Sleep State

The dependency of quiescence on RIS indicates that most types of behavioral quiescence in the worm constitute sleep rather than other types of quiescence, such as quiet wakefulness or paralysis. However, quiescence during L1 arrest has not yet been characterized as sleep using behavioral criteria [10, 11, 19, 32, 33, 41–43]. Sleep is defined and can be distinguished from other types of quiescence by a reduced response threshold, rapid reversibility, and homeostatic regulation [44]. We hence assayed whether quiescence during L1 arrest fulfills the behavioral definition of sleep.

To test for responsiveness, we used blue light to trigger an escape response [45] in arrested L1 larvae inside microfluidic compartments and measured the resulting increase in locomotion. Although waking worms responded with a strong locomotion increase, during sleep, the acceleration was reduced (Figures 3A and 3B). We next tested for reversibility of sleep by providing a strong optogenetic sensory stimulus. For this, we expressed the channelrhodopsin variant ReaChR in nociceptive ASH neurons or mechano-sensory neurons, depolarized them with green light [46], and followed locomotion and RIS polarization. Optogenetic stimulation of ASH neurons or *mec-4*-expressing neurons quickly reversed quiescence (Figures 3C, 3D, S3A, and S3B). The waking stimulus also acutely inhibited RIS activity, leading to a premature termination of the RIS depolarization transient (Figures 3C, 3D, S3A, and S3B). To provide a non-optogenetic sensory stimulus, we applied noxious blue light, which triggers an avoidance response [45, 47]. Similar to the optogenetic stimulus, blue light reversed quiescence and inhibited RIS (Figures 3E and S3C).

To test for homeostatic regulation, we deprived sleep through temporary optogenetic inhibition of RIS using the light-driven proton pump ArchT [48]. We inhibited RIS for 1 hr using green light and measured the behavioral response and the depolarization of the RIS neuron before, during, and after the inhibition. Optogenetic silencing efficiently prevented RIS depolarization and sleep induction during illumination (Figures S3D and S3E). Following the end of RIS inhibition, sleeping behavior and RIS depolarization increased compared to control levels, suggesting that quiescence is under homeostatic control (Figures 3F, S3D, and S3E).

During sleep, including *C. elegans* lethargus, the majority of neurons show dampened activity [38, 49]. To test whether global neural activity is reduced during sleep, we imaged pan-neuronal calcium activity. Global brain activity was indeed dampened during quiescence bouts (Figure 3G).

Thus, starvation-induced quiescence during larval arrest presents a sleep state, with its key behavioral and neurophysiological hallmarks. Regulatory principles typical for mammalian sleep are present also in starvation-induced sleep: the fast inhibition of RIS through a waking stimulus is similar to the flip flop switch that ensures discrete behavioral states and the increased RIS

(C) Sleeping worms responded immediately to ASH activation and the sleep bout ceased. The fraction of sleeping animals decreased by 91.6% ± 1.7% (control worms without ATR by 12.3% ± 2.9%; ***p < 0.001; two-sample t test). RIS activity (ΔF/F) dropped by 35.5% ± 4.2% (control worms without ATR showed only a drop of ΔF/F of 11.9% ± 0.3%; ***p < 0.001 two-sample t test). Locomotion velocity increased by 313.7% ± 26.0% (control worms without ATR by 65.7% ± 14.0%; ***p < 0.001; two-sample t test). $n_{(ATR)}$ = 23 worms; $n_{(without\ ATR)}$ = 47 worms.

(D) Sleeping worms immediately responded to *mec-4*-expressing mechano-sensory neuron activation, and the sleep bout ceased. The fraction of sleeping animals decreased by 52.45% ± 6.0% (control worms without ATR by 10.0% ± 2.6%; ***p < 0.001; two-sample t test). RIS activity (ΔF/F) dropped by 32.8% ± 3.2% (control worms without ATR showed only a drop of ΔF/F of 7.0% ± 2.1%; ***p < 0.001; two-sample t test). Locomotion velocity increased by 351.7% ± 44.0% (control worms without ATR by 14.6% ± 4.0%; ***p < 0.001; two-sample t test). $n_{(ATR)}$ = 29 worms; $n_{(without\ ATR)}$ = 50 worms.

(E) Sleeping worms immediately responded to noxious blue light illumination, leading to sleep bout termination. The fraction of sleeping animals decreased by 75.5% ± 3.5% (control worms without blue light stimulation by 4.1% ± 0.6%; ***p < 0.001; two-sample t test). RIS activity (ΔF/F) dropped by 43.2% ± 7.9% (control worms without blue light stimulation showed only a drop of ΔF/F of 4.3% ± 7.7%; ***p < 0.001; two-sample t test). Locomotion velocity increased by 367.9% ± 5.8% (control worms without ATR by 80.3% ± 28.2%; ***p < 0.001; two-sample t test). $n_{(stimulation)}$ = 35 worms; $n_{(without\ stimulation)}$ = 37 worms.

(F) To test for homeostasis, RIS was inhibited for 1 hr with ArchT and green light in the arrested L1 larvae. RIS activity is shown in red (control without ATR in light red), speed in black (control without ATR in gray), and the fraction of sleeping animals in blue (control without ATR in light blue). After the end of green light exposure, the fraction of sleeping worms increased by 265.6% ± 13.1% (control worms without ATR decreased by 19.3% ± 6.7%; **p = 0.007; two-sample t test). RIS activity (ΔF/F) increased by 24.3% ± 2.2% (in control worms without ATR, it increased by 5.6% ± 1.0%; **p = 0.005; two-sample t test). Locomotion velocity decreased by 63.3% ± 16.0% (control worms without ATR increased by 5.5% ± 4.0%; ***p < 0.001; two-sample t test). $n_{(ATR)}$ = 32 worms; $n_{(without\ ATR)}$ = 25 worms.

(G) To test for overall neuronal activity during a sleep bout, calcium activity in the head neurons was measured using pan-neuronal GCaMP6s. Head neuron activity is shown in orange and locomotion speed in black; blue shading shows sleep bouts as defined by a locomotion cessation threshold. At the onset of a sleep bout, head neuron activity decreased and locomotion ceased. ΔF/F was decreased to 82.3% ± 0.2% (n = 21 worms; ***p < 0.001; paired Wilcoxon rank test). Error bars in (C)–(G) indicate the SEM. See also Figure S3.

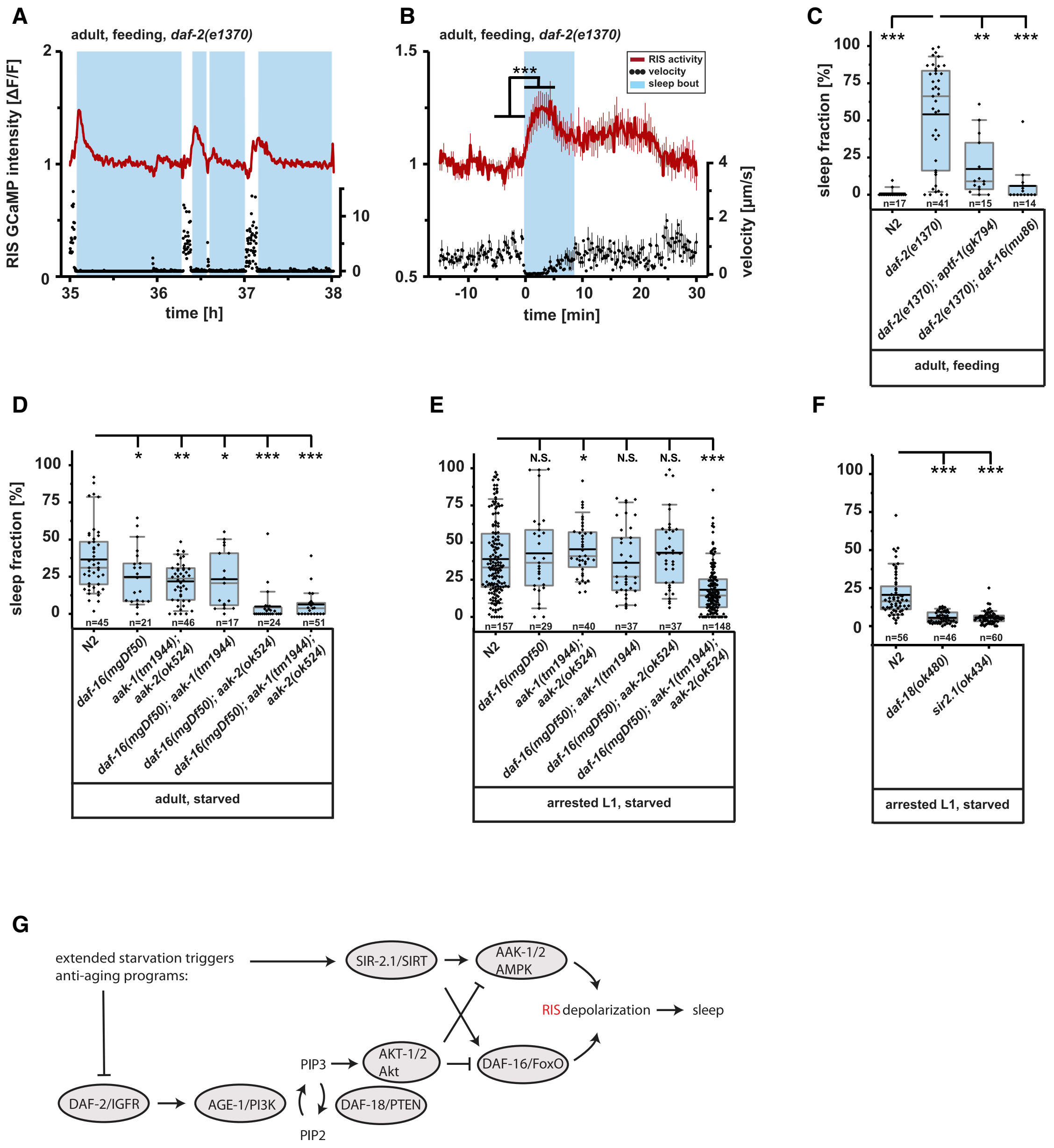

Figure 4. The Nutrient-Sensing Longevity Regulators IIS and AMP Kinase Regulate Starvation Sleep through RIS Activation

(A) RIS GCaMP3 signal intensities and corresponding velocity traces of a representative *daf-2(e1370)* mutant worm. RIS activity is shown in red and locomotion speed in black; blue shading shows sleep bouts as defined by a locomotion cessation threshold. At the onset of a sleep bout, RIS activated and locomotion ceased. Note that RIS depolarizes at sleep onset and is not depolarized during the entire quiescence bout, which is similarly observed in the dauer larva.

(B) Averaged RIS activity and velocity aligned to sleep bout onset. RIS activity (ΔF/F) increased by 17.9% ± 4.7% during sleep (n = 15 worms; ***$p < 0.001$; paired Wilcoxon rank test).

(C) DAF-2 inhibition induces sleep through RIS and DAF-16. Median sleep time was 66% in *daf-2(e1370)*; 9% in *daf-2(e1370); aptf-1(gk794)*, **$p < 0.01$; and 0% in *daf-2(e1370)*; *daf-16(mu86)*, ***$p < 0.001$.

(D) Longevity genes control starvation-induced sleep in the adult: median time spent in sleep was 31% in wild-type; 24% in *daf-16(mgDf50)* mutation, *$p < 0.05$; 24% in *aak-1(tm1944);aak-2(ok524)* double mutants, **$p < 0.01$; 21% in *daf-16(mgDf50)/aak-1(tm1944)* double mutants, *$p < 0.05$; 0% in *daf-16(mgDf50)/aak-2(ok524)* double mutants, ***$p < 0.001$; and 4% in *daf-16(mgDf50)/aak-1(tm1944)/aak-2(ok524)* triple mutants, ***$p < 0.001$.

(E) Longevity genes control starvation-induced larval sleep. Single or double mutation combinations did not reduce sleep significantly, but, compared with wild-type (median sleep fraction 33%), the *daf-16(mgDf50)/aak-1(tm1944)/aak-2(ok524)* triple mutant reduced sleep (median sleep fraction 14%); *$p < 0.05$; ***$p < 0.001$.

(F) Insulin signaling and sirtuin, which control the activity of FoxO and AMPK, control sleep. Median sleep fraction was 16% in wild-type; 4% in *daf-18(ok480)*, ***$p < 0.001$; and 5% in *sir-2.1(ok434)*, ***$p < 0.001$.

(legend continued on next page)

depolarization during rebound sleep also occurs similarly in sleep-active neurons of the vertebrate POA [26].

AMP-Activated Kinase and FoxO Act in Parallel to Induce Sleep during Starvation

Starvation is sensed by conserved pathways that trigger protective strategies. AMP-activated kinase (AAK-1/2) is an energy sensor that is activated by a high AMP/ATP ratio and that triggers the switch from anabolic to catabolic processes. Reduced activity of the insulin/insulin-like signaling (IIS) receptor (DAF-2) leads to the activation of FoxO (DAF-16), a transcription factor that expresses genes that promote stress resistance and longevity [21]. DAF-16 also plays a role in coping with the consequences of stressful sleep deprivation and modulates quiescence [11, 50–52]. Both FoxO and AMPK are required for starvation-induced developmental arrest [20, 53]. To test whether longevity pathways are responsible for the majority of starvation sleep, we measured RIS activation and sleep in mutants that are defective in AMP-activated kinase and IIS.

We first tested a loss-of-function mutation in *daf-2* in the presence of food conditions that normally do not lead to sleep. *daf-2(−)* caused sleep and RIS depolarization in the presence of starved bacteria as a food source (Figures 4A–4C, S2M, and S2N), and this effect was dependent on *daf-16* (Figure 4C). We next tested sleep in *daf-16(−)* during larval and adult starvation. *daf-16(−)* showed only a moderate reduction of sleep in adults, consistent with previous results (Figure 4D) [11]. Similarly, partially reduced sleeping behavior was found in *aak-1/aak-2* double-mutant animals during starvation (Figure 4D), prompting us to knock out both pathways simultaneously. The *aak-2/daf-16* double mutant showed a near-complete absence of sleep in the adult (Figure 4D), but not in the larva (Figure 4E). *aak-1/aak-2/daf-16* triple mutant worms showed a strong loss of sleep in the starved adult (Figure 4D) and also a strong sleep reduction in the arrested L1 larva (Figure 4E). Thus, AAK-1/2 and DAF-16 appear to act in parallel to induce sleep during extended starvation.

We next wished to link starvation-induced sleep to the genes that are upstream of AMPK and FoxO. We hence quantified sleep in mutants of genes that are known to control AMPK and FoxO. In the presence of food, IIS activates the phosphatidylinositol 3-kinase (PI3K) AGE-1 to produce PIP3, which activates Akt kinase (AKT-1/2) [21]. A major role of Akt in *C. elegans* is to inhibit FoxO [54]. In other systems, Akt also inhibits AMPK by adding an inhibitory phosphorylation on Ser485, which then inhibits the activation of AMPK by LKB1 [55], and there is evidence that Akt also inhibits AMPK in *C. elegans* [56]. Thus, both FoxO and AMPK have in common that they can be suppressed through Akt [55–57]. The tumor suppressor DAF-18/PTEN inhibits IIS signaling by hydrolyzing PIP3 produced by AGE-1/PI3K. *daf-18* deletion thus leads to constitutive activation of Akt [21]. We tested whether *daf-18* deletion leads to a suppression of starvation-induced sleep and found that sleep was strongly reduced (Figure 4F). Starvation also leads to the activation of SIRT/SIR-2.1, which can act through activation of both FoxO [57] and AMPK [58]. We thus tested sleep after deletion of *sir-2.1*. We observed again a reduction of sleep in *sir-2.1(−)* mutants (Figure 4F). These results are consistent with the known network of longevity genes and a model in which, in the presence of food, canonical IIS is wake promoting through the inhibition of AMPK and FOXO and in which, during starvation, sirtuin signaling is sleep-promoting by activating AMPK and FOXO (Figure 4G). Such dual control mechanism of two parallel pathways could account for the robustness of sleep induction during starvation. Our data thus suggest that the known network of longevity genes controls sleep induction during starvation.

To find out in which tissues AMPK and FoxO act during starvation to induce sleep, we performed rescue experiments. We used *daf-2(−)/daf-16(−)* worms and expressed *daf-16* specifically in either neurons, muscle, or intestine and quantified sleep in adults in the presence of food [59, 60]. Sleep was reinstated partially but significantly by expression in muscle and inconsistently by expression in intestine, but not by expression in neurons (Figure S4A). The role of *daf-16* in muscle during starvation is consistent with previous data suggesting a role of *daf-16* in muscle to control increased sleep after forced locomotion during lethargus and supports a conserved role of muscle in signaling sleep need [51, 61]. During physical exercise, musculature is the major consumer of energy, and this tissue may thus be well suited to signal energy shortage during both forced movement and starvation [62]. The weak rescue from non-neuronal tissues together with the lack of neuronal rescue suggests that *daf-16* acts in signaling centers outside of RIS.

To identify the tissues in which AMPK acts, we expressed *aak-2* in an *aak-2(−)/daf-16(−)* background. We quantified sleep in adult worms in the absence of food and looked for rescue in neurons, muscle, intestine, hypodermis, and the excretory cell [15, 56, 63]. AMPK rescue effects were partial but significant in all tissues that were tested. Because AMPK could be rescued from all tissues, including neurons, we specifically tested whether AMPK also can act in RIS and found that it partially could (Figures S4B and S4C). Thus, AMPK appears to be able to induce sleep by acting in many tissues, including in RIS. Consistent with previous reports, which found that AMPK can act across various tissues, this may indicate that many or perhaps even all tissues that experience energy stress can signal to RIS for sleep induction [15, 56, 63].

Sleep Is Required to Survive Larval Starvation-Induced Arrest

The strong induction of sleep during starvation and arrest through aging pathways suggested that sleep might play a role in surviving food deprivation and development cessation [18, 64]. Thus, we tested adult lifespan along with survival and recovery from L1 arrest in the presence or absence of food in sleepless mutants. We first measured the lifespan of adult worms in the presence of food and during starvation. In both conditions, there was no consistent and significant difference

(G) Hypothetical working model that integrates literature data with our observations. The numbers of assayed worms (n) are displayed below the boxplots. Sleep duration comparisons with N2 were made using the Mann-Whitney U test and were confirmed with Benjamini-Hochberg procedure for multiple comparisons. Error bars indicate the SEM.
See also Figure S4 for tissue-specific rescue experiments.

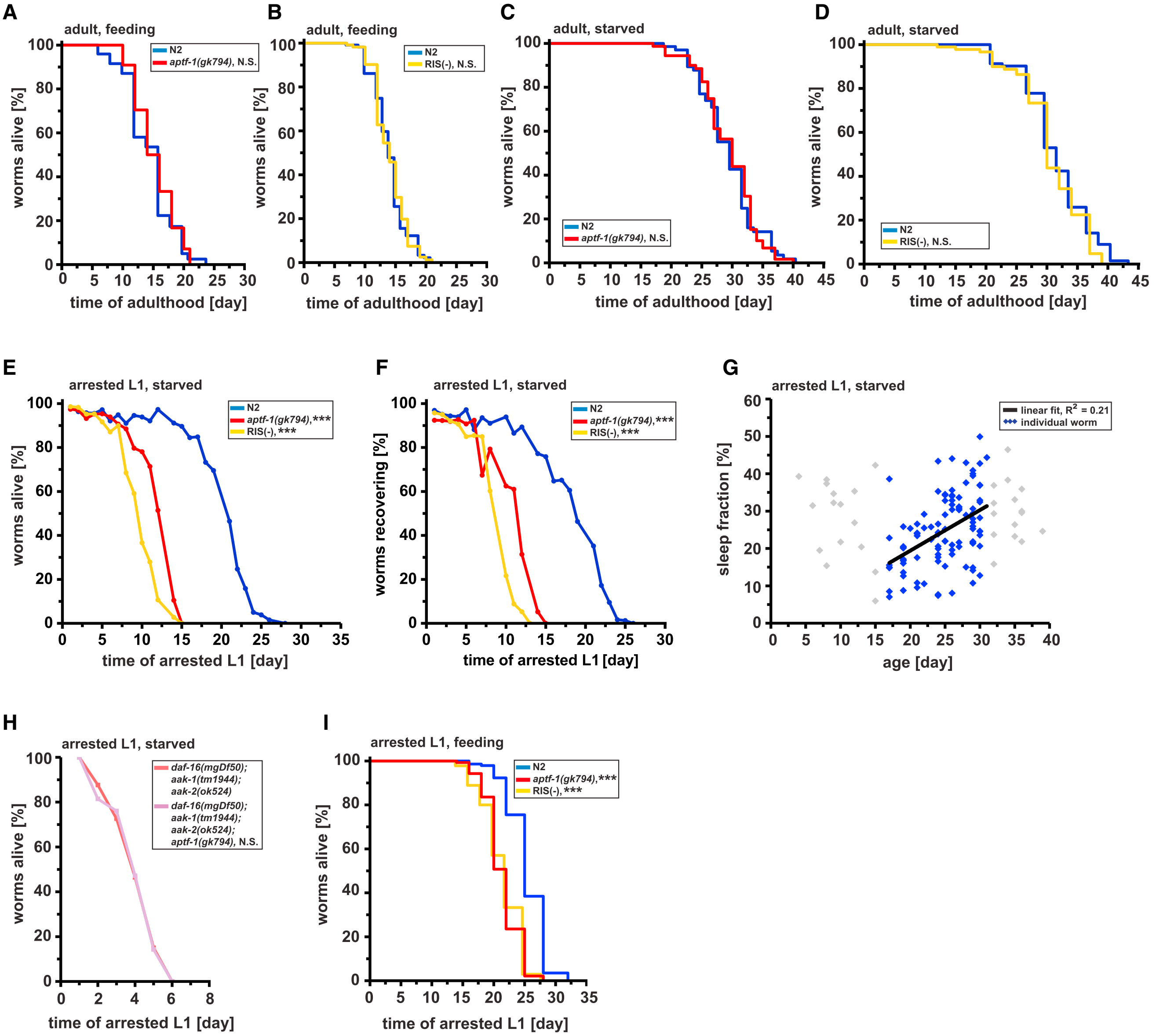

Figure 5. Sleep Is Required for Surviving Larval Starvation-Induced Arrest

Shown are representative experiments; additional replicates can be found in Figure S5.

(A) Adult lifespans of feeding wild-type (blue) and *aptf-1(gk794)* (red) worms were not significantly different (mean lifespan, N2: 14.6 days, n = 44 worms; *aptf-1(gk794)*: 15.3 days, n = 43 worms; p = 0.71; log rank test).

(B) Lifespan of feeding wild-type (blue) and RIS(–) (yellow) adult worms shows no significant difference (mean lifespan, N2: 14.2 days, n = 94 worms; RIS(–): 14.1 days, n = 86 worms; p = 0.71; log rank test).

(C and D) No significant difference of lifespan was detected in (C) starved adult *aptf-1(gk794)* compared to starved N2 (mean lifespan, N2: 29.8 days, n = 60 worms; *aptf-1(gk794)*: 29.5 days, n = 63 worms; p = 0.65; log rank test) and (D) also not in starved RIS(–) (mean lifespan N2: 32.0 days, n = 85 worms; RIS(–): 30.6 days, n = 84 worms; p = 0.06; log rank test).

(E) L1-arrested *aptf-1(gk794)* mutants and RIS ablated worms showed substantially reduced survival in the absence of food (time at which 50% of worms were still alive, N2: 20.7 days, n > 60 worms for each time point; *aptf-1(gk794)*: 12.1 days, n > 60 worms; ***p < 0.001; RIS(–): 9.4 days, n > 60 worms; ***p < 0.001; significance shown from day 8; Fischer's exact test).

(F) Arrested L1 *aptf-1(gk794)* and RIS-ablated worms showed a significant decline in the ability to re-enter development when fed (time at which 50% of worms were still able to recover, N2: 18.7 days, n > 60 worms; *aptf-1(gk794)*: 11.4 days, n > 60 worms; ***p < 0.001; RIS(–): 8.5 days, n > 60 worms; ***p < 0.001; significance shown from day 8 for RIS(–) and day 10 for *aptf-1* mutant; Fischer's exact test).

(G) Sleep amount predicts starvation survival. Arrested starved L1 larvae were cultured in microchambers, and their sleep and survival were quantified. Data from individual worms within the range used for linear fitting (black line) are plotted as blue diamonds (data outside the range of the SD of the mean, including early-dying immobile individuals, were excluded; gray); $R^2 = 0.21$.

(legend continued on next page)

in lifespan between wild-type, *aptf-1(−)*, and RIS-ablated worms (Figures 5A–5D and S5A–S5D). We then tested L1 arrest survival and recoverability. For this, we measured both the time that L1 larvae stayed alive in the absence of food as well as the time until larvae were still able to recover from the arrest and to resume their development when fed [65]. Both survival time and the ability to recover were reduced consistently and significantly by about half in sleepless mutants (Figures 5E. 5F, and S5E–S5H). Thus, although there does not seem to be a strict requirement of sleep for survival in adult *C. elegans*, it becomes essential in the arrested larva.

To test whether sleep is a predictor of survival, we quantified quiescence for individual starved L1 larvae inside microfluidic devices and also measured their survival span. A correlation was seen between the amount of sleep during the first 2–4 days of starvation and survival (an increase of 1% sleeping time corresponded to an increase in survival extension by roughly 5%; Figure 5G), suggesting that sleep is a predictor for surviving starvation.

If sleep is part of the protective program induced by AAK-1/2 and IIS, then ablating sleep in AAK-1/2 and IIS mutants should not further decrease survival during larval starvation. To test this idea, we looked at survival of *aptf-1(−)* mutant worms in a FoxO/AAK-1/2 triple mutant background. There was no difference in survival due to sleep loss when we compared the *daf-16(mgDf50)/aak-1(tm1944)/aak-2(ok524)* triple mutant with *daf-16(mgDf50)/aak-1(tm1944)/aak-2(ok524)/aptf-1(gk794)* quadruple mutant (Figures 5H and S5I). This is consistent with the view that sleep and AAK/FoxO act in the same pathway.

If the role of sleep were to conserve energy or nutrients, then feeding would suppress the requirement of sleep for survival. To test this idea, we arrested L1 larvae using 5-fluoro-2′-deoxyuridine (FUdR), kept them in the presence of *ad libitum E. coli* as food source, and measured survival. The arrested larvae consumed the bacteria (Figures S6A and S6B) and showed sleeping behavior (Figure S6C). However, sleepless worms still had a significantly shorter lifetime than the sleeping control (Figures 5I and S5J). This suggests that, although resource preservation is important, sleep acts beyond energy or nutrient conservation to achieve survival.

Sleep Counteracts the Progression of Aging Phenotypes during Starvation-Induced Arrest

Recent work demonstrated that L1 arrested larvae show a progression of aging phenotypes leading to death. Thus, the cause of death of arrested larvae is similar to the one in the adult [65]. We wondered whether sleep is required for survival by slowing down the progression of aging phenotypes during starvation. To test this idea, we looked at established markers of aging, such as the deterioration of body wall muscle fibers, mitochondrial fragmentation, and protein aggregation [65]. We crossed *aptf-1(−)* into transgenic strains in which either muscle myosin, muscle mitochondria, or an aggregation-prone polyglutamine-containing protein, poly-Q35::YFP, were fluorescently labeled [65–69]. L1 larvae were starved, and the progression of aging was monitored by fluorescence microscopy until the animals had died. The morphology of the aging markers looked normal in freshly starved L1 *aptf-1(−)*. However, as time passed, aging markers progressed more quickly in the sleepless mutant compared with the wild-type. Muscle fibers degraded more quickly, mitochondria underwent fission earlier, and protein aggregation occurred faster. Sleepless worms reached the same maximal level of aging marker progression approximately ten days earlier compared with the wild-type (Figure 6). Together, these results suggest that, rather than merely saving energy, sleep is required for survival of starvation by counteracting the progression of aging processes.

DISCUSSION

To understand why sleep is important for *C. elegans*, we monitored behavioral activity and RIS calcium transients to survey the prevalence of sleep across life stages and physiological conditions. We tested for the effects of food quality and availability, as these define the major physiological conditions in the life of *C. elegans*, which proliferates in ephemeral microbe-rich habitats and is able to survive long periods of starvation [19]. Sleep occurred during most stages and conditions that were analyzed. RIS depolarized at the onset of sleep bouts and typically showed activity until the end of the sleep bout, inversely mirroring the pattern of behavioral activity. This pattern of RIS depolarization is consistent with the view that RIS is the inducer of sleep, and the maintenance of high RIS activity throughout most of the sleep bout suggests that sleep is under constant control of RIS. During longer sleep bouts, which were, for example, observed in dauer larvae and *daf-2(−)*, calcium transients still correlated with sleep onset but decreased already before the end of the bout. This may speculatively be caused by an additional mechanism that may dampen wakefulness independently of RIS, thus potentially explaining a reduced requirement for RIS activity for maintaining sleep. Consistent with this speculation, spontaneous motion is reduced in *daf-2(−)* and dauer larvae [70]. *daf-2(−)* mutants showed a residual fraction of immobility that could not be suppressed by *aptf-1(−).* Similarly, a small fraction of residual quiescence was observed in the absence of functional RIS when worms were fed with bacteria in the presence of growth medium. Different scenarios could explain this residual quiescence. First, occasionally, slow locomotion may be falsely scored as quiescence by the analysis algorithm. Second, quiescence may exist that is not sleep or is sleep but is not induced by RIS. Severe cellular stress can also induce behavioral quiescence independently of RIS, involving activation of the ALA neuron [39, 41]. Thus, hypothetically, some of the conditions that we

(H) Starved arrested L1 *aak-1(tm1944)/aak-2(ok524)/daf-16(mgDf50)/aptf-1(gk794)* quadruple mutants showed no reduced survival compared to *aak-1(tm1944)/aak-2(ok524)/daf-16(mgDf50)* triple mutants (time at which 50% of worms were still alive, *aak-1(tm1944)/aak-2(ok524)/daf-16(mgDf50):* 3.9 days, n > 60 worms; *aak-1(tm1944)/aak-2(ok524)/daf-16(mgDf50)/aptf-1(gk794):* 3.9 days, n > 60 worms; p > 0.05 for all days; Fischer's exact test).

(I) FUdR-arrested L1s in the presence of food have a decreased survival. Arrested L1 survival is significantly decreased in *aptf-1(gk794)* and in RIS(−) worms (mean survival, N2: 25.7 days, n = 143 worms; *aptf-1(gk794)*: 21.9 days, n = 140 worms; ***p < 0.001; RIS(−): 22.1 days, n = 135 worms; ***p < 0.001; log rank test). See also Figures S5 and S6 and Tables S1, S2, S3, S4, and S5 for additional replicates and details on food consumption.

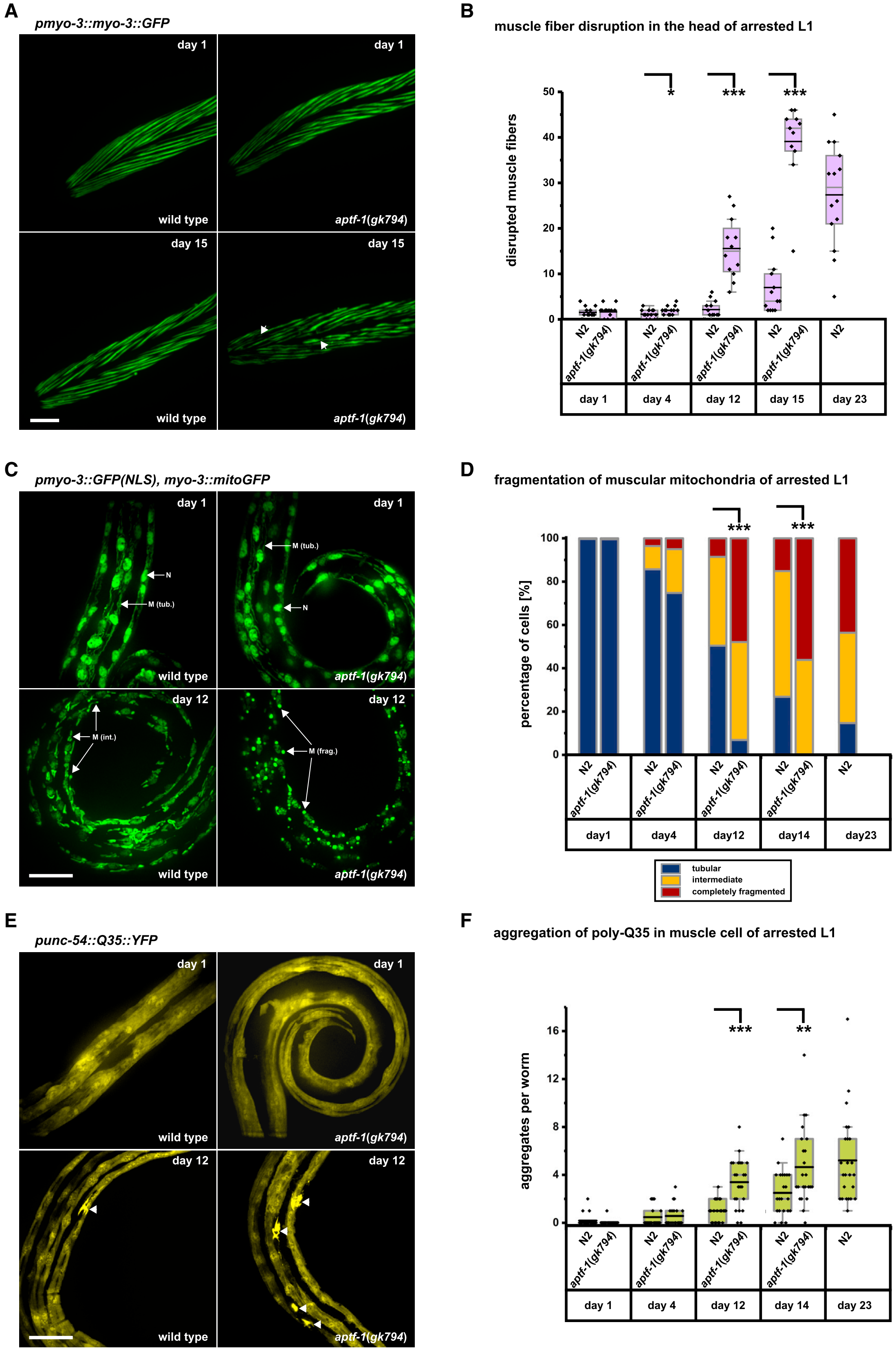

(legend on next page)

investigated may have also triggered a stress response and RIS-independent quiescence. Third, in some instances, the genetic RIS ablation might not have been fully penetrant due to transgene silencing or mutant suppression. Because, overall, only a minority of quiescent counts did not depend on RIS, we did not further investigate their cause. In summary, it emerges that, under physiological conditions, the majority of behavioral quiescence in *C. elegans* presents sleep caused by RIS.

In *C. elegans*, sleep amount strongly depends on food quality and availability, with starvation, developmental arrest, and diapause presenting the strongest sleep triggers. We hence focused our analysis on the role of sleep during starvation conditions. Starvation and aging are intimately linked: seminal work from *C. elegans* has led to the identification of genes controlling aging, including FoxO and AMP kinase, and these healthy aging genes are also required for surviving starvation [20, 21]. Genetic analysis in mice and humans showed that these genes are conserved regulators of aging [21]. Here, building on previous work on aging and sleep in *C. elegans* [11, 36, 51, 52], we linked the network of known longevity genes to sleep induction. This longevity network consists of IIS and sirtuin signaling, which control FoxO and AMPK, which activate the RIS neuron and thus induce sleep. FoxO and AMPK can act in tissues outside of RIS (even though AMPK can also act within RIS), suggesting that multiple tissues can signal starvation to induce sleep.

All organisms are threatened with the possibility of experienced famine in their life. Thus, strategies to survive the depletion of food are highly adaptive and underlying mechanisms are deeply conserved. Across species, animals show a stereotyped biphasic behavioral response to starvation. First, arousal is increased and sleep is reduced [12–16]. This phase is then followed by a phase of reduced physical activity [8–11]. Extreme conditions of food deprivation can lead to a quiescence state known as torpor, which is thought to preserve energy by shutting down physical and metabolic activity. Torpor differs from sleep in that the behavioral quiescence is not readily reversible. By contrast, sleep has been proposed to not only conserve energy but also to allocate resources [17, 71].

Does sleep serve different or similar functions during different conditions? What are the identities of these functions? And how are they carried out? Sleep has been proposed to serve diverse roles, ranging from energy conservation and allocation [6, 71] and restoration and regulation of key cellular and metabolic processes [72, 73] to higher brain functions [74, 75]. Viability assays revealed an important and specific role of sleep in surviving starvation-induced developmental arrest. Although sleep is vital for surviving starvation in larvae, we did not find any evidence that it is essential for lifespan extension in adults. Consistent with this finding, juvenile stages typically sleep more and are more susceptible to sleep deprivation also in other species [23, 76, 77]. A major biological difference between starvation in adult and larval worms is that adult worms have already completed development, with all somatic cells being post-mitotic, whereas the larva has to arrest their development, which includes the inhibition of cell division [78]. Thus, the survival experiments suggest that the vulnerability of larvae to sleep loss relates to the arrested development. Because adult worms also spent substantial time asleep, this suggests that sleep may also serve additional functions, which may not be essential for survival under ideal laboratory conditions [50, 51]. How does sleep prevent death during starvation-induced arrest? The progression of aging phenotypes, but not residual energy reserves, has been shown to predict the ability of larvae to survive and recover from starvation-induced arrest, suggesting that arrested worms do not die because they run out of energy but because of cellular demise that is mechanistically similar to aging [65]. Consistent with this finding, we observed that sleep slows down the aging phenotype progression during starvation-induced arrest. Thus, sleep appears to be required for starvation survival, not only because it saves energy from being burned for motility but rather because it counteracts aging progression. Speculatively, sleep might allocate resources away from behavioral activity toward cell-protecting pathways that are similar to anti-aging processes.

Sleep is ancient and most likely evolved to serve basic, essential needs, such as recuperation from stress and illness [41, 79]. Here, we show that sleep during starvation-induced developmental arrest presents an anti-aging program required for survival. This function could have been an important ancient reason for a sleep response, which was co-opted later during evolution to also serve higher brain functions. Most likely, the molecular link between starvation, development, aging, and sleep also plays a role in human sleep. Figuring out how sleep protects against cellular demise will be instructive to understand its revitalizing functions.

Figure 6. Sleep Counteracts the Progression of Aging Phenotypes during L1 Arrest

(A and B) Body-wall muscle myosin deteriorates faster in *aptf-1(−)*. (A) Shown is the head region of transgenic worms expressing GFP-tagged myosin (representative examples). After two days of starvation, muscle structure appears intact in both wild-type and *aptf-1(gk794)*. After fifteen days of starvation, muscle myosin deterioration has progressed much more in *aptf-1(gk794)* compared to wild-type. Arrowheads point to gaps in muscle structure. (B) Quantification of muscle deterioration. 4 days: n > 10, N2: 1.1 ± 0.3; *aptf-1(gk794)*: 2 ± 0.3, *$p < 0.05$; 12 days: n > 10, N2: 2.2 ± 0.5; *aptf-1(gk794)*: 15.6 ± 1.9, ***$p < 0.001$; 15 days: n > 10, N2: 7 ± 1.7; *aptf-1(gk794)*: 39.1 ± 2.7, ***$p < 0.001$, two-sample t test.

(C and D) Mitochondrial fragmentation is increased in *aptf-1(−)*. (C) Mitochondrial morphology appears normal in one day starved larvae, but the initially tubular mitochondria fragmented much faster in *aptf-1(−)* compared with the wild-type. Mitochondria in body wall muscles were labeled using GFP and were classified as tubular (M [tub.]), intermediately fragmented (M [int.]), or completely fragmented (M. [frag.]). Shown are the mid-body regions of worms, and arrows point to example mitochondrial structures or nucleus (N). (D) For the quantification of mitochondrial deterioration, the percentage of muscle cells displaying each level of fragmentation was determined (12 days—M (frag.): n > 20, N2: 8.5% ± 3.3%; *aptf-1(gk794)*: 47.9% ± 5.9%, ***$p < 0.001$; 14 days—M (frag.): n > 20, N2: 15% ± 3.4%; *aptf-1(gk794)*: 56.1% ± 6.1%, ***$p < 0.001$, two-sample t test).

(E and F) Protein aggregation is increased in *aptf-1(−)*. (E) Poly-Q35 was expressed in muscle, and the number of aggregates formed was scored. In one day starved L1, there were almost no detectable aggregates in both wild-type and *aptf-1(gk794)*. Shown are the mid-body regions of worms with arrowheads pointing at aggregates. (F) The number of aggregates increased much faster in *aptf-1(gk794)* compared with wild-type (12 days: n > 20, N2: 1 ± 0.2; *aptf-1(gk794)*: 3.4 ± 0.4, ***$p < 0.001$; 14 days: n > 20, N2: 2.5 ± 0.4; *aptf-1(gk794)*: 4.7 ± 0.7, **$p < 0.01$, two-sample t test).

STAR★METHODS

Detailed methods are provided in the online version of this paper and include the following:

- KEY RESOURCES TABLE
- CONTACT FOR REAGENT AND RESOURCE SHARING
- EXPERIMENTAL MODEL AND SUBJECT DETAILS
  - Worm maintenance and strains
- METHOD DETAILS
  - Molecular biology and transgenic strain generation
  - Dauer pheromone extraction and dauer larvae generation
  - Long-term imaging
  - Optogenetics and light stimulation
  - Image Analysis
  - Lifespan and survival assays
  - Quantification of bacteria consumption
  - Quantification of aging markers
- QUANTIFICATION AND STATISTICAL ANALYSIS

SUPPLEMENTAL INFORMATION

Supplemental Information includes six figures and seven tables and can be found with this article online at https://doi.org/10.1016/j.cub.2018.10.009.

ACKNOWLEDGMENTS

We thank Sabine König for extracting dauer pheromone; Juliane Schwarz for creating the *pmec-4::ReaChr* and *psra-6::ReaChr* strains; Michal Turek for creating the *pflp-11::GCaMP* and *pflp-11::ArchT* strains; Jan Konietzka for providing the agarose microchamber with N2-arrested L1 worms; Richard Roy, Shaolin Li, and Masamitsu Fukuyama for providing rescue strains; and Alexander Silbersdorff (Centre for Statistics of the University of Göttingen) for providing statistical advice. Some strains were provided by the CGC, which is funded by NIH Office of Research Infrastructure Programs (P40 OD010440). This work was supported by an Excellence Stipend of the Göttingen Graduate School for Neurosciences, Biophysics, and Molecular Biosciences to F.M., the Max Planck Society (Max Planck Research Group) to H.B., a European Research Council Starting Grant under the European Union's Horizon 2020 research and innovation program (ID: 637860; SLEEPCONTROL) to H.B., and an Exploration Grant of the Boehringer Ingelheim Stiftung Germany (Effects of sleep on ageing using *C. elegans* as a model system) to H.B. Additional data can be found in the Supplemental Information.

AUTHOR CONTRIBUTIONS

Y.W., F.M., and J.P. designed, performed, and analyzed experiments. H.B. conceived the general outline of the project, supervised the work, and wrote the paper.

DECLARATION OF INTERESTS

The authors declare no competing interests.

Received: February 20, 2018
Revised: September 11, 2018
Accepted: October 2, 2018
Published: November 8, 2018

REFERENCES

1. Cirelli, C., and Tononi, G. (2008). Is sleep essential? PLoS Biol. *6*, e216.
2. Colten, H.R., and Altevogt, B.M. (2006). Functional and economic impact of sleep loss and sleep-related disorders. In Sleep Disorders and Sleep Deprivation: An Unmet Public Health Problem, H.R. Colten, and B.M. Altevogt, eds. (National Academies Press), pp. 137–172.
3. Joiner, W.J. (2016). Unraveling the evolutionary determinants of sleep. Curr. Biol. *26*, R1073–R1087.
4. Nath, R.D., Bedbrook, C.N., Abrams, M.J., Basinger, T., Bois, J.S., Prober, D.A., Sternberg, P.W., Gradinaru, V., and Goentoro, L. (2017). The jellyfish Cassiopea exhibits a sleep-like state. Curr. Biol. *27*, 2984–2990.e3.
5. Rechtschaffen, A., and Bergmann, B.M. (1995). Sleep deprivation in the rat by the disk-over-water method. Behav. Brain Res. *69*, 55–63.
6. Siegel, J.M. (2009). Sleep viewed as a state of adaptive inactivity. Nat. Rev. Neurosci. *10*, 747–753.
7. Lesku, J.A., Rattenborg, N.C., Valcu, M., Vyssotski, A.L., Kuhn, S., Kuemmeth, F., Heidrich, W., and Kempenaers, B. (2012). Adaptive sleep loss in polygynous pectoral sandpipers. Science *337*, 1654–1658.
8. Novak, C.M., Jiang, X., Wang, C., Teske, J.A., Kotz, C.M., and Levine, J.A. (2005). Caloric restriction and physical activity in zebrafish (Danio rerio). Neurosci. Lett. *383*, 99–104.
9. Severinsen, T., and Munch, I.C. (1999). Body core temperature during food restriction in rats. Acta Physiol. Scand. *165*, 299–305.
10. McCloskey, R.J., Fouad, A.D., Churgin, M.A., and Fang-Yen, C. (2017). Food responsiveness regulates episodic behavioral states in *Caenorhabditis elegans*. J. Neurophysiol. *117*, 1911–1934.
11. Skora, S., Mende, F., and Zimmer, M. (2018). Energy scarcity promotes a brain-wide sleep state modulated by insulin signaling in C. elegans. Cell Rep. *22*, 953–966.
12. Danguir, J., and Nicolaidis, S. (1979). Dependence of sleep on nutrients' availability. Physiol. Behav. *22*, 735–740.
13. Borbély, A.A. (1977). Sleep in the rat during food deprivation and subsequent restitution of food. Brain Res. *124*, 457–471.
14. Wang, T., Hung, C.C., and Randall, D.J. (2006). The comparative physiology of food deprivation: from feast to famine. Annu. Rev. Physiol. *68*, 223–251.
15. Ahmadi, M., and Roy, R. (2016). AMPK acts as a molecular trigger to coordinate glutamatergic signals and adaptive behaviours during acute starvation. eLife *5*, e16349.
16. Goetting, D.L., Soto, R., and Van Buskirk, C. (2018). Food-dependent plasticity in *Caenorhabditis elegans* stress-induced sleep is mediated by TOR-FOXA and TGF-β signaling. Genetics *209*, 1183–1195.
17. Kilduff, T.S., Krilowicz, B., Milsom, W.K., Trachsel, L., and Wang, L.C. (1993). Sleep and mammalian hibernation: homologous adaptations and homologous processes? Sleep *16*, 372–386.
18. Kapahi, P., Kaeberlein, M., and Hansen, M. (2017). Dietary restriction and lifespan: lessons from invertebrate models. Ageing Res. Rev. *39*, 3–14.
19. Frézal, L., and Félix, M.A. (2015). C. elegans outside the Petri dish. eLife *4*, e05849.
20. Baugh, L.R. (2013). To grow or not to grow: nutritional control of development during Caenorhabditis elegans L1 arrest. Genetics *194*, 539–555.
21. Kenyon, C.J. (2010). The genetics of ageing. Nature *464*, 504–512.
22. Trojanowski, N.F., and Raizen, D.M. (2016). Call it worm sleep. Trends Neurosci. *39*, 54–62.
23. Kayser, M.S., and Biron, D. (2016). Sleep and development in genetically tractable model organisms. Genetics *203*, 21–33.
24. Saper, C.B., Scammell, T.E., and Lu, J. (2005). Hypothalamic regulation of sleep and circadian rhythms. Nature *437*, 1257–1263.
25. Bringmann, H. (2018). Sleep-active neurons: conserved motors of sleep. Genetics *208*, 1279–1289.
26. Saper, C.B., Fuller, P.M., Pedersen, N.P., Lu, J., and Scammell, T.E. (2010). Sleep state switching. Neuron *68*, 1023–1042.
27. Alam, M.A., Kumar, S., McGinty, D., Alam, M.N., and Szymusiak, R. (2014). Neuronal activity in the preoptic hypothalamus during sleep deprivation and recovery sleep. J. Neurophysiol. *111*, 287–299.

28. Pimentel, D., Donlea, J.M., Talbot, C.B., Song, S.M., Thurston, A.J.F., and Miesenböck, G. (2016). Operation of a homeostatic sleep switch. Nature *536*, 333–337.

29. Turek, M., Lewandrowski, I., and Bringmann, H. (2013). An AP2 transcription factor is required for a sleep-active neuron to induce sleep-like quiescence in C. elegans. Curr. Biol. *23*, 2215–2223.

30. Turek, M., Besseling, J., Spies, J.P., König, S., and Bringmann, H. (2016). Sleep-active neuron specification and sleep induction require FLP-11 neuropeptides to systemically induce sleep. eLife *5*, e12499.

31. Spies, J., and Bringmann, H. (2018). Automated detection and manipulation of sleep in C. elegans reveals depolarization of a sleep-active neuron during mechanical stimulation-induced sleep deprivation. Sci. Rep. *8*, 9732.

32. You, Y.J., Kim, J., Raizen, D.M., and Avery, L. (2008). Insulin, cGMP, and TGF-beta signals regulate food intake and quiescence in C. elegans: a model for satiety. Cell Metab. *7*, 249–257.

33. Cassada, R.C., and Russell, R.L. (1975). The dauerlarva, a post-embryonic developmental variant of the nematode Caenorhabditis elegans. Dev. Biol. *46*, 326–342.

34. Bringmann, H. (2011). Agarose hydrogel microcompartments for imaging sleep- and wake-like behavior and nervous system development in Caenorhabditis elegans larvae. J. Neurosci. Methods *201*, 78–88.

35. Turek, M., Besseling, J., and Bringmann, H. (2015). Agarose microchambers for long-term calcium imaging of Caenorhabditis elegans. J. Vis. Exp. e52742.

36. Gaglia, M.M., and Kenyon, C. (2009). Stimulation of movement in a quiescent, hibernation-like form of Caenorhabditis elegans by dopamine signaling. J. Neurosci. *29*, 7302–7314.

37. Hu, P.J. (2007). Dauer. WormBook, 1–19.

38. Nichols, A.L.A., Eichler, T., Latham, R., and Zimmer, M. (2017). A global brain state underlies *C. elegans* sleep behavior. Science *356*, eaam6851.

39. Trojanowski, N.F., Nelson, M.D., Flavell, S.W., Fang-Yen, C., and Raizen, D.M. (2015). Distinct mechanisms underlie quiescence during two Caenorhabditis elegans sleep-like states. J. Neurosci. *35*, 14571–14584.

40. Van Buskirk, C., and Sternberg, P.W. (2007). Epidermal growth factor signaling induces behavioral quiescence in Caenorhabditis elegans. Nat. Neurosci. *10*, 1300–1307.

41. Hill, A.J., Mansfield, R., Lopez, J.M., Raizen, D.M., and Van Buskirk, C. (2014). Cellular stress induces a protective sleep-like state in C. elegans. Curr. Biol. *24*, 2399–2405.

42. Raizen, D.M., Zimmerman, J.E., Maycock, M.H., Ta, U.D., You, Y.J., Sundaram, M.V., and Pack, A.I. (2008). Lethargus is a Caenorhabditis elegans sleep-like state. Nature *451*, 569–572.

43. Iwanir, S., Tramm, N., Nagy, S., Wright, C., Ish, D., and Biron, D. (2013). The microarchitecture of C. elegans behavior during lethargus: homeostatic bout dynamics, a typical body posture, and regulation by a central neuron. Sleep *36*, 385–395.

44. Campbell, S.S., and Tobler, I. (1984). Animal sleep: a review of sleep duration across phylogeny. Neurosci. Biobehav. Rev. *8*, 269–300.

45. Edwards, S.L., Charlie, N.K., Milfort, M.C., Brown, B.S., Gravlin, C.N., Knecht, J.E., and Miller, K.G. (2008). A novel molecular solution for ultraviolet light detection in Caenorhabditis elegans. PLoS Biol. *6*, e198.

46. Lin, J.Y., Knutsen, P.M., Muller, A., Kleinfeld, D., and Tsien, R.Y. (2013). ReaChR: a red-shifted variant of channelrhodopsin enables deep transcranial optogenetic excitation. Nat. Neurosci. *16*, 1499–1508.

47. Ward, A., Liu, J., Feng, Z., and Xu, X.Z. (2008). Light-sensitive neurons and channels mediate phototaxis in C. elegans. Nat. Neurosci. *11*, 916–922.

48. Han, X., Chow, B.Y., Zhou, H., Klapoetke, N.C., Chuong, A., Rajimehr, R., Yang, A., Baratta, M.V., Winkle, J., Desimone, R., and Boyden, E.S. (2011). A high-light sensitivity optical neural silencer: development and application to optogenetic control of non-human primate cortex. Front. Syst. Neurosci. *5*, 18.

49. Schwarz, J., Lewandrowski, I., and Bringmann, H. (2011). Reduced activity of a sensory neuron during a sleep-like state in Caenorhabditis elegans. Curr. Biol. *21*, R983–R984.

50. Sanders, J., Scholz, M., Merutka, I., and Biron, D. (2017). Distinct unfolded protein responses mitigate or mediate effects of nonlethal deprivation of C. elegans sleep in different tissues. BMC Biol. *15*, 67.

51. Driver, R.J., Lamb, A.L., Wyner, A.J., and Raizen, D.M. (2013). DAF-16/FOXO regulates homeostasis of essential sleep-like behavior during larval transitions in C. elegans. Curr. Biol. *23*, 501–506.

52. Nagy, S., Tramm, N., Sanders, J., Iwanir, S., Shirley, I.A., Levine, E., and Biron, D. (2014). Homeostasis in C. elegans sleep is characterized by two behaviorally and genetically distinct mechanisms. eLife *3*, e04380.

53. Baugh, L.R., and Sternberg, P.W. (2006). DAF-16/FOXO regulates transcription of cki-1/Cip/Kip and repression of lin-4 during C. elegans L1 arrest. Curr. Biol. *16*, 780–785.

54. Paradis, S., and Ruvkun, G. (1998). Caenorhabditis elegans Akt/PKB transduces insulin receptor-like signals from AGE-1 PI3 kinase to the DAF-16 transcription factor. Genes Dev. *12*, 2488–2498.

55. Hardie, D.G. (2011). AMP-activated protein kinase: an energy sensor that regulates all aspects of cell function. Genes Dev. *25*, 1895–1908.

56. Fukuyama, M., Sakuma, K., Park, R., Kasuga, H., Nagaya, R., Atsumi, Y., Shimomura, Y., Takahashi, S., Kajiho, H., Rougvie, A., et al. (2012). C. elegans AMPKs promote survival and arrest germline development during nutrient stress. Biol. Open *1*, 929–936.

57. Berdichevsky, A., Viswanathan, M., Horvitz, H.R., and Guarente, L. (2006). C. elegans SIR-2.1 interacts with 14-3-3 proteins to activate DAF-16 and extend life span. Cell *125*, 1165–1177.

58. Curtis, R., O'Connor, G., and DiStefano, P.S. (2006). Aging networks in Caenorhabditis elegans: AMP-activated protein kinase (aak-2) links multiple aging and metabolism pathways. Aging Cell *5*, 119–126.

59. Lin, K., Hsin, H., Libina, N., and Kenyon, C. (2001). Regulation of the Caenorhabditis elegans longevity protein DAF-16 by insulin/IGF-1 and germline signaling. Nat. Genet. *28*, 139–145.

60. Libina, N., Berman, J.R., and Kenyon, C. (2003). Tissue-specific activities of C. elegans DAF-16 in the regulation of lifespan. Cell *115*, 489–502.

61. Ehlen, J.C., Brager, A.J., Baggs, J., Pinckney, L., Gray, C.L., DeBruyne, J.P., Esser, K.A., Takahashi, J.S., and Paul, K.N. (2017). *Bmal1* function in skeletal muscle regulates sleep. eLife *6*, e26557.

62. Laranjeiro, R., Harinath, G., Burke, D., Braeckman, B.P., and Driscoll, M. (2017). Single swim sessions in C. elegans induce key features of mammalian exercise. BMC Biol. *15*, 30.

63. Narbonne, P., and Roy, R. (2009). Caenorhabditis elegans dauers need LKB1/AMPK to ration lipid reserves and ensure long-term survival. Nature *457*, 210–214.

64. Kaeberlein, T.L., Smith, E.D., Tsuchiya, M., Welton, K.L., Thomas, J.H., Fields, S., Kennedy, B.K., and Kaeberlein, M. (2006). Lifespan extension in Caenorhabditis elegans by complete removal of food. Aging Cell *5*, 487–494.

65. Roux, A.E., Langhans, K., Huynh, W., and Kenyon, C. (2016). Reversible age-related phenotypes induced during larval quiescence in C. elegans. Cell Metab. *23*, 1113–1126.

66. Yasuda, K., Ishii, T., Suda, H., Akatsuka, A., Hartman, P.S., Goto, S., Miyazawa, M., and Ishii, N. (2006). Age-related changes of mitochondrial structure and function in Caenorhabditis elegans. Mech. Ageing Dev. *127*, 763–770.

67. Morley, J.F., Brignull, H.R., Weyers, J.J., and Morimoto, R.I. (2002). The threshold for polyglutamine-expansion protein aggregation and cellular toxicity is dynamic and influenced by aging in Caenorhabditis elegans. Proc. Natl. Acad. Sci. USA *99*, 10417–10422.

68. David, D.C., Ollikainen, N., Trinidad, J.C., Cary, M.P., Burlingame, A.L., and Kenyon, C. (2010). Widespread protein aggregation as an inherent part of aging in C. elegans. PLoS Biol. *8*, e1000450.

69. Campagnola, P.J., Millard, A.C., Terasaki, M., Hoppe, P.E., Malone, C.J., and Mohler, W.A. (2002). Three-dimensional high-resolution second-

harmonic generation imaging of endogenous structural proteins in biological tissues. Biophys. J. *82*, 493–508.

70. Gems, D., Sutton, A.J., Sundermeyer, M.L., Albert, P.S., King, K.V., Edgley, M.L., Larsen, P.L., and Riddle, D.L. (1998). Two pleiotropic classes of daf-2 mutation affect larval arrest, adult behavior, reproduction and longevity in Caenorhabditis elegans. Genetics *150*, 129–155.
71. Schmidt, M.H. (2014). The energy allocation function of sleep: a unifying theory of sleep, torpor, and continuous wakefulness. Neurosci. Biobehav. Rev. *47*, 122–153.
72. Mackiewicz, M., Shockley, K.R., Romer, M.A., Galante, R.J., Zimmerman, J.E., Naidoo, N., Baldwin, D.A., Jensen, S.T., Churchill, G.A., and Pack, A.I. (2007). Macromolecule biosynthesis: a key function of sleep. Physiol. Genomics *31*, 441–457.
73. Tu, B.P., and McKnight, S.L. (2006). Metabolic cycles as an underlying basis of biological oscillations. Nat. Rev. Mol. Cell Biol. *7*, 696–701.
74. Diekelmann, S., and Born, J. (2010). The memory function of sleep. Nat. Rev. Neurosci. *11*, 114–126.
75. Krause, A.J., Simon, E.B., Mander, B.A., Greer, S.M., Saletin, J.M., Goldstein-Piekarski, A.N., and Walker, M.P. (2017). The sleep-deprived human brain. Nat. Rev. Neurosci. *18*, 404–418.
76. Roffwarg, H.P., Muzio, J.N., and Dement, W.C. (1966). Ontogenetic development of the human sleep-dream cycle. Science *152*, 604–619.
77. Kayser, M.S., Yue, Z., and Sehgal, A. (2014). A critical period of sleep for development of courtship circuitry and behavior in Drosophila. Science *344*, 269–274.
78. Sulston, J.E., and Horvitz, H.R. (1977). Post-embryonic cell lineages of the nematode, Caenorhabditis elegans. Dev. Biol. *56*, 110–156.
79. Krueger, J.M., Frank, M.G., Wisor, J.P., and Roy, S. (2016). Sleep function: toward elucidating an enigma. Sleep Med. Rev. *28*, 46–54.
80. Labrousse, A., Chauvet, S., Couillault, C., Kurz, C.L., and Ewbank, J.J. (2000). Caenorhabditis elegans is a model host for Salmonella typhimurium. Curr. Biol. *10*, 1543–1545.
81. Nguyen, J.P., Linder, A.N., Plummer, G.S., Shaevitz, J.W., and Leifer, A.M. (2017). Automatically tracking neurons in a moving and deforming brain. PLoS Comput. Biol. *13*, e1005517.
82. Mair, W., Morantte, I., Rodrigues, A.P., Manning, G., Montminy, M., Shaw, R.J., and Dillin, A. (2011). Lifespan extension induced by AMPK and calcineurin is mediated by CRTC-1 and CREB. Nature *470*, 404–408.
83. Fire, A., Xu, S., Montgomery, M.K., Kostas, S.A., Driver, S.E., and Mello, C.C. (1998). Potent and specific genetic interference by double-stranded RNA in Caenorhabditis elegans. Nature *391*, 806–811.
84. Kramer, J.M., and Johnson, J.J. (1993). Analysis of mutations in the sqt-1 and rol-6 collagen genes of Caenorhabditis elegans. Genetics *135*, 1035–1045.
85. Zhang, P., Judy, M., Lee, S.J., and Kenyon, C. (2013). Direct and indirect gene regulation by a life-extending FOXO protein in C. elegans: roles for GATA factors and lipid gene regulators. Cell Metab. *17*, 85–100.
86. Urmersbach, B., Besseling, J., Spies, J.P., and Bringmann, H. (2016). Automated analysis of sleep control via a single neuron active at sleep onset in C. elegans. Genesis *54*, 212–219.
87. Brenner, S. (1974). The genetics of Caenorhabditis elegans. Genetics *77*, 71–94.
88. Ahringer, A. (2006). Reverse genetics. WormBook 1–43.
89. Merritt, C., and Seydoux, G. (2010). Transgenic solutions for the germline. WormBook, 1–21.
90. Redemann, S., Schloissnig, S., Ernst, S., Pozniakowsky, A., Ayloo, S., Hyman, A.A., and Bringmann, H. (2011). Codon adaptation-based control of protein expression in C. elegans. Nat. Methods *8*, 250–252.
91. Wilm, T., Demel, P., Koop, H.U., Schnabel, H., and Schnabel, R. (1999). Ballistic transformation of Caenorhabditis elegans. Gene *229*, 31–35.
92. Praitis, V., Casey, E., Collar, D., and Austin, J. (2001). Creation of low-copy integrated transgenic lines in Caenorhabditis elegans. Genetics *157*, 1217–1226.
93. Golden, J.W., and Riddle, D.L. (1984). The Caenorhabditis elegans dauer larva: developmental effects of pheromone, food, and temperature. Dev. Biol. *102*, 368–378.
94. Stiernagle, T. (2006). Maintenance of C. elegans. WormBook, 1–11.
95. Nagy, S., Raizen, D.M., and Biron, D. (2014). Measurements of behavioral quiescence in Caenorhabditis elegans. Methods *68*, 500–507.
96. Lewis, J.A., and Fleming, J.T. (1995). Basic culture methods. Methods Cell Biol. *48*, 3–29.
97. Jung, J., Nakajima, M., Kojima, M., Ooe, K., and Fukuda, T. (2012). Microchip device for measurement of body volume of *C. elegans* as bioindicator application. J. Micro-Nano Mech. *7*, 3–11.
98. Herndon, L.A., Schmeissner, P.J., Dudaronek, J.M., Brown, P.A., Listner, K.M., Sakano, Y., Paupard, M.C., Hall, D.H., and Driscoll, M. (2002). Stochastic and genetic factors influence tissue-specific decline in ageing C. elegans. Nature *419*, 808–814.

STAR★METHODS

KEY RESOURCES TABLE

REAGENT or RESOURCE	SOURCE	IDENTIFIER
Bacterial and Virus Strains		
OP50 *Escherichia coli* B, Uracil auxotroph	Caenorhabditis Genetics Center	OP50
OP50 *Escherichia coli* that contains a GFP plasmid (pFPV25.1)	Caenorhabditis Genetics Center [80]	OP50-GFP
Chemicals, Peptides, and Recombinant Proteins		
FUDR, 5-Fluoro-2′-deoxyuridine	Sigma-Aldrich	F0503
Levamisole hydrochloride	Sigma-Aldrich	PHR1798
Experimental Models: Organisms/Strains		
N2: *wild type*	Caenorhabditis Genetics Center	N2
HBR227: *aptf-1(gk794)* II.	[29]	HBR227
HBR1361: *goeIs304[pflp-11::SL1-GCaMP3.35-SL2::mKate2-unc-54-3′UTR, unc-119(+)].*	This paper	HBR1361
HBR1374: *goeIs307[pflp-11::ArchT::SL2mKate2-unc-54-3′UTR,unc-119(+)]; goeIs304[pflp-11::SL1-GCaMP3.35-SL2::mKate2-unc-54-3′UTR, unc-119(+)].*	This paper	HBR1374
HBR1709: *daf-16(mgDf50) I; goeIs304[pflp-11::SL1-GCaMP3.35-SL2::mKate2-unc-54-3′UTR, unc-119(+)].*	This paper and Caenorhabditis Genetics Center	HBR1709
HBR1753: *wtfIs5[prab-3::NLS::GCaMP6s; prab-3::NLS::tagRFP]*	This paper, created from AML32 (Andrew Leifer lab) [81]	HBR1753
HBR1777: *goeIs384 [pflp-11::egl-1::SL2-mkate2-flp-11-3′UTR, unc-119(+)].*	This paper	HBR1777
HBR1807: *goeIs232[psra-6::ReaChr::mKate2-unc-54-3′UTR, unc-119(+)]; goeIs304[pflp-11::SL1-GCaMP3.35-SL2::mKate2-unc-54-3′UTR, unc-119(+)].*	This paper	HBR1807
HBR1808: *goeIs251[pmec-4::ReaChr::mKate2-unc-54-3′UTR,unc-119(+)]; goeIs304[pflp-11::SL1-GCaMP3.35-SL2::mKate2-unc-54-3′UTR, unc-119(+)].*	This paper	HBR1808
HBR1830: *daf-2(e1370) III; goeIs304[pflp-11::SL1-GCaMP3.35-SL2::mKate2-unc-54-3′UTR, unc-119(+)].*	This paper and Caenorhabditis Genetics Center	HBR1830
HBR2005: *aak-1(tm1944) III; aak-2(ok524) X; goeIs304[pflp-11::SL1-GCaMP3.35-SL2::mKate2-unc-54-3′UTR, unc-119(+)].*	This paper and Caenorhabditis Genetics Center	HBR2005
HBR2006: *daf-16(mgDf50) I; aak-1(tm1944) III; aak-2(ok524) X; goeIs304[pflp-11::SL1-GCaMP3.35-SL2::mKate2-unc-54-3′UTR, unc-119(+)].*	This paper and Caenorhabditis Genetics Center	HBR2006
HBR2007: *daf-16(mgDf50) I; aak-1(tm1944) III; goeIs304[pflp-11::SL1-GCaMP3.35-SL2::mKate2-unc-54-3′UTR, unc-119(+)].*	This paper and Caenorhabditis Genetics Center	HBR2007
HBR2009: *daf-16(mgDf50) I; aak-2(ok524) X; goeIs304[pflp-11::SL1-GCaMP3.35-SL2::mKate2-unc-54-3′UTR, unc-119(+)].*	This paper and Caenorhabditis Genetics Center	HBR2009
HBR2020: *daf-16(mgDf50) I; aptf-1(gk794) II; aak-1(tm1944) III; aak-2(ok524) X.*	This paper and Caenorhabditis Genetics Center	HBR2020
HBR2030: *aptf-1(gk794) II; aak-1(tm1944) III; aak-2(ok524) X.*	This paper and Caenorhabditis Genetics Center	HBR2030
AGD397: *aak-1(tm1944) III; aak-2(ok524) X; uthEx202 [crtc-1p::crtc-1 cDNA::tdTomato::unc-54 3′UTR + rol-6(su1006)]* (only non-*rol-6* animals were used)	Andrew Dillin lab, Caenorhabditis Genetics Center [82]	AGD397
CF1295: *daf-16(mu86) I; daf-2(e1370) III; muEx108[pKL99-2(daf-16::GFP/daf16bKO) + pRF4(rol-6)]* (only non-*rol-6* animals were used)	Cynthia Kenyon lab, Caenorhabditis Genetics Center [59]	CF1295
AM140: *rmIs132 [punc-54::Q35::YFP] I.*	Richard Morimoto lab, Caenorhabditis Genetics Center [67]	AM140

(*Continued on next page*)

Continued

REAGENT or RESOURCE	SOURCE	IDENTIFIER
PD4251: *ccIs4251 [(pSAK2) pmyo-3::GFP::LacZ::NLS+(pSAK4)pmyo-3::mitochondrialGFP +dpy-20(+)] I.*	Andrew Fire lab, Caenorhabditis Genetics Center [83]	PD4251
RW1596: *myo-3(st386) V; stEx30[pmyo-3::GFP + rol-6(su1006)].*	Robert Waterston lab, Caenorhabditis Genetics Center [67]	RW1596
HBR1617: *rmIs132[punc-54::Q35::YFP] I; aptf-1(gk794) II.*	This paper and [29, 67]	HBR1617
HBR1618: *ccIs4251[(pSAK2) pmyo-3::GFP::LacZ::NLS + (pSAK4) pmyo-3::mitochondrial GFP + dpy-20(+)] I; aptf-1(gk794) II; dpy-20(e1282) IV.*	This paper and [29, 83]	HBR1618
HBR1516: *aptf-1(gk794) II; myo-3(st386) V; stEx30[pmyo-3::GFP + rol-6(su1006)].*	This paper and [29, 67]	HBR1516
HBR2098: *daf-18(ok480) IV.*	This paper and Caenorhabditis Genetics Center	HBR2098
HBR2100: *sir-2.1(ok434) IV.*	This paper and Caenorhabditis Genetics Center	HBR210
HE1006*: rol-6(su1006) II.*	Henry Epstein lab, Caenorhabditis Genetics Center [84]	HE1006
CF1724*: daf-16(mu86) I; daf-2(e1370) III; muIs105 [pdaf-16::GFP::daf-16 + rol-6(su1006)].*	Cynthia Kenyon lab, Caenorhabditis Genetics Center [59]	CF1724
CF2093*: daf-16(mu86) I; daf-2(e1370) III; muIs131 [punc-119::GFP::daf-16 + rol-6(su1006)].*	Cynthia Kenyon lab, Caenorhabditis Genetics Center [85]	CF2093
CF2093*: daf-16(mu86) I; daf-2(e1370) III; muIs131 [punc-119::GFP::daf-16 + rol-6(su1006)].*	Cynthia Kenyon lab, Caenorhabditis Genetics Center [85]	CF2093
CF2102*: daf-16(mu86) I; daf-2(e1370) III; muIs126 [pmyo-3::GFP::daf-16 + rol-6(su1006)].*	Cynthia Kenyon lab, Caenorhabditis Genetics Center [85]	CF2102
CF2005*: daf-16(mu86) I; daf-2(e1370) III; muIs120 [pges-1::GFP::daf-16 + rol-6(su1006)].*	Cynthia Kenyon lab, Caenorhabditis Genetics Center [85]	CF2005
CF2570*: daf-16(mu86) I; daf-2(e1370) III; muIs142[pges-1::GFP::daf-16 + odr-1p::RFP].*	Cynthia Kenyon lab, Caenorhabditis Genetics Center [85]	CF2570
HBR2158*: daf-16(mgDf50) I; aak-2(ok524) X; tdEx618[ppgp-1::RFP+rol-6(su1006)]; goeIs304[pflp-11::SL1-GCaMP3.35-SL2::mKate2-unc-54-3′UTR, unc-119(+)].*	This paper and Masamitsu Fukuyama lab [56]	HBR2158
HBR2159*: daf-16(mgDf50) I; aak-2(ok524) X; tdEx589[pMF342, pmyo-3::GFP::aak-2::unc-54 3′UTR]+ppgp-1::RFP +rol-6(su1006)].*	This paper and Masamitsu Fukuyama lab [56]	HBR2159
HBR2160*: daf-16(mgDf50) I; aak-2(ok524) X; tdEx700[pMF380ppgp-1::GFP::aak-2::unc-54 3′UTR]+ppgp-1::RFP+rol-6(su1006)].*	This paper and Masamitsu Fukuyama lab [56]	HBR2160
HBR2161*: daf-16(mgDf50) I; aak-2(ok524) X; tdEx632[pMF308prgef-1::GFP::aak-2::unc-54 3′UTR+ppgp-1::RFP+rol-6(su1006)].*	This paper and Masamitsu Fukuyama lab [56]	HBR2161
HBR2162*: daf-16(mgDf50) I; aak-2(ok524) X; tdEx541[pMF312, pdpy-7::GFP::aak-2::unc-54 3′UTR+ppgp-1::RFP+rol-6(su1006)].*	This paper and Masamitsu Fukuyama lab [56]	HBR2162
HBR2163*: daf-16(mgDf50) I; aak-2(ok524) X; tdEx679[pKS19, paak-2::aak-2::GFP::unc-86 3′UTR+ppgp-1::RFP+rol-6(su1006)].*	This paper and Masamitsu Fukuyama lab [56]	HBR2163
HBR2173*: daf-16(mgDf50), aak-2 (ok524) X; goeEx720 [pflp-11::aak-2a::SL2-mKate::flp-11 3′UTR, unc-119(+) + myo-2::mCherry]; [ppgp-1::RFP+rol-6(su1006)]; goeIs304[pflp-11::SL1-GCaMP3.35-SL2::mKate2-unc-54-3′UTR, unc-119(+)].*	This paper and Caenorhabditis Genetics Center	HBR2173
HBR2151*: daf-16(mgDf50), aak-2 (ok524) X; rrEx127[paak-2::aak-2+rol-6(su1006)].*	This paper and Richard Roy lab [63]	HBR2151
HBR2152*: daf-16(mgDf50), aak-2 (ok524) X; rrEx191[psulp-5::AAK-2+rol-6(su1006)]* [63].	This paper and Richard Roy lab [63]	HBR2152
HBR2153*: daf-16(mgDf50), aak-2 (ok524) X; rrEx123[pelt-2::aak-2+rol-6(su1006)]* [63].	This paper and Richard Roy lab [63]	HBR2153
HBR2154*: daf-16(mgDf50), aak-2 (ok524) X; rrEx120[psur-5::aak-2+rol-6(su1006)]* [63].	This paper and Richard Roy lab [63]	HBR2154

(*Continued on next page*)

Continued		
REAGENT or RESOURCE	SOURCE	IDENTIFIER
HBR2155: *daf-16(mgDf50), aak-2 (ok524) X; rrEx122[punc-54::aak-2+rol-6(su1006)]* [63].	This paper and Richard Roy lab [63]	HBR2155
HBR2156: *daf-16(mgDf50), aak-2 (ok524) X; rrEx114[punc-119::aak-2+rol-6(su1006)]* [63].	This paper and Richard Roy lab [63]	HBR2156
HBR2157: *daf-16(mgDf50), aak-2 (ok524) X; rrEx193[pdpy-7p::aak-2+psur-5::GFP]* [63].	This paper and Richard Roy lab [63]	HBR2157
Oligonucleotides		
For a list of primers see Table S7	N/A	N/A
Recombinant DNA		
Plasmid K171: *pmec-4::ReaChr::mKate2-unc-54-3′utr, unc-119(+)*	This paper	K171
Plasmid K172: *psra-6::ReaChr::mKate2-unc-54-3′utr, unc-119(+)*	This paper	K172
Plasmid K214: *pflp-11::ArchT::SL2-mKate2-unc-54-3′utr,unc-119(+)*	This paper	K214
Plasmid K216: *pflp-11::SL1-GCaMP3.35-SL2::mKate2-unc-54-3′utr, unc-119(+)*	This paper	K216
Plasmid K281: *pflp-11::egl-1::SL2-mKate2-flp-11-3′utr, unc-119(+)*	This paper	K281
Plasmid K358: *pflp-11::aak-2a::SL2-mKate2-flp-11 3′utr, unc-119(+)*	This paper	K358
Software and Algorithms		
MATLAB R2017a	Mathworks	9.2.0.538062
OriginPro 2017	Originlab	SR1 b9.4.1.354 (64-bit)
Excel 2016	Microsoft	MSO 16.0.4738.1000 (32-bit)
Wormtracker	Urmersbach et al. [86]	Wormtracker
ImageJ-win64	Fiji - ImageJ	1.52b, Java 1.6.0_24 (64-bit)
Andor iQ2	Andor	iQ 2.9.1
R	http://cran.r-project.org	R 3.3.2, GUI 1.68
NIS-Elements	Nikon	AR 5.02.00 (64-bit)

CONTACT FOR REAGENT AND RESOURCE SHARING

Further information and requests for resources and reagents should be directed to and will be fulfilled by the Lead Contact, Henrik Bringmann (Henrik.Bringmann@mpibpc.mpg.de).

EXPERIMENTAL MODEL AND SUBJECT DETAILS

Worm maintenance and strains

C. elegans was grown on Nematode Growth Medium (NGM) agarose plates seeded with *E. coli* OP50 and were kept at 20°C [87]. For crossings, the strains were genotyped using Duplex PCR genotyping of single worms [88]. Primer sequences used for Duplex PCR can be found in Table S7. Following *C. elegans* strains were used:

N2: *wild-type*
HBR227: *aptf-1(gk794)* II.
HBR1361: *goeIs304[pflp-11::SL1-GCaMP3.35-SL2::mKate2-unc-54-3′UTR, unc-119(+)]*.
HBR1374: *goeIs307[pflp-11::ArchT::SL2mKate2-unc-54-3′UTR,unc-119(+)]; goeIs304[pflp-11::SL1-GCaMP3.35-SL2::mKate2-unc-54-3′UTR, unc-119(+)]*.
HBR1709: *daf-16(mgDf50) I; goeIs304[pflp-11::SL1-GCaMP3.35-SL2::mKate2-unc-54-3′UTR, unc-119(+)]*.
HBR1753: *wtfIs5[prab-3::NLS::GCaMP6s; prab-3::NLS::tagRFP]* (created from AML32 (a gift from Andrew Leifer) by backcrossing 2x into N2) [81].
HBR1777: *goeIs384 [pflp-11::egl-1::SL2-mkate2-flp-11-3′UTR, unc-119(+)]*.
HBR1807: *goeIs232[psra-6::ReaChr::mKate2-unc-54-3′UTR, unc-119(+)]; goeIs304[pflp-11::SL1-GCaMP3.35-SL2::mKate2-unc-54-3′UTR, unc-119(+)]*.
HBR1808: *goeIs251[pmec-4::ReaChr::mKate2-unc-54-3′UTR,unc-119(+)]; goeIs304[pflp-11::SL1-GCaMP3.35-SL2::mKate2-unc-54-3′UTR, unc-119(+)]*.

HBR1830: *daf-2(e1370) III; goeIs304[pflp-11::SL1-GCaMP3.35-SL2::mKate2-unc-54-3′UTR, unc-119(+)]*.
HBR2005: *aak-1(tm1944) III; aak-2(ok524) X; goeIs304[pflp-11::SL1-GCaMP3.35-SL2::mKate2-unc-54-3′UTR, unc-119(+)]*.
HBR2006: *daf-16(mgDf50) I; aak-1(tm1944) III; aak-2(ok524) X; goeIs304[pflp-11::SL1-GCaMP3.35-SL2::mKate2-unc-54-3′UTR, unc-119(+)]*.
HBR2007: *daf-16(mgDf50) I; aak-1(tm1944) III; goeIs304[pflp-11::SL1-GCaMP3.35-SL2::mKate2-unc-54-3′UTR, unc-119(+)]*.
HBR2009: *daf-16(mgDf50) I; aak-2(ok524) X; goeIs304[pflp-11::SL1-GCaMP3.35-SL2::mKate2-unc-54-3′UTR, unc-119(+)]*.
HBR2020: *daf-16(mgDf50) I; aptf-1(gk794) II; aak-1(tm1944) III; aak-2(ok524) X*.
HBR2030: *aptf-1(gk794) II; aak-1(tm1944) III; aak-2(ok524) X*.
AGD397: *aak-1(tm1944) III; aak-2(ok524) X; uthEx202 [crtc-1p::crtc-1 cDNA::tdTomato::unc-54 3′UTR + rol-6(su1006)]* (only non-rol-6 animals were used) [82].
CF1295: *daf-16(mu86) I; daf-2(e1370) III; muEx108[pKL99-2(daf-16::GFP/daf16bKO) + pRF4(rol-6)]* (only non-rol-6 animals were used) [59].
AM140: *rmIs132 [punc-54::Q35::YFP] I* [67].
PD4251: *ccIs4251 [(pSAK2)pmyo-3::GFP::LacZ::NLS+(pSAK4)pmyo-3::mitochondrialGFP +dpy-20(+)] I* [83].
RW1596: *myo-3(st386) V; stEx30[pmyo-3::GFP + rol-6(su1006)]* [69].
HBR1617: *rmIs132[punc-54::Q35::YFP] I; aptf-1(gk794) II*.
HBR1618: *ccIs4251[(pSAK2) pmyo-3::GFP::LacZ::NLS + (pSAK4) pmyo-3::mitochondrial GFP + dpy-20(+)] I; aptf-1(gk794) II; dpy-20(e1282) IV*.
HBR1516: *aptf-1(gk794) II; myo-3(st386) V; stEx30[pmyo-3::GFP + rol-6(su1006)]*.
HBR2098*: daf-18(ok480) IV.* created from RB712 by backcrossing 2x into N2
HBR2100*: sir-2.1(ok434) IV.* created from VC199 by backcrossing 2x into N2
HE1006: *rol-6(su1006) II* [84].
CF1724: *daf-16(mu86) I; daf-2(e1370) III; muIs105 [pdaf-16::GFP::daf-16 + rol-6(su1006)]* [59].
CF2093: *daf-16(mu86) I; daf-2(e1370) III; muIs131 [punc-119::GFP::daf-16 + rol-6(su1006)]* [85].
CF2102: *daf-16(mu86) I; daf-2(e1370) III; muIs126 [pmyo-3::GFP::daf-16 + rol-6(su1006)]* [85].
CF2005: *daf-16(mu86) I; daf-2(e1370) III; muIs120 [pges-1::GFP::daf-16 + rol-6(su1006)]* [85].
CF2570: *daf-16(mu86) I; daf-2(e1370)III; muIs142[pges-1::GFP::daf-16 + odr-1p::RFP]* [85].
HBR2158: *daf-16(mgDf50) I; aak-2(ok524) X; tdEx618[ppgp-1::RFP+rol-6(su1006)]; goeIs304[pflp-11::SL1-GCaMP3.35-SL2::mKate2-unc-54-3′UTR, unc-119(+)]* [56].
HBR2159: *daf-16(mgDf50) I; aak-2(ok524) X; tdEx589[pMF342, pmyo-3::GFP::aak-2::unc-54 3′UTR]+ppgp-1::RFP+rol-6(su1006)]* [56].
HBR2160: *daf-16(mgDf50) I; aak-2(ok524) X; tdEx700[pMF380ppgp-1::GFP::aak-2::unc-54 3′UTR]+ppgp-1::RFP+rol-6(su1006)]* [56].
HBR2161: *daf-16(mgDf50) I; aak-2(ok524) X; tdEx632[pMF308prgef-1::GFP::aak-2::unc-54 3′UTR+ppgp-1::RFP+rol-6(su1006)]* [56].
HBR2162: *daf-16(mgDf50) I; aak-2(ok524) X; tdEx541[pMF312, pdpy-7::GFP::aak-2::unc-54 3′UTR+ppgp-1::RFP+rol-6(su1006)]* [56].
HBR2163: *daf-16(mgDf50) I; aak-2(ok524) X; tdEx679[pKS19, paak-2::aak-2::GFP::unc-86 3′UTR+ppgp-1::RFP+rol-6(su1006)]* [56].
HBR2173: *daf-16(mgDf50), aak-2 (ok524) X; goeEx720 [pflp-11::aak-2a::SL2-mKate::flp-11 3′UTR, unc-119(+) + myo-2::mCherry]; [ppgp-1::RFP+rol-6(su1006)]; goeIs304[pflp-11::SL1-GCaMP3.35-SL2::mKate2-unc-54-3′UTR, unc-119(+)].*
HBR2151: *daf-16(mgDf50), aak-2 (ok524) X; rrEx127[paak-2::aak-2+rol-6(su1006)]* [63].
HBR2152: *daf-16(mgDf50), aak-2 (ok524) X; rrEx191[psulp-5::AAK-2+rol-6(su1006)]* [63].
HBR2153: *daf-16(mgDf50), aak-2 (ok524) X; rrEx123[pelt-2::aak-2+rol-6(su1006)]* [63].
HBR2154: *daf-16(mgDf50), aak-2 (ok524) X; rrEx120[psur-5::aak-2+rol-6(su1006)]* [63].
HBR2155: *daf-16(mgDf50), aak-2 (ok524) X; rrEx122[punc-54::aak-2+rol-6(su1006)]* [63].
HBR2156: *daf-16(mgDf50), aak-2 (ok524) X; rrEx114[punc-119::aak-2+rol-6(su1006)]* [63].
HBR2157: *daf-16(mgDf50), aak-2 (ok524) X; rrEx193[pdpy-7p::aak-2+psur-5::GFP]* [63].

METHOD DETAILS

Molecular biology and transgenic strain generation

We cloned all constructs into the pCG150 Vector that contains *unc-119(+)* [89] by using the Three Fragment Gateway System (Invitrogen, Carlsbad, CA). For verification, the cloned constructs were sequenced. The GCaMP3.35, the ReaChr, the ArchT and the *egl-1* genes were expression-optimized for *C. elegans* [90]. The *pflp-11::egl-1* transgene specifically expresses the apoptosis inducing protein EGL-1 in RIS. Thus, strains carrying this transgene have a genetic ablation of RIS. For AMPK rescue a cDNA corresponding to *aak-2a* was used and was obtained by synthesizing the gene based on the available gene sequence (Wormbase release WS266). We generated the transgenic strains by microparticle bombardment into *unc-119(ed3)* mutant worms and used phenotypic rescue of

the *unc* phenotype as a selection marker [91, 92]. The insertions that were obtained were backcrossed two times against N2 to remove the *unc-119(ed3)* background. The following plasmids were created for this study:

K171: *pmec-4::ReaChr::mKate2-unc-54-3′utr, unc-119(+)*
K172: *psra-6::ReaChr::mKate2-unc-54-3′utr, unc-119(+)*
K214: *pflp-11::ArchT::SL2-mKate2-unc-54-3′utr,unc-119(+)*
K216: *pflp-11::SL1-GCaMP3.35-SL2::mKate2-unc-54-3′utr, unc-119(+)*
K281: *pflp-11::egl-1::SL2-mKate2-flp-11-3′utr, unc-119(+)*
K358: *pflp-11::aak-2a::SL2-mKate2-flp-11 3′utr, unc-119(+)*

Dauer pheromone extraction and dauer larvae generation

The crude dauer pheromone was extracted as described previously [93]. Briefly, worms were cultured in 1L of S-medium with resuspended OP50 at 25°C. When the culture clarified as a consequence of bacteria depletion, additional OP50 was added. When cleared the second time, the supernatant was filtered and boiled to a solid crust. This crust was extracted three times with ethanol. After evaporation of the ethanol, the dauer pheromone was resuspended in 1 mL of sterile water and stored at −20°C. A 10 μL drop of water containing dauer pheromone was placed on the solidified agarose, which contained the microchambers. The drop was placed after sealing the chambers with a coverslip. The drop was pipetted onto the surface that did not contain any microchambers and subsequently diffused into the agar. To obtain dauer larvae, NGM plates were left to starve at 25°C and daily controlled for occurrence of dauer larvae. 3 days after the first dauers occurred, the starved NGM plate was chunked to a fresh unseeded NGM plate and the dauers were allowed to crawl off that chunk. From there, individual dauer larvae were isolated and used for behavioral experiments

Long-term imaging

For long-term imaging, agarose microchamber imaging was used [34, 35]. Briefly, box-shaped indentations in agarose hydrogel were cast using a PDMS mold. The chambers were then filled with worms and for fed conditions with bacteria, and sealed with a glass coverslip. 3% agarose dissolved in S-Basal [94] was used as a hydrogel in all experiments to starve worms and bacteria except for the experiments were we investigated behavior in the presence of growth medium. To obtain growth medium conditions we mixed Nematode Growth Medium [94] and S-Basal 1:1, added 3% agarose, and casted microchambers from this mix. To obtain dead bacteria, the OP50 lawn was pasteurized on seeded NGM plates for 3 hr in an oven set to 70°C. For pheromone experiments, 10 μL of dauer pheromone extract was added on top of the agarose microchamber, which then diffused into the agarose. Behavioral and functional calcium imaging [29, 86] was performed simultaneously. In short, we used an Andor iXon (512x512 pixels) EMCCD camera and LED illumination (CoolLed) using standard GFP filter sets. The exposure time was set to 5ms. The EMCCD "TTL fire out" signal was used to trigger the LED illumination in order to illuminate only during exposure. The 490nm light intensity was 2.00mW/mm^2 using a 20x objective and 0.60mW/mm^2 using a 10x objective. EM gain was set to 100. Using these parameters, we obtained image sequences with clearly identifiable worm outlines and measurable neuronal calcium transients.

Typically, 8-12 individual fields in close vicinity were filmed. Adult worms were cultured in 700μm x 700μm x 45μm (X length x Y length x Z depth) microchambers. Dauer larvae were kept in 370μm x 370μm x 25μm microchambers and L1 larvae in 190μm x 190μm x 15μm unless otherwise mentioned. For adults and dauer larvae, one imaged field included an individual worm in a single microchamber. For L1s, one filmed field can include up to four worms in adjacent microchambers. Adults were imaged using the 10x objective. L1s and dauer were imaged with the 20x objective. An automatic stage (Prior Proscan 2 or 3) moved repeatedly to the microchambers using low acceleration speeds. The frame rate we obtained was 0.1frame/s unless otherwise noted. Worms were filmed either continuously or in series of movies for optogenetic experiments.

In the L1 experiments, pretzel stage eggs were picked with an eyelash or eyebrow hair to an empty NGM plate to remove remaining bacteria. From this empty NGM plate, the eggs were transferred to the microchamber again using a clean hair. Image acquisition was started 24 hr after hatching. Dauers were obtained from plates that were left to starve at 25°C. To isolate individual dauers, we cut out agarose chunks of the starved plates and transferred them to an empty NGM plate and allowed the worms to crawl off the agarose chunk. Individual dauers were pipetted with 1-5μL of water to the microchamber. With a pick, the dauers were distributed over the microchambers so that each dauer was placed into its own chamber. We assayed dauers that were 3 days old. Adults for starvation, *daf-2(e1370)* mutants and its controls were treated with 5-fluoro-2-deoxyuridine (FUdR, Sigma-Aldrich) before the experiments. For this, L4s were transferred to seeded NGM plates containing 50μM FUdR and 50mg/L kanamycin (Sigma-Aldrich) [64]. 24 hr after the FUdR treatment, we transferred the adults to the microchamber. The worms were either transferred with a worm pick into a microchamber filled with OP50 for feeding experiments or they were pipetted in a 1-5μL drop of water to a microchamber for starvation experiments. For starvation experiments, worms were kept without food for 24 hr in the empty microchambers before image acquisition. For *daf-2(e1370)* mutant experiments, imaging was started 35 hr after shifting the temperature from 15°C to 23°C. Imaging was started 6 hr after the pheromone application. Imaging was started 3 hr after the microchamber was prepared. To allow identification of the non-pumping period, additional DIC images were collected.

Optogenetics and light stimulation

All optogenetic experiments using ReaChr and ArchT were performed inside microchambers [29]. Two days before the experiments, the strains were grown on NGM plates supplemented with 0.2mM all-trans retinal (ATR, Sigma-Aldrich). The agarose that was used for microchamber fabrication was also supplemented with ATR of the same concentration. A dual GFP/mCherry excitation/dichroic plus GFP emission filter set (Chroma) was used to allow simultaneous GCaMP imaging and optogenetic manipulation. To activate ReaChr and ArchT, the LED light intensity at 585nm was set to 0.47mW/mm^2 using a 20x-objective and 0.14mW/mm^2 using a 10x-objective. The light intensities were measured using a light voltmeter (PM100A, Thorlabs).

To test for responsiveness to stimulation, we applied blue light irradiation at 490nm with an intensity of 3mW/mm^2 to arrested L1 larvae inside the microfluidic compartments. To evoke a behavioral response we used blue light, which triggers an endogenous light-avoidance response. For these experiments, L1 worms were starved for 24 hr inside microchambers (110μm x 110μm x 10μm) and imaged with 2frames/s. After 1min of baseline imaging, the animals were illuminated for 3min and followed by an additional 1min imaging without illumination. Each animal was assayed up to 16 times with at least 1 hr between two blue light irradiations to allow the worm to recover from the stimulus. To extract sleep-wake-differences of responsiveness, the worm's behavioral state was classified post hoc into either "wake" or "sleep." The worm was classified as "wake," when it was never in a sleep bout during the imaging sequence 3.5 min before the stimulation (see below) throughout the baseline measurement and was classified as "sleep" when it was continuously in a sleep bout during the 3.5 min before stimulation. Only data from time points that continuously met the criteria for being asleep or awake during the 3.5 min before stimulation were taken for the sleep and wake analysis, the other data were excluded. These excluded data were those time points during which the worm showed episodes of both sleep and wake during the 3.5 min of stimulation. The specific numbers of experiments and data exclusions are specified as follows: For Figure 3A, a total of 600 measurements were made. 446 (74.3%) were counted as "wake," 98 (16.3%) were counted as "sleep" and 56 (9.3%) could not be assigned to "wake" or "sleep" and were excluded. For Figure 3C with ATR, a total of 125 measurements were made. 70 (56.0%) were counted as "wake," 19 (15.2%) were counted as "sleep" and 36 (28.8%) could not be assigned to "wake" or "sleep" and were excluded. For Figure 3C without ATR, a total of 273 measurements were made. 110 (40.3%) were scored as "wake," 40 (14.7%) were scored as "sleep" and 123 (40.1%) could not be assigned to "wake" or "sleep" and were excluded. For Figure 3D with ATR, a total of 167 measurements were made. 101 (60.5%) were counted as "wake," 42 (25.1%) were counted as "sleep" and 24 (14.4%) could not be assigned to "wake" or "sleep" and were excluded. For Figure 3D without ATR, a total of 297 measurements were made. 69 (23.2%) were counted as "wake," 85 (28.6%) were counted as "sleep" and 143 (48.1%) could not be assigned to "wake" or "sleep" and were excluded. Overall for Figures 3C and 3D, a total of 862 measurements were made. 350 (40.6%) were counted as "wake," 186 (21.6%) were counted as "sleep" and 326 (37.8%) could not be assigned to "wake" or "sleep" and were excluded. For Figure 3E with stimulus, a total of 180 measurements were made. 80 (44.4%) were counted as "wake," 38 (21.1%) were counted as "sleep" and 62 (34.4%) could not be assigned to "wake" or "sleep" and were excluded. For Figure 3E without stimulus, a total of 131 measurements were made. 32 (24.4%) were counted as "wake," 71 (54.2%) were counted as "sleep" and 28 (21.4%) could not be assigned to "wake" or "sleep" and were excluded.

To measure responsiveness to stimulation, the slope of the linear fit of the movement speed over the first minute of stimulation was used to measure the velocity increase. These velocity increase measurements were averaged for every worm and were subsequently averaged over all worms measured. Data was statistically compared using the Wilcoxon signed rank test for paired samples so as to compare sleep and wake responses.

To test for homeostasis, RIS was optogenetically inhibited in arrested L1 larvae. *flp-11::ArchT* expressing larvae were starved for 24 hr in the microchambers and imaged for 3 hr. During the first hour, baseline data was recorded without illumination. For another hour the data was recorded under green light illumination. The third hour was recorded without green light. For statistical comparison (two-sample t test) between the condition with supplemented ATR and without ATR, we used averaged data ranging from 2.5 – 7.5 min after the end of green light stimulation.

To test for reversibility, arrested L1 larvae that express either *sra-6::ReaChr* or *mec-4::ReaChr* were used 24 hr after hatching in the absence of food. Each animal was imaged for 30 min and illuminated after 15 min for 1500 ms with green light. This was repeated up to 6 times with a delay of at least 1 hr to allow the worm to recover from the stimulus. The reversibility recordings were classified post hoc in "awake" or "asleep." During each data point, the worm was classified as awake, when it spent less than 25% in a sleep bout during the 15min of baseline measurement and was classified as asleep when it spent at least 95% of the time in a sleep bout during the 3.5 min time interval before the optogenetic activation started. Data that could not get clearly classified with the above criteria were excluded from the analysis. Awake and asleep data was averaged for every worm to give one value for wake and one for sleep. Data was then averaged across all worms. For statistical comparisons (two-sample t test), we averaged data from the 5min after the end of optogenetic stimulation.

Image Analysis

We extracted two parameters from the acquired images: 1) worm locomotion velocity: Two methods were used: A) The worm's head position was determined either manually or automatically with a home-made MATLAB routine that detects the position of RIS in the head of the animal, or the centroid of the entire body, similar to the one previously described [86] and calculated with a conversion factor to obtain the velocity in μm/s. This method was applied for Figures 2, 3B, 4C–4F, 5G, S3E, and S6C. B) Sleep bouts were extracted using frame subtraction using a MATLAB routine similarly as previously described [95]. This method was applied for Figure S4. 2) RIS depolarization as measured by GCaMP3 intensity: Using the coordinates for RIS position as determined by the MATLAB

routine, RIS GCaMP3 intensity was extracted from the images. The RIS GCaMP3 intensity was calculated as the mean of the 30 highest pixel intensities in an 11pixel x 11pixel square around the RIS cell body. Pan-neuronal nuclear GCaMP6s intensity was calculated as the mean of the 500 highest pixel intensities in a 69pixel x 69pixel region of interest around the center of the head neurons. The intensities were subsequently normalized to obtain $\Delta F/F$.

We analyzed sleep bouts in movies with 3 hr recording time. To score for sleep, the velocity data was smoothed using a 1st degree polynomial local regression model over 25 time points using the in-build *smooth* function in MATLAB. To be scored as a sleep bout, it had to last at least 3min with a smoothed velocity below 0.5μm/s for adults and dauers and below 3μm/s for arrested L1 larvae. In L1 larvae in the presence of food, a sleep bout was defined as a smoothed locomotion velocity below 1.2 μm/s for at least 2 min. To be scored as sleep using frame subtraction, intensity counts had to be below 20% of the average intensity. These cutoff parameters were determined empirically [95]. For sleep amount comparisons the time spent in a sleep bout was divided by the total time. For developing L1 larvae in the presence of food, lethargus was defined by the non-pumping period. As a representative L1 wake phase we took 3 hr before lethargus onset.

For averaging RIS activity and velocity, data was aligned to the sleep bout onset. RIS activity and velocity starting 15min before bout onset until 30min after sleep bout onset were selected for display. To obtain a flat baseline, selections that have sleeping bouts within the 15min before the bout onset were excluded from the alignment. The averaged RIS activities 5min before and 5min after the bout onset were compared using the Wilcoxon signed rank test for paired samples. The displayed RIS $\Delta F/F$ data of individual animals was smoothed with a 5-point-running-average.

Lifespan and survival assays

To isolate L1 larvae for lifespan and survival assays, eggs were synchronized using bleaching of a mixed population of worms and collected in standard M9 buffer [96]. The worms were allowed to hatch and to arrest overnight. The animals were then kept in the dark, at 20°C on a slowly spinning rotator in 2 mL Eppendorf tubes. At defined time intervals, we took samples and transferred the worms to fresh seeded NGM plates. We scored after 5-20 min the percentage of living worms to measure survival. A worm was scored as alive, when it was able to move on the plate. To analyze recovery, we kept the worms on the plates for 2-4 days at 20°C and then scored the percentage of worms that had re-entered development and had grown to at least larval stage L3 after feeding. At least 50 worms were scored at each time point. For comparisons, the time points “50% alive” and “50% recovering” were defined. These values represent the days when the survival/recovering curves reach 50% surviving or recovering worms [65].

For survival measurements of arrested L1s in the presence of food, FUdR was used to cause the arrest. FUdR was added to a final concentration of 50μM to the synchronized worm culture in M9 at 20°C at day 4. At day 5, at least 100 worms were pipetted to NGM plates seeded with *E. coli* and containing 50μM of FUdR, with a worm density of roughly 20 worms per plate. We kept the plates at 20°C in dark and scored every two or three days for viability. Worms were identified as dead if they didn’t shown locomotion after several gentle mechanical stimulations by pick. Dead worms were removed from the plates. Living worms were gently transferred to fresh FUdR NGM plates if any fungi contamination appeared on the plates. Worms that had dried out after crawling off the agar surface were excluded from the analysis.

For adult lifespan assays on food, a standard lifespan protocol was used [64]. In brief, a synchronized worm culture was grown until young adulthood. At least 50 worms were transferred to freshly seeded NGM plates with a worm density of roughly 10 worms per plate. Worms were kept at 20°C in dark and scored every two or three days for viability. They were classified as alive if they showed locomotion or, if quiescent, responded to gentle mechanical stimulation exerted by a platinum wire pick. Living worms were transferred to fresh NGM plates every two days in the first two weeks to separate them from their offspring until they had stopped laying eggs. Worms that had dried out after crawling off the agar surface or that died after leaking out through the vulva were excluded from the analysis. The lifespan assays of starving adults were performed similar to the lifespan assay on food. The differences were that young adults were kept to feed on plates supplemented with 50μM FUdR for two days before they were transferred to food-free plates containing again 50μM FUdR.

To correlate sleep amount with survival, we kept arrested larvae inside agarose microchambers without food at 20°C in dark. The microchambers with L1 larvae were prepared as described above. When the agar appeared dry after a few days, we re-moisturized the microchamber with 20μL of S-Basal containing 100u/mL Nystatin (Sigma Aldrich). Nystatin was added to prevent fungal growth. Worms were imaged after two days of arrest using DIC time-lapse microscopy with a frame rate of 0.1frame/s to quantify sleep. Using a home-made MATLAB script, the worm’s centroid was determined and its velocity measured. The resulting plot was smoothed over 40 time points. To be scored sleeping, the worm’s smoothened velocity had to be below 40% of the average velocity for at least 2min. The time spent sleeping was divided by the total imaging time, averagely for each worm from more than 35 hr of time lapse footage recorded between day 2 and day 4 after starvation. A linear fit was calculated from worms within the range: $\text{mean}_{(lifespan)} \pm \text{SD}_{(lifespan)}$. The others were excluded as outliers.

Quantification of bacteria consumption

To quantify bacterial consumption in FUdR-arrested larvae, FUdR was added to a final concentration of 50μM to the 3day synchronized worm culture for 24 hr. Worms were transferred in agarose microchambers filled with OP50 that had been transformed with a GFP plasmid (pFPV25.1) [34, 35, 80]. Food consumption was measured by quantifying GFP fluorescence over time. As a control we used bacteria-filled chambers that did not contain worms. With standard GFP filter sets, 20x objective, 490 nm light (with 2.00 mW/mm^2 intensity), 5ms exposure time and 50 EM gain we quantified GFP signals one and three days after microchamber

preparation. For statistical comparisons (two-sample t test), we averaged the pixel intensities of the chambers and calculated the change in fluorescent intensity between day one and three.

To compare bacterial consumption of FUdR-arrested and reproductive growing L1s, worms were cultured in agarose microchambers as described above. Food consumption was measured by quantifying GFP fluorescence with 10x objective over 200 frames (1 frame/min) at the 4-day old arrested L1. At this time, worms had approximately similar body sizes. To determine the body size of L1s, we assumed an elongated cylinder shape of the worm. The volume of each worm was then calculated from the body length and the radius of the worm [97]. For statistical comparison (two-sample t test), we normalized the fluorescence intensity change during the first 200 frames of each chamber to the corresponding mean worm volume.

Quantification of aging markers

Mitochondrial fission, poly-Q35 aggregation, and muscle morphology was quantified with fluorescence microscopy similar to what was previously described [65]. L1 arrested larvae (n = 10-15) were imaged every two to three days with a spinning disc fluorescence microscope equipped with a 488nm laser (Andor Revolution on Nikon TiE). Imaging was performed after immobilization with 25mM Levamisole and mounting of the worms between a sandwich of a coverslip and a 1 mm-thick 4% agarose in M9 pad [94]. Images were acquired and scored using Andor IQ Software. Imaging conditions for the Andor iXon camera were an EM gain of 100 and exposure time of 15-25ms. Laser power was adjusted for each condition. Using 100x objective or 60x objective, z stacks were recorded to image all focal planes of each worm. To score morphology of muscle fibers and mitochondria, body muscle cells in two of DL, DR, VL, VR bundles (in the head or body for fibers or mitochondria, respectively) were analyzed by scrolling through all stacks. First, 50 striated myofilaments in head were scored manually if they were defective (i.e., showed the morphological disruption or disorganization) [98]. Mitochondria were classified manually into three fission levels: tubular, intermediate fragmentation, or complete fragmentation as in a previous study [65]. The number of poly-Q35 aggregates was also scored manually for each worm by scrolling through all z stacks. Similar to how aggregates were scored in previous studies, we defined an aggregate as a discrete structure that is isolated from the surrounding fluorescence with a discernible boundary and that falls within a typical size between 1 to 5 μm [65, 67]. All aging marker experiments were replicated twice. Two independent observers, which were not blinded to the genotype, and who agreed on the final numbers, scored each experiment.

QUANTIFICATION AND STATISTICAL ANALYSIS

Quantification is described in the Method Details. Statistical tests used were Student's t tests (two-sided), Mann-Whitney U test, Wilcoxon rank tests for paired samples, log-rank tests and Fisher's exact test using Origin software or MATLAB. For multiple comparisons in Figures 4 (multiple genotypes) and S4 (multiple tissue-specific rescues) significance was confirmed by using the Benjamini-Hochberg Procedure with a false discovery rate of 5%. The specific tests used are described in the figure captions and the Results. The graphs show mean ± SEM unless noted otherwise. The boxplots show individual data points, the box represents the 25%–75% range, the whiskers the 10%–90% range, the thin gray line is the median and the bold black line is the mean. For each experiment at least two biological replicates were performed. Detailed results for lifespan and survival assays can be found in Tables S1–S5. A detailed list of the numbers of replicates for each experiment can be found in Table S6.

Current Biology, Volume *28*

Supplemental Information

Sleep Counteracts Aging Phenotypes to Survive Starvation-Induced Developmental Arrest in *C. elegans*

Yin Wu, Florentin Masurat, Jasmin Preis, and Henrik Bringmann

Figure S1

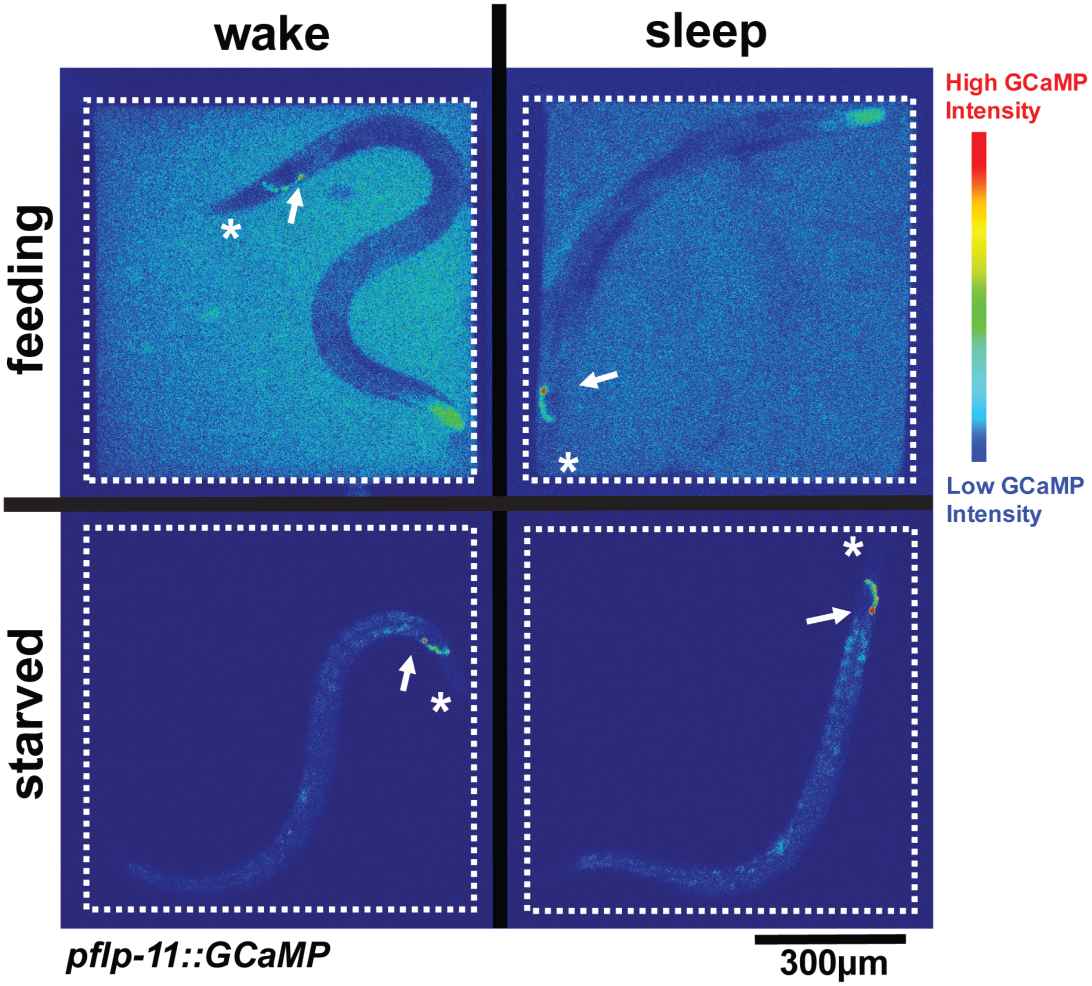
wake
sleep
feeding
starved
High GCaMP Intensity
Low GCaMP Intensity
pflp-11::GCaMP
300µm

Figure S1. Calcium and behavioral imaging in agarose microchambers. Related to Figure 1 and Figure 2. Shown are fluorescence images of adult worms expressing GCaMP3 in the sleep-active neuron RIS. The pictures are false-colored to visualize calcium activity. The border of the agarose microchamber is marked by a white dotted line. The asterisk indicates the worm's nose, the arrow the sleep neuron RIS. The left panels show wake worms, the right panels sleeping worms. Note the relaxed body posture and increased RIS GCaMP intensity during sleep. The top panels show worms feed on OP50. The bacteria are visible due to their auto-fluorescence. On the bottom panels, the worms are starved.

Figure S2

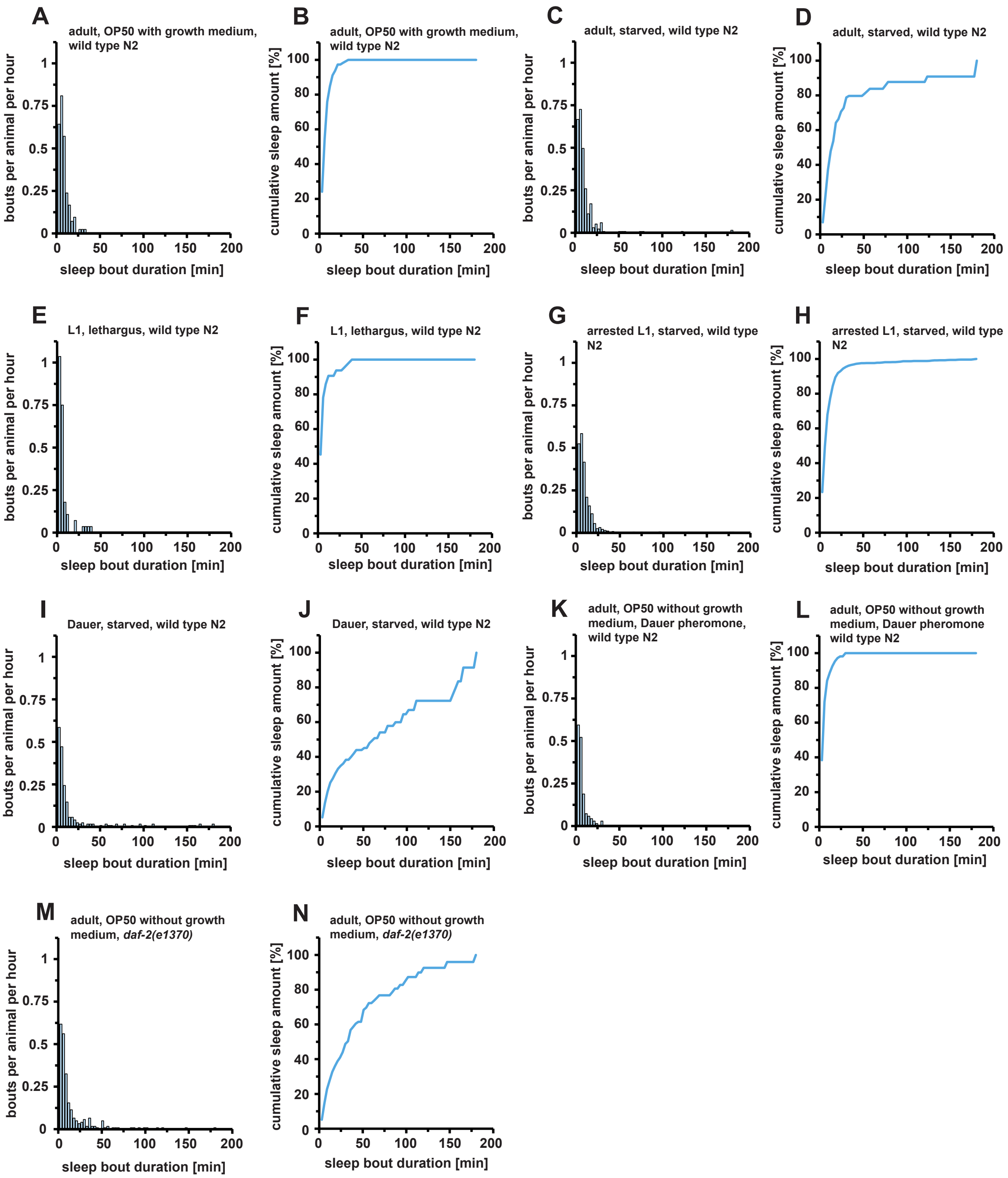

A
adult, OP50 with growth medium, wild type N2
bouts per animal per hour
sleep bout duration [min]
B
adult, OP50 with growth medium, wild type N2
cumulative sleep amount [%]
sleep bout duration [min]
C
adult, starved, wild type N2
bouts per animal per hour
sleep bout duration [min]
D
adult, starved, wild type N2
cumulative sleep amount [%]
sleep bout duration [min]
E
L1, lethargus, wild type N2
bouts per animal per hour
sleep bout duration [min]
F
L1, lethargus, wild type N2
cumulative sleep amount [%]
sleep bout duration [min]
G
arrested L1, starved, wild type N2
bouts per animal per hour
sleep bout duration [min]
H
arrested L1, starved, wild type N2
cumulative sleep amount [%]
sleep bout duration [min]
I
Dauer, starved, wild type N2
bouts per animal per hour
sleep bout duration [min]
J
Dauer, starved, wild type N2
cumulative sleep amount [%]
sleep bout duration [min]
K
adult, OP50 without growth medium, Dauer pheromone, wild type N2
bouts per animal per hour
sleep bout duration [min]
L
adult, OP50 without growth medium, Dauer pheromone wild type N2
cumulative sleep amount [%]
sleep bout duration [min]
M
adult, OP50 without growth medium, daf-2(e1370)
bouts per animal per hour
sleep bout duration [min]
N
adult, OP50 without growth medium, daf-2(e1370)
cumulative sleep amount [%]
sleep bout duration [min]

Figure S2. Distribution of sleep bout lengths and their contribution to total sleep amount. Related to Figure 1 and Figure 2. Shown are number of bouts per animal per hour as well as cumulative sleep amount as a function of bout length to show which bout lengths contribute how much to total sleep amount. Note that most sleep occurs in shorter bouts except for dauer larvae and *daf-2(-)*, which show also longer sleep bouts that contribute substantially to total sleep amount. (**A-B**) Adults with growth medium, (**C-D**) starved adults, (**E-F**) L1 lethargus, (**G-H**) starved L1 arrested, (**I-J**) dauer without food, (**K-L**) adults feeding on starved bacteria and dauer pheromone, (**M-N**) *daf-2(-)* adults feeding on starved bacteria.

Figure S3

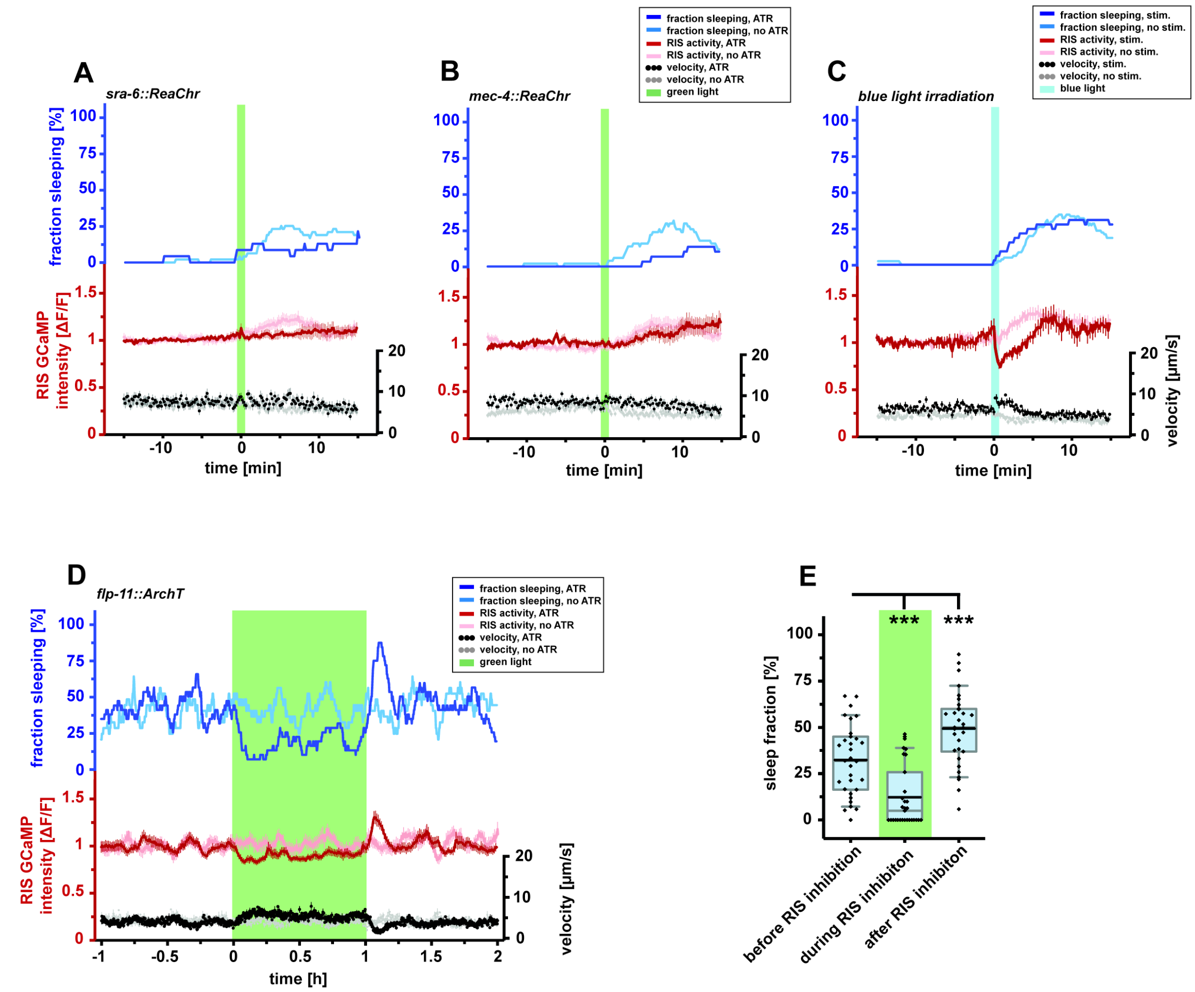

A
sra-6::ReaChr
B
mec-4::ReaChr
C
blue light irradiation
fraction sleeping, ATR
fraction sleeping, no ATR
RIS activity, ATR
RIS activity, no ATR
velocity, ATR
velocity, no ATR
green light
fraction sleeping, stim.
fraction sleeping, no stim.
RIS activity, stim.
RIS activity, no stim.
velocity, stim.
velocity, no stim.
blue light
fraction sleeping [%]
RIS GCaMP intensity [ΔF/F]
velocity [µm/s]
time [min]
D
flp-11::ArchT
time [h]
E
sleep fraction [%]
***
***
before RIS inhibition
during RIS inhibiton
after RIS inhibiton

Figure S3. Control experiments for characterizing larval sleep during starvation-induced arrest. Related to Figure 3. (**A-B**) ASH and mechano-sensory neurons activation during wake in arrested L1 larvae. During wake, there is no detectable action from ASH nociceptive neurons (**A**) or *mec-4* expression mechano-sensory neurons (**B**). RIS activity is shown in red (control without ATR in light red), speed in black (control without ATR in gray), and the fraction of sleeping animals in blue (control without ATR in light blue). (**A-C**) ASH and mechano-sensory neurons activation during wake in arrested L1 larvae. During wake, there is no detectable action from ASH nociceptive neurons (**A**), *mec-4* expression mechano-sensory neurons (**B**) or from noxious blue light irradiation (**C**). RIS activity is shown in red (control without ATR or without stimulation in light red), speed in black (control without ATR or without stimulation in gray), the fraction of sleeping animals in blue (control without ATR or without stimulation in light blue), green light illumination in green and noxious blue light irradiation in teal. (**D-E**) *flp-11::ArchT* activation efficiently reduces RIS activity and consequently inhibits sleep. (**D**) Complete documentation of homeostasis experiment. RIS activity is shown in red (control without ATR in light red), speed in black (control without ATR in gray), and the fraction of sleeping animals in blue (control without ATR in light blue). (**E**) Statistical comparison of the different 1h long periods. During green light illumination the sleep fraction was reduced by $62.0 \pm 0.5\%$, $^{***}p < 0.001$, and after illumination the sleep fraction increased by $52.5 \pm 0.1\%$, $^{***}p < 0.001$, compared to the sleep fraction before the illumination. Comparisons were made using the paired Wilcoxon rank test. $n = 32$ worms.

Figure S4

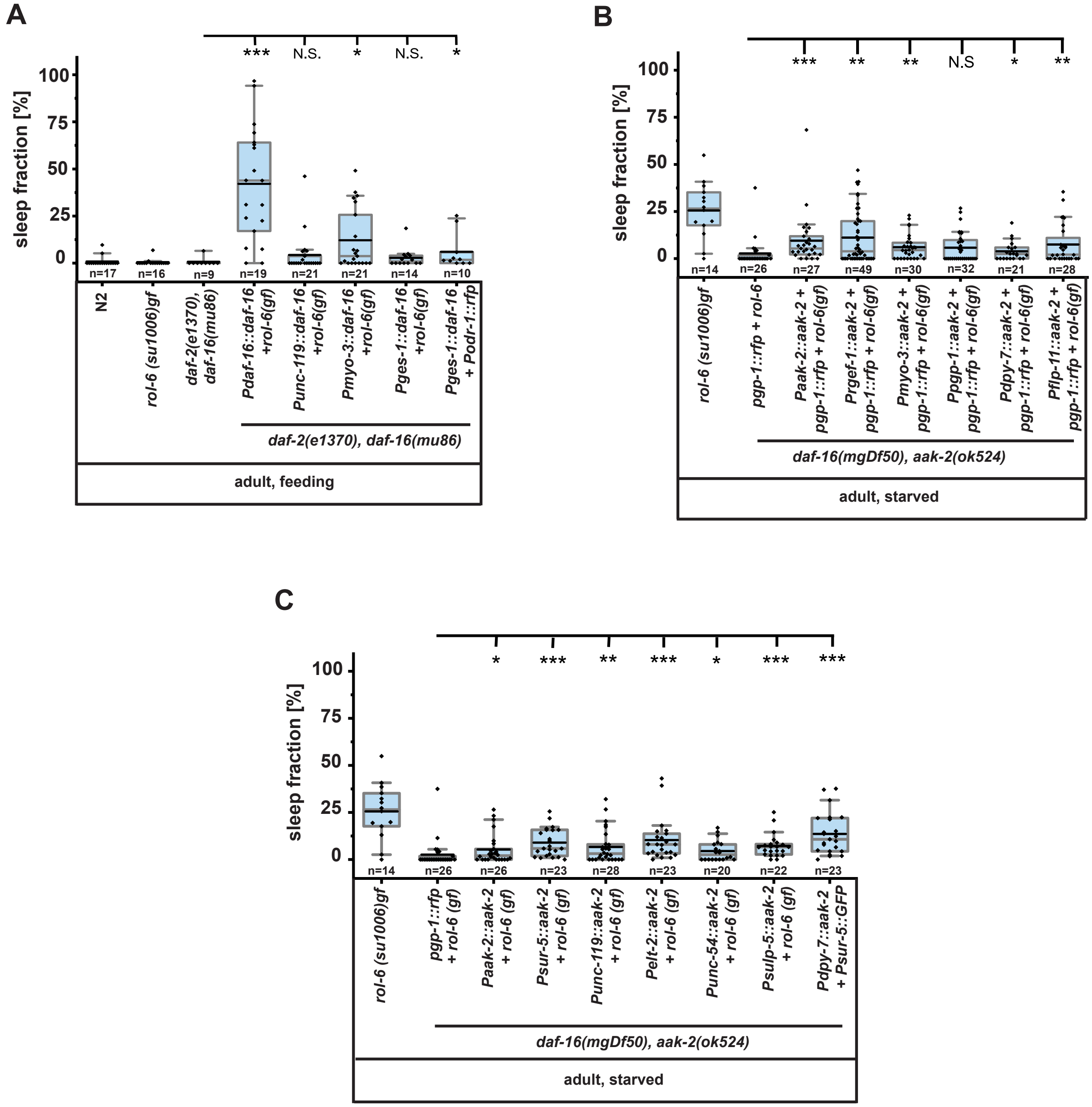

Figure S4. Rescue experiments for FoxO and AMPK. Related to Figure 4. (A) Rescue experiments using inserted transgenes of *daf-16* shows weak rescue only in muscle and inconsistent recue in the intestine. *daf-16* was expressed from its endogenous promoter, in neurons (*Punc-119*), in body wall muscle (*Pmyo-3*) and in the intestine (*Pges-1*). Most transgenes carry a *rol-6(su1006)* transformation marker, the roller phenotype does not affect sleep, Median sleep fraction was 0% for wild type N2 and *rol-6(su1006)*, the same data is displayed in panel B. Median sleep fraction for rescue experiments was 0% for the background genotype *daf-2(e1370)/daf-16(mu86)*, 44% for rescue using the endogenous promoter (***p < 0.001), 0 % for pan-neuronal rescue, 4% for muscle rescue (*p < 0.05), 1% and 2% for intestinal rescue dependent on the

transgenic array ($p = 0.11$ and $*p < 0.05$, respectively). *daf-2(e1370)/daf-16(mu86)* data was taken from DIC movies collected for the same animals that were analyzed for Figure 4C and is the same as used in Figure S4B. (B) Rescue experiments using extrachromosomal transgenes of *aak-2* shows weak rescue across tissues, which was expressed from its endogenous promotor, in neurons (*Prgef-1*), in body wall muscle (*Pmyo-3*), in the intestine (*Ppgp-1*), in the hypodermis (*Pdpy-7*), and in RIS (*Pflp-11*). Transgenes carry a *rol-6(su1006)* and intestinal red fluorescence (*pgp-1::rfp*) transformation marker. For comparison, a wild type background is shown, which only expresses a transformation marker. It is the same comparison as shown in Figure S4D. Median sleep fraction was 0% for *daf-2(mgDf50)/aak-2(ok524)* carrying the transformation markers only, 5% for rescue by endogenous promoter ($***p < 0.001$), 4% for pan-neuronal rescue ($**p < 0.01$), 4% for muscle rescue ($**p < 0.01$), 0% for intestine, 2% for hypodermis ($*p < 0.05$), and 2% for RIS ($**p < 0.01$). (C) Additional rescue experiments using extrachromosomal transgenes of *aak-2* with different transformation markers and promoters shows weak rescue across tissues, which was expressed from its endogenous promotor, ubiquitously (*Psur-5*), in neurons (*Punc-119*), in the intestine (*Pelt-2*), in muscle (*Punc-54*), in the hypodermis (*Pdpy-7*), and in the excretory cell (*Psulp-5*). Transgenes carry a *rol-6(su1006)* or other transformation marker. For comparison, a wild type background is shown, which only expresses a transformation marker. It is the same comparison as shown in Figure S4C. Median sleep fraction was 0% for *daf-2(mgDf50)/aak-2(ok524)* carrying the transformation markers only, 2% for rescue by the endogenous promoter ($*p < 0.001$), 6% for ubiquitous expression ($***p < 0.001$), 3% for pan-neuronal rescue ($**p < 0.01$), 8% for intestine ($***p < 0.001$), 3% for muscle rescue ($*p < 0.05$), 7% for the excretory cell ($***p < 0.001$) and 11% for hypodermis ($***p < 0.001$). The low rescue effects obtained after using the endogenous *aak-2* and *sur*-5 promoters are consistent with previous measurements using these transgenes. Perhaps the weak rescue can be explained by weak transmission of the array[S1]. The number of worms assayed (n) is displayed underneath each box plot. For statistical comparisons, Mann-Whitney U test was used to calculate the p value and significance was confirmed by Benjamini-Hochberg Procedure for multiple comparisons.

Figure S5

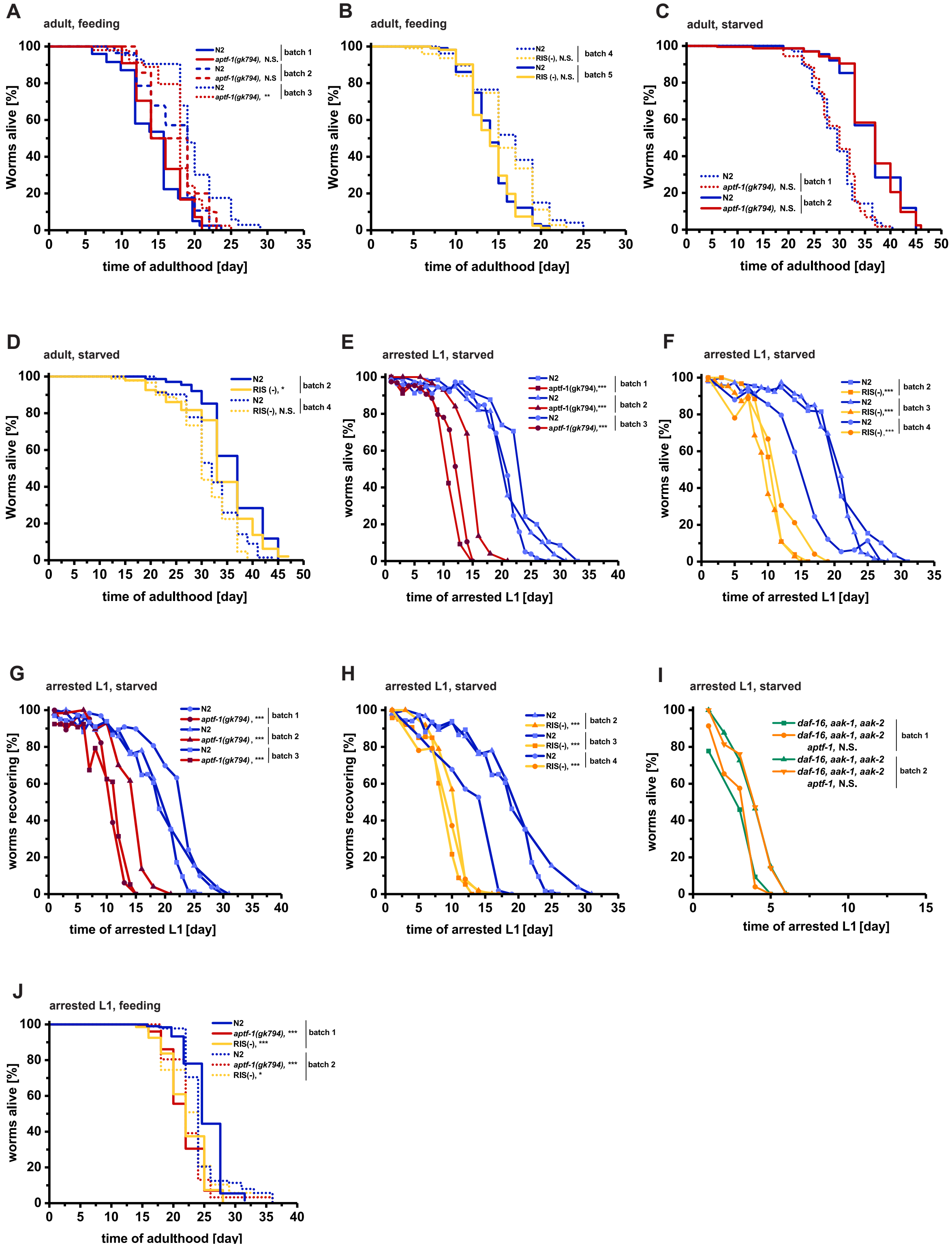
A
adult, feeding
N2
aptf-1(gk794), N.S.
batch 1
N2
aptf-1(gk794), N.S
batch 2
N2
aptf-1(gk794), **
batch 3
Worms alive [%]
time of adulthood [day]
B
adult, feeding
N2
RIS(-), N.S.
batch 4
N2
RIS (-), N.S.
batch 5
Worms alive [%]
time of adulthood [day]
C
adult, starved
N2
aptf-1(gk794), N.S.
batch 1
N2
aptf-1(gk794), N.S.
batch 2
Worms alive [%]
time of adulthood [day]
D
adult, starved
N2
RIS (-), *
batch 2
N2
RIS(-), N.S.
batch 4
Worms alive [%]
time of adulthood [day]
E
arrested L1, starved
N2
aptf-1(gk794),***
batch 1
N2
aptf-1(gk794),***
batch 2
N2
aptf-1(gk794),***
batch 3
worms alive [%]
time of arrested L1 [day]
F
arrested L1, starved
N2
RIS(-),***
batch 2
N2
RIS(-),***
batch 3
N2
RIS(-),***
batch 4
worms alive [%]
time of arrested L1 [day]
G
arrested L1, starved
N2
aptf-1(gk794) , ***
batch 1
N2
aptf-1(gk794) , ***
batch 2
N2
aptf-1(gk794) , ***
batch 3
worms recovering [%]
time of arrested L1 [day]
H
arrested L1, starved
N2
RIS(-), ***
batch 2
N2
RIS(-), ***
batch 3
N2
RIS(-), ***
batch 4
worms recovering [%]
time of arrested L1 [day]
I
arrested L1, starved
daf-16, aak-1, aak-2
daf-16, aak-1, aak-2
aptf-1, N.S.
batch 1
daf-16, aak-1, aak-2
daf-16, aak-1, aak-2
aptf-1, N.S.
batch 2
worms alive [%]
time of arrested L1 [day]
J
arrested L1, feeding
N2
aptf-1(gk794), ***
RIS(-), ***
batch 1
N2
aptf-1(gk794), ***
RIS(-), *
batch 2
worms alive [%]
time of adulthood [day]

Figure S5. Sleep is required to survive larval starvation. Related to Figure 5. Supplementary figure containing additional replicates related to Figure 5. The biological replicates are indicated as different batches with different lines or symbols. **(A)** Adult lifespan of feeding wild type (blue) and *aptf-1* mutants (red). No consistent significant difference could be shown. **(B)** Lifespan of feeding wild type (blue) and RIS(-) (yellow) adult worms were not significantly different for the two replicates. **(C)** No significant difference was detected for the starved adult lifespan of wild type and *aptf-1(gk794),* **(D)** and also not between starved wild type and RIS(-) adult worms. **(E-F)** L1-arrested starved *aptf-1(gk794)* mutants and RIS ablated worms show substantially reduced survival in all replicates latest from day 16. **(G-H)** All replicates of arrested L1 *aptf-1(gk794)* and RIS-ablated worms showed a significant decline in the ability to re-enter development compared to N2 worms when fed. Data are significant latest from day 12. **(I)** Starved arrested L1 *aak-1(tm1944)/aak-2(ok524)/daf-16(mgDf50)/aptf-1(gk794)* quadruple mutants showed no reduced lifespan compared to *aak-1(tm1944)/aak-2(ok524)/daf-16(mgDf50)* triple mutants. **(J)** *aptf-1(gk794)* and RIS(-) L1s arrested on FUdR have a decreased survival compared to wild type worms in the presence of food. The corresponding data, significance values and tests for all replicates are shown in Tables S1-S5.

Figure S6

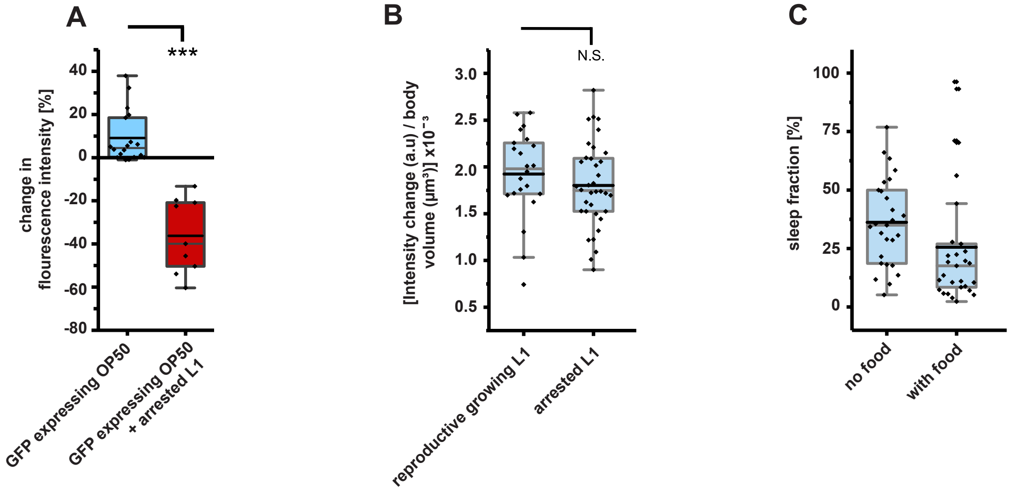

Figure S6. Arrested L1 larvae consume bacteria. Related to Figure 5. (**A**) The fluorescence intensity change of GFP expressing OP50 was measured in agarose microchambers. The two-day reduction in fluorescence is significantly different comparing microchambers containing arrest L1s to microchambers without worms (with worms: 36.3 ± 5.8% decrease, n = 9 microchambers, without worms: 9.1 ± 2.8% increase, n = 18 microchambers, ***p < 0.001, Mann-Whitney U test). **(B)** Arrested L1 larvae consume similar amounts of bacteria as reproductive growing L1 worms. The change in fluorescence intensity of OP50 GFP bacteria within 200 min was normalized to worm volume, to compensate for variability in size, which is caused by increased growth in non-arrested worms. The time window was chosen because size differences were still modest. No significant difference in bacterial consumption was detected for reproductive growing L1s (with a mean of 1.92×10^{-3} arbitrary units/μm^3, n = 22) and FUdR-arrested L1s (mean = 1.8×10^{-3} arbitrary units /µm3, n = 36), p = 0.33, two-sample t-test. (C) Sleep fraction of starved and feeding FUdR arrested L1s in wildtype. Starved L1s with FUdR (4 to 5 days-old) had a median sleep fraction of 34.9%, n = 27. Median sleep fraction of the feeding L1s with FUdR (4 to 5 days-old) is 17.5%, n = 29.

Table S1

Lifespan of adult, feeding					
Strain	Experiment batch (in Figure)	# scored/ # total	Mean of lifespan (days)	Compared to	*p* value against control (log-rank test)
wildtype N2	1 (Figure 5A)	44/50	14.64		
aptf-1(gk794)	1 (Figure 5A)	43/50	15.29	N2 Batch1	0.71, N.S.
wildtype N2	2	28/50	16.92		
aptf-1(gk794)	2	12/50	16.91	N2 Batch2	0.70, N.S.
wildtype N2	3	39/50	19.74		
aptf-1(gk794)	3	42/50	17.82	N2 Batch3	0.003, **
wildtype N2	4	72/100	16.30		
RIS(-)	4	75/102	15.54	N2 Batch4	0.23, N.S.
wildtype N2	5 (Figure 5B)	94/120	14.22		
RIS(-)	5 (Figure 5B)	86/120	14.07	N2 Batch5	0.71, N.S.
Lifespan of adult, starved					
wildtype N2	1 (Figure 5C)	60/100	29.81	-	
aptf-1(gk794)	1 (Figure 5C)	63/100	29.46	N2 Batch1	0.65, N.S.
wildtype N2	2	52/200	36.21		
aptf-1(gk794)	2	94/200	36.33	N2 Batch2	0.96, N.S.
RIS(-)	2	83/200	33.62	N2 Batch2	0.04, *
wildtype N2	3 (Figure 5D)	85/100	31.96		
RIS(-)	3 (Figure 5D)	84/95	30.64	N2 Batch3	0.06, N.S.

Table S1. Overview of all replicates of the adult lifespan. Related to Figure 5. Displayed are the strains used, the experimental / replicates, the scored animals from the total number (the missing worms were censored), the mean lifespan estimated from the log-rank test, against which wild type data was tested and the *p* value of the log-rank test.

Table S2

Survival of arrested L1, starved						
Strain	Experiment batch (in Figure)	min. scored/counting	50% alive at (days)	Compared to	Δ 50% alive	*p* value against control (Fisher's exact test)
wildtype N2	1	>50	22.89			
aptf-1(gk794)	1	>50	10.62	N2 Batch1	-53.6%	< 0.001, *** from day 11
wildtype N2	2	>50	20.06			
aptf-1(gk794)	2	>50	14,69	N2 Batch2	-26.8%	< 0.001, *** from day 16
RIS(-)	2	>50	10.3	N2 Batch2	-48.7%	< 0.001, *** from day 10
wildtype N2	3 (Figure 5E)	>50	20.70			
aptf-1(gk794)	3 (Figure 5E)	>50	12.07	N2 Batch3	-41.7%	< 0.001, *** from day 11
RIS(-)	3 (Figure 5E)	>50	9.41	N2 Batch3	-54.5%	< 0.001, *** from day 9
wildtype N2	4	>50	14.85			
RIS(-)	4	>50	9.93	N2 Batch4	-33.1%	< 0.001, *** from day 12

Table S2. Overview of all replicates of the starved arrested L1 lifespans. Related to Figure 5. Displayed are the strains used, the batch / replicate, the scored worms, median survival, against which wild type data was tested, the reduction in lifespan compared to wildtype and the *p* value and from which day on there was a significant difference in the Fisher's exact test.

Table S3

Ability to re-enter development of arrested L1 when fed, starved						
Strain	Experiment batch (in Figure)	min. scored/counting	50% recover at (days)	Compared to	Δ 50% alive	*p* value against control (Fisher's exact test)
wildtype N2	1	>50	22.68			
aptf-1(gk794)	1	>50	10.49	N2 Batch1	-53.7%	< 0.001, *** from day 11
wildtype N2	2	>50	19.42			
aptf-1(gk794)	2	>50	14.48	N2 Batch2	-25.4%	< 0.001, *** from day 16
RIS(-)	2	>50	10.28	N2 Batch2	-47.1%	< 0.001, *** from day 10
wildtype N2	3 (Figure 5F)	>50	18.74			
aptf-1(gk794)	3 (Figure 5F)	>50	11.37	N2 Batch3	-39.3%	< 0.001, *** from day 12
RIS(-)	3 (Figure 5F)	>50	8.53	N2 Batch3	-54.5%	< 0.001, *** from day 8
wildtype N2	4	>50	14.12			
RIS(-)	4	>50	9.09	N2 Batch4	-35.6%	< 0.001, *** from day 12

Table S3. Overview of all replicates of the capacity to recover from the L1 arrest. Related to Figure 5. Displayed are the strains used, the experimental batch / replicate, the scored animals, median recovery rate, against which wild type data set was tested, the reduction in lifespan compared to wildtype and the *p* value and from which day on there was a significant difference in the Fisher's exact test.

Table S4

Survival of arrested L1 of quadruple mutants, starved					
Strain	Experiment batch (in Figure)	min. scored/counting	50% alive at (days)	Compared to	*p* value against control (Fisher's exact test)
aak-1(tm1944), aak-2(ok524), daf-16(mgDf50)	1	>60	2.74		
aak-1(tm1944), aak-2(ok524), daf-16(mgDf50), aptf-1(gk794)	1	>60	3.14	Triple mutant Batch1	>0.05 N.S.
aak-1(tm1944), aak-2(ok524), daf-16(mgDf50)	2 (Figure 5H)	>60	3.85		
aak-1(tm1944), aak-2(ok524), daf-16(mgDf50), aptf-1(gk794)	2 (Figure 5H)	>60	3.9	Triple mutant Batch2	>0.05 N.S.

Table S4. Overview of all replicates of the lifespan of IIS and AMPK mutants. Related to Figure 5. Overview of all replicates of the lifespan of IIS and AMPK mutants. Displayed are the strains used, the experimental batch / replicate, the scored animals, median recovery rate, against which wild type data set was tested, the reduction in lifespan compared to wildtype and the *p* values and significance in the Fisher's exact test.

Table S5

Survival of FUdR-arrested L1, feeding						
Strain	Experiment batch (in Figure)	scored/total	Mean of lifespan (days)	Compared to	Δ 50% alive	*p* value against control (log-rank test)
wildtype N2	1 (Figure 5I)	143/200	25.70			
aptf-1(gk794)	1 (Figure 5I)	140/200	21.86	N2 Batch1	-14.5%	< 0.001, ***
RIS(-)	1 (Figure 5I)	135/200	22.05	N2 Batch1	-14.2%	< 0.001, ***
wildtype N2	2	88/101	24.63			
aptf-1(gk794)	2	92/101	22.5	N2 Batch2	-8.6%	< 0.001, ***
RIS(-)	2	100/144	23.04	N2 Batch2	-6.5%	0.017, *

Table S5. Overview of all replicates of the lifespan of FUdR-arrested L1s. Related to Figure 5. Displayed are the strains used, the experimental batch / replicate, the scored animals, the mean survival, against which wild type data set was tested, the reduction in lifespan compared to wildtype and the *p* values and the significance in the log-rank test.

Table S6

Experiment	No. of biological replicates	Comments
Figure 1J	4	
Figure 1K	7	
Figure 1L	3	
Figure 1M	7	
Figure 1N	5	
Figure 1O	3	
Figure 2A, wt with growth medium	4	
Figure 2A, *aptf-1(-)* with growth medium	4	
Figure 2A, wt without growth medium	2	
Figure 2A, *aptf-1(-)* without growth medium	2	
Figure 2A, wt with growth medium, dead OP50	3	
Figure 2A, *aptf-1(-)* with growth medium, dead OP50	2	
Figure 2B, wt, adult feeding	2	
Figure 2B, *aptf-1(-)*, adult feeding	2	
Figure 2B, *Pflp-11:EGL-1*, adult feeding	6	
Figure 2B, wt, adult starved	9	
Figure 2B, *aptf-1(-)*, adult starved	3	
Figure 2B, *Pflp-11:EGL-1*, adult starved	6	
Figure 2C, wt, non-starved L1, before lethargus	3	
Figure 2C, *aptf-1(-)*, non-starved L1, before lethargus	3	
Figure 2C, wt, non-starved L1, during lethargus	3	
Figure 2C, *aptf-1(-)*, non-starved L1, during lethargus	3	
Figure 2D, wt, arrested L1	8	
Figure 2D, *aptf-1(-)*, arrested L1	4	
Figure 2D, *Pflp-11:EGL-1*, arrested L1	2	
Figure 2E, wt, Dauer	8	
Figure 2E, *aptf-1(-)*, Dauer	4	
Figure 2E, *Pflp-11:EGL-1*, Dauer	4	
Figure 2F, wt, Dauer Pheromone	5	
Figure 2F, *aptf-1(-)*, Dauer Pheromone	3	
Figure 3A, B: 2	2	
Figure 3C with ATR	2	
Figure 3C without ATR	2	
Figure 3D with ATR	2	
Figure 3D without ATR	2	
Figure 3E with ATR	4	
Figure 3E without ATR	3	
Figure 3F	2	
Figure 4B	6	
Figure 4C wt	2	
Figure 4C *daf-2(-)*	9	
Figure 4C *daf-2(-); aptf-1(-)*	4	
Figure 4C *daf-2(-); daf-16(-)*	6	
Figure 4D wt	9	
Figure 4D *daf-16(-)*	4	
Figure 4D *aak-1(-); aak-2(-)*	7	
Figure 4D *daf-16(-); aak-1(-)*	4	
Figure 4D *daf-16(-); aak-2(-)*	2	
Figure 4D *daf-16(-); aak-1(-); aak-2(-)*	3	
Figure 4E wt	8	
Figure 4E *daf-16(-)*	2	
Figure 4E *aak-1(-); aak-2(-)*	2	
Figure 4E *daf-16(-); aak-1(-)*	2	
Figure 4E *daf-16(-); aak-2(-)*	4	
Figure 4E *daf-16(-); aak-1(-); aak-2(-)*	6	
Figure 4F wt	2	
Figure 4F *daf-18(-)*	2	
Figure 4F *sir2.1(-)*	2	
Figure 5A adult wt, feeding + Figure S5A	3	
Figure 5A adult *aptf-1(-)*, feeding + Figure S5A	3	
Figure 5B adult wt, feeding + Figure S5B	2	
Figure 5B adult RIS(-), feeding + Figure S5B	2	
Figure 5C adult wt, starved + Figure S5C	2	
Figure 5C adult *aptf-1(-)*, starved + Figure S5C	2	
Figure 5D adult wt, starved + Figure S5D	2	
Figure 5D adult RIS(-), starved + Figure S5D	2	
Figure 5E L1 wt, starved + Figure S5E-F	3	

Figure 5E L1 *aptf-1(-)*, starved + Figure S5E-F	3	
Figure 5E L1 RIS(-), starved + Figure S5E-F	3	
Figure 5F L1 wt, starved + Figure S5G-H	3	
Figure 5F L1 *aptf-1(-)*, starved + Figure S5G-H	3	
Figure 5F L1 RIS(-), starved + Figure S5G-H	3	
Figure 5G wt, starved	5	
Figure 5H *daf-16(-); aak-1(-); aak-2(-),* starved + Figure S5I	2	
Figure 5H *daf-16(-), aak-1(-), aak-2(-), aptf-1(-),* starved + Figure S5I	2	
Figure 5I L1 wt, FUDR, feeding + Figure S5J	2	
Figure 5I L1 *aptf-1(-)*, FUDR, feeding + Figure S5J	2	
Figure 5I L1 RIS(-), FUDR, feeding + Figure S5J	2	
Figure 6A wt	2	
Figure 6A *aptf-1(-)*	2	
Figure 6C wt	2	
Figure 6C *aptf-1(-)*	2	
Figure 6E wt	2	
Figure 6E *aptf-1(-)*	2	
Figure S2A	4	
Figure S2B	4	
Figure S2C	9	
Figure S2D	9	
Figure S2E	3	
Figure S2F	3	
Figure S2G	8	
Figure S2H	8	
Figure S2I	8	
Figure S2J	8	
Figure S2K	5	
Figure S2L	5	
Figure S2M	9	
Figure S2N	9	
Figure S3A with ATR	2	
Figure S3A without ATR	2	
Figure S3B with ATR	2	
Figure S3B without ATR	2	
Figure S3C, D with ATR	4	
Figure S3C, D without ATR	3	
Figure S3E, before RIS inhibition	4	
Figure S3E, during RIS inhibition	4	
Figure S3E, after RIS inhibition	4	
Figure S4A wt	2	From Figure 4C
Figure S4A *rol-6(gf)*	2	
Figure S4A *daf-2(-); daf-16(-)*	4	Reanalysis of DIC movies from Figure 4C
Figure S4A *daf-2(-); daf-16(-); Pdaf-16::daf-16+rol-6(gf)*	3	
Figure S4A *daf-2(-); daf-16(-); Punc-119::daf-16+rol-6(gf)*	5	
Figure S4A *daf-2(-); daf-16(-); Pmyo-3::daf-16+rol-6(gf)*	5	
Figure S4A *daf-2(-); daf-16(-); Pges-1::daf-16+rol-6(gf)*	4	
Figure S4B *rol-6(gf)*	2	
Figure S4B *daf-16(-); aak-2(-); pgp-1::rfp+rol-6 (gf)*	4	
Figure S4B*daf-16(-); aak-2(-); Paak-2::aak-2+pgp-1::rfp+rol-6 (gf)*	5	
Figure S4B *daf-16(-); aak-2(-); Prgef-1::aak-2+pgp-1::rfp+rol-6 (gf)*	8	
Figure S4B*daf-16(-); aak-2(-); Pmyo-3::aak-2+pgp-1::rfp+rol-6 (gf)*	5	
Figure S4B *daf-16(-); aak-2(-); Ppgp-1::aak-2+pgp-1::rfp+rol-6 (gf)*	5	
Figure S4B *daf-16(-); aak-(-); Pdpy-7::aak-2+pgp-1::rfp+rol-6 (gf)*	3	
Figure S4B *daf-16(-); aak-2(-); Pdpy-7::aak-2+pgp-1::rfp+rol-6 (gf)*	3	
Figure S4C *rol-6 (gf)*	2	
Figure S4C *daf-16(-); aak-2(-); pgp-1::rfp+rol-6 (gf)*	4	
Figure S4C *daf-16(-); aak-2(-); Paak-2::aak-2+rol-6 (gf)*	3	
Figure S4C *daf-16(-); aak-2(-); Psur-5::aak-2+rol-6 (gf)*	3	
Figure S4C *daf-16(-), aak-2(-), Punc-119::aak-2+rol-6 (gf)*	2	
Figure S4C *daf-16(-); aak-2(-); Pelt-2::aak-2+rol-6 (gf)*	3	
Figure S4C *daf-16(-); aak-2(-); Punc-54::aak-2+rol-6 (gf)*	3	
Figure S4C *daf-16(-); aak-2(-); Psulp-5::aak-2+rol-6 (gf)*	3	
Figure S4C *daf-16(-); aak-2(-); Pdpy-7::aak-2+Psur-5::GFP*	3	
Figure S5A, wt, adult feeding	3	
Figure S5A, *aptf-1*(-), adult feeding	3	
Figure S5B, wt, adult feeding	2	
Figure S5B, RIS(-), adult feeding	2	
Figure S5C, wt, adult starved	2	

Figure S5C, *aptf-1(-)*, adult starved	2	
Figure S5D, wt, adult starved	2	
Figure S5D, RIS(-), adult starved	2	
Figure S5E, wt, arrested L1 starved	3	
Figure S5E, *aptf-1(-)*, arrested L1 starved	3	
Figure S5F, wt, arrested L1 starved	3	
Figure S5F, RIS(-), arrested L1 starved	3	
Figure S5G, wt, arrested L1 starved	3	
Figure S5G, *aptf-1(-)*, arrested L1 starved	3	
Figure S5H, wt, arrested L1	3	
Figure S5H, RIS(-), arrested L1	3	
Figure S5I, *daf-16(-); aak-1(-); aak-2(-),* arrested L1	2	
Figure S5I, *daf-16(-); aak-1(-); aak-2(-); aptf-1(-)*, arrested L1	2	
Figure S5J, wt, arrested L1 feeding	2	
Figure S5J, *aptf-1(-)*, arrested L1 feeding	2	
Figure S5J, RIS(-), arrested L1 feeding	2	
Figure S6A GPF OP50 control	2	
Figure S6A GFP OP50 L1 arrested worm	2	
Figure S6B reproductive growing L1	2	
Figure S6B arrested L1	2	
Figure S6C FUDR arrested L1, starved	2	
Figure S6C FUDR arrested L1, feeding	2	

Table S6. Number of replicates for all experiments. Related to all Figures and Supplemental Figures.

Table S7

Primer name	Primer sequence 5'-3'
aak-1(tm1944)	
890	ACTGTTCCGAGTCCTTTCATTC
891	TTCCCTTTCTTCGCTCACTTTT
892	ATGGAACTGTGTATCGGCCA
aak-2(ok524)	
893	ATCGCCAAATTATGCTGCCC
894	GGCAGGGTTCCACAAAGAAG
895	AAGGAGACACTCGGAGTTGG
aptf-1(ok974)	
48	CGACAATCTTCCCAAAGACC
49	CGGATCGATTGCTAGAGAGG
50	GCTTGGACGGCTTTAGTTGA
*daf-2(e1370)**	
519	CGGGATGAGACTGTCAAGATTGGAGATTTCGG
520	CAACACCTCATCATTACTCAAACCAATCCATG
daf-16(mgDf50)	
636	AGGTCAGAGTTCTAGGCGTAAA
637	CGGTTCCAGCAATTCCAAGT
638	AGTGGATTCTGAGCACACGA
daf-16(mu86)	
468	AAAGAGCTCGTGGTGGGTTA
469	TCGCGCCTTTGTCTCTCTAT
470	AGTGTCGAGTGAAGGGAGC
sir-2.1(ok434)	
1107	GTTTGATCACTCCTTCGCAAC
1108	TCCCAGGACAGTTCGTACC
1109	GCGGAAATGGCAGAAGTAGT
daf-18(ok480)	
1090	AGCATGGACTTCAACGATACAA
1091	GAATGCCAGATTGCCGAACA
1092	CGGTGGTCCATTTGAGATACC

Table S7. List of PCR primers used for Duplex PCR Genotyping. Related to STAR Methods. *The *daf-2* mutant allele was identified using restriction with NcoI-HF (in CutSmart Buffer, New England Biolabs).

Supplemental Reference

S1. Narbonne, P., and Roy, R. (2009). Caenorhabditis elegans dauers need LKB1/AMPK to ration lipid reserves and ensure long-term survival. Nature *457*, 210-214.
